# The Surtsey Magma Series

**DOI:** 10.1038/srep11498

**Published:** 2015-06-26

**Authors:** C. Ian Schipper, Sveinn P. Jakobsson, James D.L. White, J. Michael Palin, Tim Bush-Marcinowski

**Affiliations:** 1 School of Geography, Environment and Earth Sciences, Victoria University, PO Box 600, Wellington 6140, New Zealand; 2 Icelandic Institute of Natural History, Urridaholtsstraeti 6-8, 210 Gardabaer, Iceland; 3 Geology Department, University of Otago, PO Box 56, Dunedin 9054, New Zealand

## Abstract

The volcanic island of Surtsey (Vestmannaeyjar, Iceland) is the product of a 3.5-year-long eruption that began in November 1963. Observations of magma-water interaction during pyroclastic episodes made Surtsey the type example of shallow-to-emergent phreatomagmatic eruptions. Here, in part to mark the 50^th^ anniversary of this canonical eruption, we present previously unpublished major-element whole-rock compositions, and new major and trace-element compositions of sideromelane glasses in tephra collected by observers and retrieved from the 1979 drill core. Compositions became progressively more primitive as the eruption progressed, with abrupt changes corresponding to shifts between the eruption’s four edifices. Trace-element ratios indicate that the chemical variation is best explained by mixing of different proportions of depleted ridge-like basalt, with ponded, enriched alkalic basalt similar to that of Iceland’s Eastern Volcanic Zone; however, the systematic offset of Surtsey compositions to lower Nb/Zr than other Vestmannaeyjar lavas indicates that these mixing end members are as-yet poorly contained by compositions in the literature. As the southwestern-most volcano in the Vestmannaeyjar, the geochemistry of the Surtsey Magma Series exemplifies processes occurring within ephemeral magma bodies on the extreme leading edge of a propagating off-axis rift in the vicinity of the Iceland plume.

The eruption of Surtsey Volcano, in the Vestmannaeyjar (Vestmann Islands), Iceland (1963–67; Fig. 1), is the canonical example of shallow-to-emergent subaqueous explosive volcanism. It demonstrated the ways in which magma-water interaction drastically affects plume and fragmentation dynamics to create characteristic steam-rich tephra jets and fine-grained deposits. The growth of new islands during the eruption was intensively chronicled in a near-continuous sample and observation record^1^,^2^,^3^,^4^,^5^ that helped it become the type example of pyroclastic activity that is now referred to as *Surtseyan Volcanism*^6^,^7^,^8^. Timing also probably contributed to Surtsey becoming a type locality, because its eruption occurred not long after similar ones (particularly of Capelinhos, Azores, 1957-58^9^,^10^) had demonstrated dynamic and depositional^11^ similarities, motivating volcanologists to consider that shallow phreatomagmatic eruptions deserved their own unique and unifying classification among eruption styles^12^. Beyond the physical dynamics demonstrated during Surtsey’s four-years of activity, the locality that is Surtsey Volcano is significant for having formed in the southwestern-most eruption of the Vestmannaeyjar archipelago, at the propagating tip of Iceland’s Pleistocene-Holocene, Eastern Volcanic Zone^13^ (EVZ). Its products are therefore key to unravelling petrological characteristics at the leading edge of an off-axis rift in the vicinity of the Iceland plume ^e.g.,^^14^.

Here we present the “Surtsey magma series”, defined by previously unpublished whole-rock major-element analyses of microcrystalline rocks (bombs and lava) from different vents, and the “Surtsey melt series”, defined by new and previously unpublished major and trace-element analyses of sideromelane glasses in tephra collected by observers and retrieved from the 1979 drill core^15^. Samples in both series are linked to their time of eruption and vent of origin to allow a spatiotemporal account of geochemical variability throughout the progression of Surtsey’s eruption, from both whole rocks (melt + crystals +/− inclusions) and glass (melt now preserved as glass in tephra, but having been subjected to some fractionation during the eruption) perspectives. Now, in the 50^th^ anniversary of Surtsey’s eruption, and with a recently submitted International Continental Drilling Program (ICDP) proposal to drill into Surtsey for a second time^16^, this paper serves as a short review of the eruption, an archive of new and previously unpublished geochemical data from valuable historical samples, and an assessment of petrological characteristics of Surtsey’s magma system.

## Progression of the Eruption

The crew of fishing vessel *Ísleifur II* were first to witness activity in the vicinity of what would become the island of Surtsey, at first mistaking the plume for the smoke of a burning ship, in the early morning of November 14, 1963^2^. Following their reports, a consortium of Icelandic geologists began documenting the eruption in great detail, with many aspects of the eruption given in the Surtsey Research Progress Reports (https://www.surtsey.is) and by S. Thorarinsson^1^,^2^,^3^,^4^,^5^. Detailed timelines of activity at Surtsey and its satellite vents are given by Jakobsson and Moore^15^ and Thordarson^17^, and are summarised below and in Fig. 2a.

**Figure 1:**
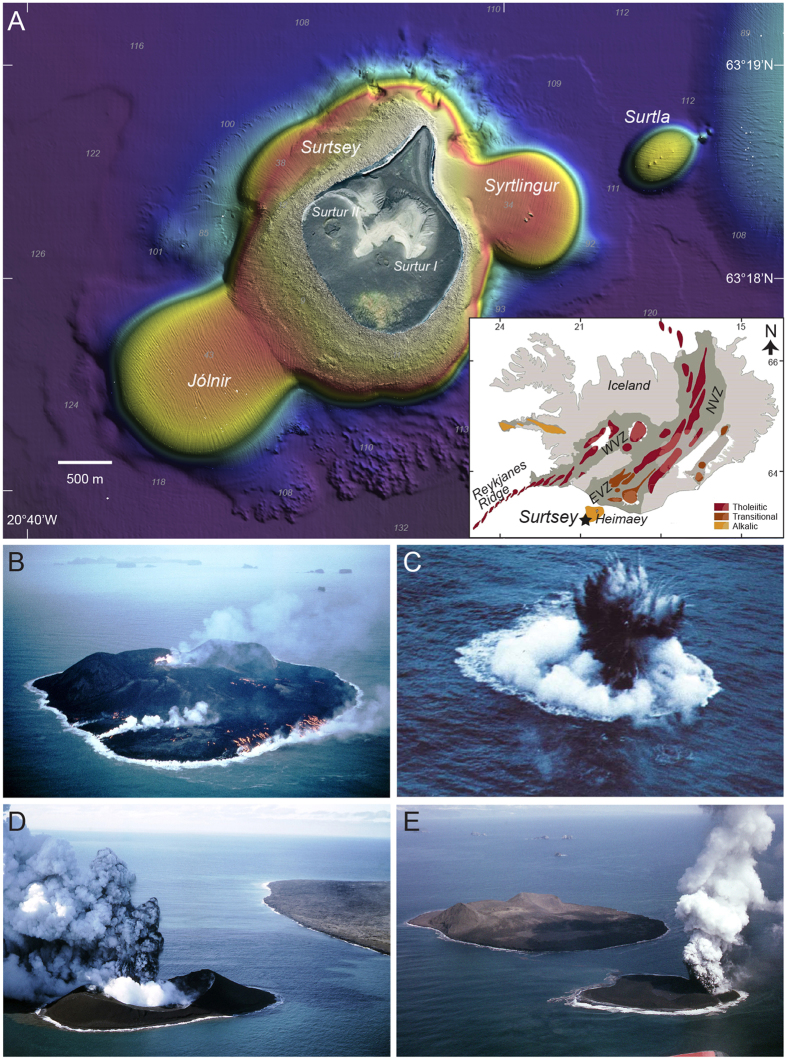
Location and eruptive activity at Surtsey **A**) Bathymetry of Surtsey as of 2007 multibeam survey, modified with permission from Jakobsson *et al*. 77. Inset shows outline of Iceland in the North Atlantic with Eastern (EVZ), Western (WVZ), Northern (NVZ), and Snaefellsnes (SnVZ) Volcanic Zones indicated^78^. B) Surtsey during effusive phase of *Surtur II* vent, 16 Oct. 1964. C) Pyroclastic activity at *Surtla*, 29 Dec. 1963; D-E) Pyroclastic activity at *Syrtlingur*, 1 Jul. 1965, and Jólnir, 15 Jul. 1966. Photographs by pilot Sigurjón Einarsson, reprinted with permission.

It is estimated that activity began on the seafloor in early November 1963, with a short submarine fissure building within a few days^15^ from a relatively flat ocean floor at about 130 metres below sea level, to the surface by 14 November. Pyroclastic activity from this initial vent, *Surtur I*, built the foundation of the island that remains today. On 28 December 1963, pyroclastic activity at a new submarine vent *Surtla* was identified ~2 km to the NE of Surtsey. *Surtla’s* phreatomagmatic jets breached the ocean surface from less than a metre’s depth (Fig. 1C)^2^, but the edifice was never observed above water, and its activity had ceased by 6 January 1964. At the end of January 1964, pyroclastic activity on Surtsey continued, but shifted to a new Western vent, *Surtur II*. Pyroclastic eruption of *Surtur II* continued until early April, 1964, when it entered an effusive phase (Fig. 1B), with a 120 m wide lava pond forming in the crater. Effusive activity continued until mid-May 1965, and included the establishment of various lava ponds and lava tube systems, intermittent surface flow activity, and the effusion of tube-fed submarine pillow lavas.

As effusive activity at *Surtur II* waned, submarine pyroclastic activity at the new satellite vent *Syrtlingur* (Fig. 1D) began ~600 m to the NE of Surtsey. *Syrtlingur* grew above the surface in late May 1965, and continued in intermittent activity until 17 October 1965. A lack of effusive activity at *Syrtlingur* left it subject to rapid wave erosion, and though it achieved a maximum height of ~70 metres above sea level, it was washed away within one week of activity ending. The same fate befell the satellite vent *Jólnir* (Fig. 1E), which is estimated to have began its submarine growth ~800 m to the SW of Surtsey when *Syrtlingur* was waning, and emerged from the sea surface in late December 1965. Activity at *Jólnir* continued until early August 1966, and was entirely pyroclastic. The tephra island of *Jólnir* was washed away by late October.

**Figure 2:**
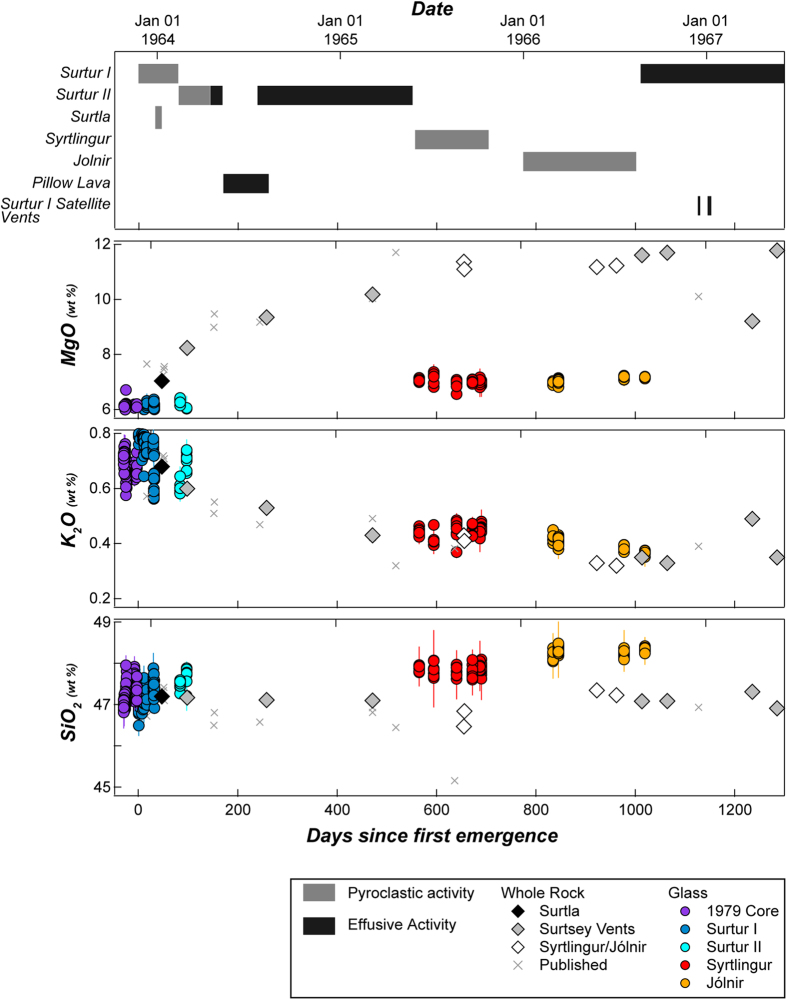
Time series of eruptive activity and overview of major-element variation. Grey crosses indicate previously published whole-rock analyses^5^,^13^,^22^,^25^,^28^,^31^. Glasses plotted as individual grains (Table S2), the averages of which are listed in Tables 3, 4. Eruption day for drill core glasses are not known, but for plotting purposes are assumed to have accumulated at a constant rate over 1 month up to the November 14, 1963 emergence, and are plotted as negative days. Actual estimates of time from breaching the seafloor to emergence are much shorter^15^.

*Jólnir’s* was the last pyroclastic activity at Surtsey Volcano. However, effusive activity resumed on the main island as *Jólnir* became inactive, from the original *Surtur I* vent. Mid August 1966 through to early June 1967 saw variable effusive activity in and around *Surtur I*, and the opening of several satellite vents in its vicinity. The extensive lava flows on the main island of Surtsey ensured its short-term resistance to erosion by wave action, long enough for palagonitization to cement the bulk of the *Surtur I and II* tephra cones into resistant tuffs^18^,^19^. In total, the eruption lasted 3.5 years (Fig. 2), and produced a total of 1.1–1.2 km^3^ of material (60–70% tephra), with 0.07 km^3^ at each of *Syrtlingur* and *Jólnir*, and 0.01 km^3^ at *Surtla*^15^.

## Previous studies on Surtsey

Surtsey has been legally protected since its emergence, and has become an important site for multidisciplinary science. Intensive studies, mainly in Earth Sciences, Terrestrial Biology and Marine Biology, provided the background for the successful nomination of Surtsey as a UNESCO World Heritage Site. The above headings are of course extremely broad, and a bibliography of works relating to Surtsey is maintained by the Surtsey Research Society (www.surtsey.is).

The petrography of Surtsey lava and/or tephra has been described in many papers and re-ports^2^,^5^,^13^,^18^,^20^,^21^,^22^,^23^,^24^,^25^,^26^,^27^,^28^. A few studies have concentrated specifically on plagioclase^29^ or oxide^21^,^26^ chemistry, and others have used Surtsey material for experimental investigation of melting relations^30^ and phase equilibria^22^,^23^. Jakobsson^28^ identified two magma types among the Vestmannaeyjar basalts, a parent VE-I and daughter VE-II. The VE-I type lavas are the most common at Surtsey, and have macrophenocrysts comprising (void-free) 12–17  vol.% olivine (often with Cr-spinel inclusions), 0–12 vol.% plagioclase, and no clinopyroxene. They have an additional microphenocryst population consisting of ≤2.2 vol.% labradorite and ≤0.7 vol.% Cr-spinel. VE-II type lavas have similar mineralogy, except for having more albitic plagioclase microphenocrysts. The crystalline groundmass of both lava types contains microlites of plagioclase, clinopyroxene, olivine, Fe-Ti oxides, with minor apatite and occasional nepheline and aegerine augite.

Sampling of Surtsey eruption products was systematic, but geochemical analyses have appeared sporadically in the literature (Fig. 2). The first report of whole-rock composition was for a single tephra erupted on Dec 1, 1964^5^,^24^. The addition of two additional analyses by Steinþórsson^25^ then showed a major-element trend from tephra more evolved at *Surtur* through to more primitive at *Syrtlingur*. Jakobsson presented additional analyses when defining the VE-I and VE-II magmas^13^,^28^; he noted that the latter was lower in MgO (VE-I: MgO = 9.1–10.1 wt% for those without olivine cumulates, VE-II: MgO = 5.4–8.2 wt%, Vestmannaeyjar wide, as per ref. 13) and was probably the fractionated daughter of the former. In 1981, scuba divers visited the eroded remnants of *Surtla* to collect three additional whole-rock samples^31^. Kokelaar and Durant^31^ found *Surtla* whole-rock samples to be of the VE-II type, as opposed to previously analysed Surtsey lavas which were all VE-I, and found them to be more evolved than previously reported. This allowed the authors to expand upon the concept that Surtsey erupted progressively more primitive magma during the evacuation of a vertically zoned magma chamber. Other chemical data from Surtsey rocks has included major-element analyses of starting materials used for experimental work on the evolution of alkali basalts^22^,^30^, and early Sr-isotope and REE analyses of Vestmannaeyjar lavas^32^.

**Figure 3:**
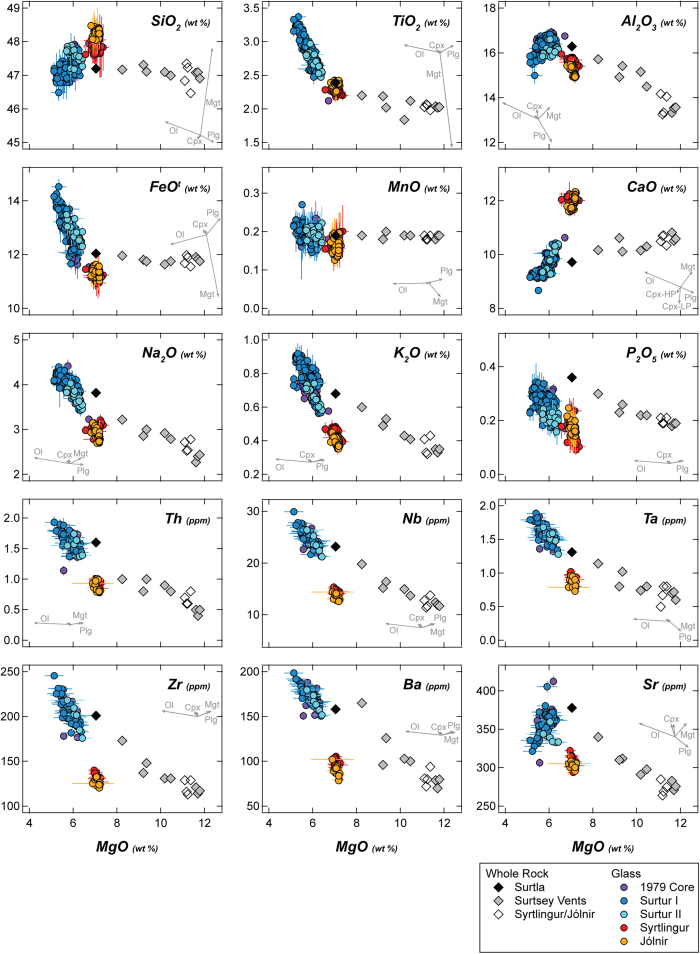
Major and selected trace-element variation diagrams versus MgO. Black arrow shows fractionation trend (~26% Olivine (Ol) + 21% Plagioclase (Plag) + 47% clinopyroxene (Cpx) + 6% magnetite (Mgt); calculated mineral compositions) modelled by Furman *et al*.^27^. Grey arrows show direction and magnitude for 5% fractionation of different phases. Major elements modelled using measured Surtsey olivine, plagioclase (Table S1), and oxide^26^ compositions, and compositions of clinopyroxene formed in high- (HP) and low pressure (LP) experiments^22^. Note that for all but CaO, the distinction between HP and LP Cpx is trivial. Trace elements modelled using standard distribution coefficients compiled in Rollinson^36^.

The petrogenetic study of Vestmannaeyjar lavas by Furman *et al*.^27^ (based on a PhD thesis^33^) had the same aims as the analysis of the Surtsey Magma Series presented here. Furman and colleagues examined the same Surtsey samples for which we present major-element chemistry (Table 1), measuring trace elements and Sr- Nd- and Pb-isotopes on a subset of the lavas. They extensively discussed major-element trends in their assessment of magma evolution, with graphical presentation of selected major-element oxides (see Figs 2, 3, 4 of Furman *et al*.^27^), and tabulation of the major-element chemistry of samples 9850 and 4638 (see Table 6 of Furman *et al*.^27^) – the primitive and evolved end-members used in their fractionation modelling. It is the primary objective of this paper to officially and completely present the full suite of Surtsey major-element data.

## Samples in the Surtsey magma and melt series

The samples used to define the Surtsey magma and melt series are housed at the Icelandic Museum of Natural History in Gardabaer, and are listed in Table 1.

Whole-rock major-element analyses are from a series of twelve scoria, bomb, and lava samples erupted from late December 1963 to May 19, 1967. All vents are represented in the whole-rock suite. Bombs and lava from *Surtur I* and *II* were collected on the island of Surtsey at various stages of activity. Scoria bombs from *Surtla* and *Syrtlingur* were dredged from their respective edifices, and were collected directly from the months-long ephemeral subaerial portion of *Jólnir*.

Major and trace-element analyses of sideromelane glasses in tephra collected on specific dates during the eruption cover pyroclastic activity from all vents, excluding *Surtla*. Collection of tephra from *Syrtlingur* and *Jólnir* was facilitated in part by their fallout deposition onto the main island of Surtsey^3^. The eruption dates for many of the tephras is known with high precision, some having been collected as they rained out directly from the eruptive plume onto the decks of various ocean-going vessels that were nearby or that were deliberately piloted into fallout zones (anecdotally against the better judgement of some of the skippers).

In 1979, a joint effort by the Icelandic Museum of Natural History and the Geothermal Research program of the US Geological Survey achieved the drilling of a 180.6 metre-deep borehole into Surtsey^15^,^34^. Core recovery on the borehole was very good, and the Surtsey magma series includes major-element analyses of sideromelane glass in tephras extracted from the core from 180.1 to 0.1 m depth. We interpret these glasses to represent the pre-emergent growth of the island of Surtsey in the lead up to the establishment of the emergent *Surtur I* vent.

## Analytical methods

The data presented here represent a combination of historical and new analyses from several laboratories.

Whole-rock major-element analysis (ref. 1 in Table 1) was by X-ray fluorescence (XRF) on fused glass beads at the Rock Geochemical Laboratory of the Geological Survey of Denmark and Greenland (Copenhagen). Fe_2_O_3_/FeO was determined by titration, and loss on ignition (LOI) was determined at 1000 °C^35^. Whole-rock trace-element analyses are given in Furman *et al*.^27^.

Glass major-element compositions by electron probe microanalysis (EPMA) are reported from several laboratories, and historic and new analyses are both presented where appropriate. Original analysis of drill core glasses (ref. 3 of Table 1) was with an ARL-EMX EPMA at the U.S. Geological Survey, Menlo Park, 1981, using 15 kV potential, 25 nA current and 10 μm rastered beam^19^. Original analysis of time series tephras (ref. 4 in Table 1) was with a JEOL 733 superprobe at the Massachusetts Institute of Technology, in 1987, using 15 kV, 10 nA, and 10 μm rastered beam. New analyses of drill core glasses (ref. 5 in Table 1) were with a Zeiss Sigma VP FEG SEM at the University of Otago, 2013, using 15 kV, 8 nA, and 10 μm beam. New analyses of time series tephras (ref. 6 of Table 1) were with a JEOL JXA-8230 superprobe at Victoria University of Wellington, in 2014, using 15 kV, 8 nA, and 10 μm beam.

Trace-element analysis of sideromelane glasses (ref. 7 of Table 1) was by laser ablation inductively coupled mass spectrometry (LA-ICPMS) with a Resonetics RESOlution M-50-LR 193 nm excimer laser ablation system coupled to an Agilent 7500 cs/ce Quadrupole ICP mass spectrometer at the University of Otago. Data were acquired with a spot size of 25–35 μm, ablating for 60 s at 5 Hz, using He as a carrier gas. Data was reduced offline using Si (from EPMA) and multiple replicate analyses of reference standards BHVO-2G and BCR-2G.

Modelling of major elements uses average olivine phenocryst and plagioclase microphenocryst compositions from Surtsey (supplementary Table S1); average clinopyroxene compositions from experimental phase equilibria studies^22^, and oxide (magnetite + ilmenite) compositions from SteinÞórsson^26^. Trace elements are modelled using standard basalt partition coefficients as compiled in Rollinson^36^, except where noted otherwise.

## The Surtsey magma and melt series

The petrography of the Surtsey Magma Series samples is consistent with the general descriptions for VE-I and VE-II types^13^,^28^. Mineral chemistry is given in Supplementary Table S1. Phenocryst compositions show cr-spinel ranging from Cr# 26–42 (average 32; Cr# = Cr/Cr + Al), olivine ranging from Fo_75_-Fo_87_ (average Fo_82_), and plagioclase ranging from An_53_-An_80_ (average An_70_). None show systematic zoning, and while olivine compositions do not vary systematically through time, plagioclase shows a very slight trend to more anorthite rich compositions. Except for two diopside crystals in one sample of *Jólnir* tephra (sample 9843), clinopyroxene is not observed as a phenocryst or microphenocryst phase in samples of the magma series, and is present only in the groundmass. Diopside is present along with olivine and plagioclase in rare, small (2–4 cm) gabbroic nodules found in Surtsey tephra and other lavas of Vestmannaeyjar^37^.

Major-element compositions of whole rocks (Table 2) varied dramatically and systematically during the course of the eruption (Fig. 2). The earliest-erupted whole-rock sample reported here, a scoria bomb from *Surtla* (4638), is the most evolved in the suite (MgO = 7.01 wt %, K_2_O = 0.68 wt %). Early activity from *Surtur I* did not yield appropriate samples for whole-rock analysis, but bomb and lava samples from the extended activity at *Surtur II* showed a marked evolution toward more primitive compositions (MgO = 8.22–10.17 wt %, K_2_O = 0.6–0.43 wt %) over the year from February 1964–1965. Bombs from both *Syrtlingur* and *Jólnir* in the period August 1965–1966 were consistently primitive (MgO = 11.44–11.19 wt %, K_2_O = 0.43–0.32 wt %). Once activity returned to the main island of Surtsey, lava from *Surtur I* and its satellite vents, from August 1966 to April 1967, showed a slight progression back to more evolved compositions (MgO = 11.69 − 9.17 wt %, K_2_O = 0.33 − 0.49 wt %), but the last lava sampled in May 1967 was then the most magnesian in the suite (MgO = 11.82 wt % K_2_O = 0.35 wt %). All samples in the magma series are nepheline normative, except for those from *Jólnir* and from *Surtur I* in 1966 (4693, 9850), which are hypersthene normative (tholeiitic in total alkali-silica space^27^).

**Figure 4:**
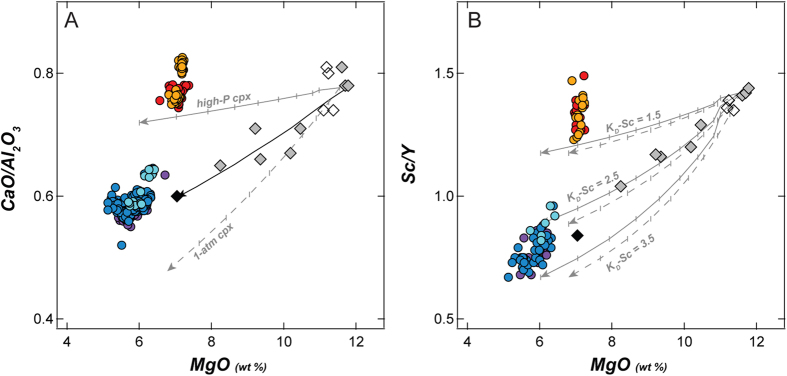
Role of clinopyroxene in crystal fractionation. Symbols as in Figs 2, 3. A) CaO/Al_2_O_3_ versus MgO. Black arrow shows major-element fractionation model of Furman *et al*.^27^, of ~48% fractionation of the approximate assemblage cpx (47%) > ol (27%) > plag (20%) > mgt (6%), with stepwise-calculated equilibrium mineral compositions. Grey arrows show (in 5% increments) fractionation of the same assemblage but with measured ol + plag (Supplementary Table S1), mgt^26^, and the high-P (>10 kbar, solid) and 1-atm (dashed) experimental cpx compositions from Thy^22^. B) Sc/Y versus MgO. Solid and dashed arrows calculated as in A, but showing influence from the range of possible *K*_*D*_*Sc* in cpx for alkali basalts^47^,^48^.

Temporal trends in tephra glass chemistry (Table 3) are similar to those in whole-rocks, in that they show a progression toward more primitive compositions over time. Glass from tephra in the 1979 drill core overlaps for all major and trace elements with those from *Surtur I+II*, and glasses from these tephras are chemically indistinguishable (Table 4; Figs 2,3). The earliest reliably time-stamped tephra samples collected after emergence are from pyroclastic activity at *Surtur I*, and are the most evolved in the suite, both in terms of major (MgO = 5.36 wt %, K_2_O = 0.86 wt %) and incompatible trace elements (Fig. 3). Tephra from *Surtur II* overlap with that from *Surtur I*, but with a trend toward less-evolved compositions. Glasses from *Syrtlingur* and *Jólnir* are noteworthy firstly for being nearly identical to each other in all major and trace elements (except for a slight tendency toward higher SiO_2_ and lower incompatible element concentrations at *Jólnir*; Figs 2, 3, 4), and secondly by being notably and ubiquitously less evolved, and more incompatible-element-depleted than Surtsey glasses (Figs 3,4).

Tephra glass is related to whole-rock analyses from the same time and/or vent, by olivine control, in keeping with the former representing melt and the latter representing melt + crystals (Fig. 3). Tephra glass on the main island of Surtsey, including at all levels in the 1979 drill core, shows some variability, but is bracketed by olivine control from the *Surtla* sample (4638, Dec. 1963) and the most-evolved and earliest-erupted whole-rock sample from *Surtur II* (9023, Feb. 1964). Some of the variability in Surtsey tephra glass can be related to minor plagioclase (Na_2_O, CaO, Sr) and Fe-Ti oxide (TiO_2_, FeO^t^) microphenocrysts, which are visible in the grains. Tephra glass from *Syrtlingur* and *Jólnir* shows no variation beyond analytical uncertainty, and similarly lies along olivine control from associated whole-rocks from the same vents. In this sense, the analysed glass from each vent’s tephra faithfully represents the melt fraction of the associated whole-rocks in the Surtsey Magma Series. The fact that glasses from *Syrtlingur* and *Jólnir* are dramatically different from those of Surtsey itself indicates that the more-primitive signature of whole-rocks from the satellite vents and later Surtsey whole-rocks is not simply the product of olivine accumulation.

## Discussion

### Was Surtsey’s magma a fractionation series, or a mixing series?

In their modelling of the Surtsey Magma Series, Furman *et al*.^27^ found that compositions could be related by up to ~48% fractionation of the assemblage: cpx (44–50%) > ol (26–27%), > plag (17–24%) > Ti-magnetite (6–7%), with stepwise-calculated mineral compositions and proportions. An issue noted as enigmatic by Furman *et al*.^27^ is that although clinopyroxene is the main phase in the modelled fractionation assemblage, it is not present as a phenocryst phase in Surtsey samples^13^, except in one sample of *Jólnir* tephra as noted above. A similar situation has been noted for lavas from Heimaey^38^,^39^, which is ~18 km NE of Surtsey, and is the site of the most recent eruption in the Vestmannaeyjar archipelago (Fig. 1A, inset)^37^. At both Surtsey and Heimaey, authors have embraced the “pyroxene paradox” e.g.^40^, relying on efficient, density-driven, clinopyroxene-melt separation at depth to explain the correlation between CaO/Al_2_O_3_ (and also Sc/Y^38^) versus MgO (Fig. 4).

Although the correlation between CaO/Al_2_O_3_ and Sc/Y versus MgO are strong in the Surtsey Magma Series, we note several issues with invoking clinopyroxene-dominated fractionation as the main control on variation among Surtsey’s magmas. First, although mechanisms for the efficient separation of clinopyroxene due to density contrast with the melt have been discussed by several authors^41^,^42^,^43^, we question whether such a mechanism can be 100% efficient, given the large amounts of clinopyroxene required^27^. Second, in 1 atm melting experiments on early Surtsey lavas^30^ olivine crystallized at 1220 °C, plagioclase at 1180 °C and clinopyroxene not until 1155 °C. We note that the temperatures of lavas at the surface of the *Surtur II* crater (e.g., exposed to the air and undoubtedly cooler than anywhere at depth) in March 1965 were still hot, 1151–1162 °C^44^, but density-driven separation of clinopyroxene would have required that it formed early, at high temperature. Third, there is a discrepancy in the trends that would be generated at high versus low pressure. Furman *et al*.^27^ inferred that evolution of the Surtsey magmas would have taken place at pressures >10 kbar (~30 km). Phase-equilibrium studies by Thy^22^, however, showed that subcalcic diopside crystallized in high-pressure runs (10–30 kbar) and that calcic diopside only crystallized in 1 atm experiments. For CaO/Al_2_O_3_ to vary strongly with MgO, only certain clinopyoxene compositions can be used in fractionation modelling (Fig. 4A). Furthermore, although Sc is moderately compatible in clinopyroxene, its partition coefficient (*K*_*D*_*Sc*) is dependent both on temperature and on composition e.g,^45^. Experiments^22^ showed clinopyroxene to be more magnesian at high pressure (En_40–58_%) than at 1 atm (En_36–41_%), which could mean a *K*_*D*_*Sc* lower by 1/2 or 2/3 for high-P clinopyroxene^46^. For alkali basalts such as those of Iceland’s EVZ, a range of 1.31–3.5 is probably realistic^47^,^48^, with lower *K*_*D*_*Sc* for higher-T. Thus, the fits of fractionation models to observed Sc/Y trends in Surtsey magma depend strongly on the choice of *K*_*D*_*Sc* (Fig. 4B).

**Figure 5:**
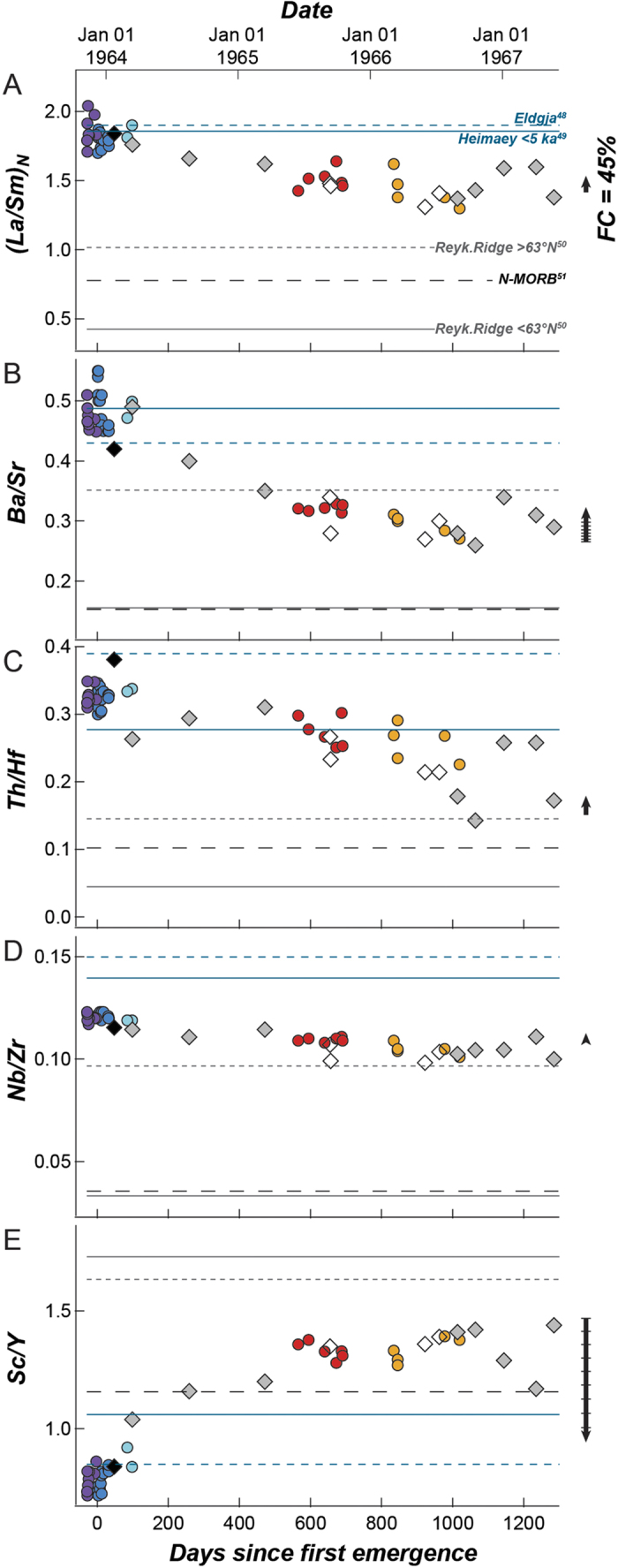
Time variation for various trace-element ratios, compared to possible Icelandic mixing end-members (La/Sm_N_ is chondrite-normalized^79^). Symbols as in Figs 2, 3, 4. Enriched alkalic end-members shown: (1) Eldgjá, EVZ^49^ (which has been identified as a component in lavas of the northern Reykjanes Ridge^50^); and (2) young (<5 ka) lavas from Heimaey, Vestmannaeyjar^38^. Depleted end-members shown: Reykjanes Ridge basalts from North and South of 63 °N^52^ and global average N-MORB^53^. Black arrows to right of each frame shows the effect of 45% (total length of arrow, marked in 5% increments where appropriate) fractionation of cpx-dominated assemblage described by Furman *et al*.^27^, using starting composition of sample 9850 and standard basalt partition coefficients as compiled by Rollinson^36^. Note that in all cases, trends are from more Icelandic alkalic basalt end members toward more depleted ridge-like end members over time, and that with the exception of Sc/Y, fractionation cannot explain the degree of variation in trace-element ratios. Degree of mixing is not equivalent for all ratios, suggesting that mixing is heterogeneous, and some ratios (e.g., Nb/Zr) are not well represented by lavas from Eldgjá or Heimaey, suggesting that the off-axis location of Surtsey involves as-yet unconstrained, end members.

The ambiguities outlined above are not in themselves sufficient to discard cpx fractionation as an important control on relationships among samples of the Surtsey Magma Series. However, trends in some trace-element ratios cannot be explained solely by fractionation. In particular, incompatible trace-element ratios such as chondrite-normalized La/Sm_N_, Th/Hf, Ba/Sr (Figs 5A–C), K/Rb and Zr/Y (not shown), and to a lesser extent Nb/Zr (Fig. 5D), show systematic variability through time to a degree that cannot have been produced by crystal fractionation of a clinopyroxene-dominated assemblage (scaled arrows, Fig. 5).

We suggest that trends in trace-element ratios can be better explained by magma mixing. End members for this inferred mixing are, however, only approximated by published compositions. The enriched component is an alkalic basalt similar to lavas from the EVZ at Eldgjá^49^, which have been used as a proxy for the main basaltic component on Iceland in investigations of ridge-plume interaction along the Reykjanes Ridge^50^, and/or those from the Vestmannaeyjar at Heimaey^38^. Lava from *Surtla* (4638) is compositionally matched in many trace elements to lavas from Eldgjá, the earliest lavas from Surtur I are better matched to the young (<5 ka) eruptives on Heimaey that form a rift-parallel array^51^. An important note, however, is that all Surtsey eruptives have significantly lower Nb/Zr than alkalic lavas of the EVZ and Vestmannaeyjar (both for whole rocks analysed by Furman *et al*.^27^ and for glasses analysed in this study), indicating that they may include an as-yet unidentified component. The depleted component is a ridge-like basalt broadly similar to the tholeiites of the submarine Reykjanes Ridge^50^,^52^, which compositionally approximates global N-MORB^53^ on average, but varies with latitude as the northern segment interacts with Iceland^14^,^50^,^52^. Figure 5 shows temporal trends for several incompatible trace-element ratios of the Surtsey Magma Series, compared with the aforementioned end-members. Although all trace-element ratios trend from more EVZ-like *Surtla* and Heimaey-like early Surtsey to more ridge-like *Syrtlingur, Jólnir*, and late-Surtsey, the ratios of mixing between the plotted end-members are not consistent (Fig. 5). This is probably because end-member magmas in the off-axis propagating tip of the EVZ remain poorly identified, not having been studied as extensively as magmas of the on-axis Reykjanes Ridge-Iceland Plume region^14^,^32^,^49^,^50^,^52^,^54^,^55^,^56^,^57^,^58^,^59^,^60^,^61^. We note, additionally, that although temporal trends in Sc/Y could be explained by crystal fractionation, they can be equally well explained by mixing (Fig. 5E).

Isotopic ratios are often good indicators of magma mixing because they are unaffected by crystal fractionation. Surtsey lavas analysed by Furman *et al*.^27^,^62^ have ^87^Sr/^86^Sr, ^143^Nd/^144^Nd, and Pb isotopic ratios that are intermediate to those of the Reykjanes Ridge^50^,^52^,^59^,^63^,^64^,^65^ and Iceland’s EVZ/Vestmannaeyjar^27^,^32^,^56^,^63^,^66^ (not shown), which generally supports a mixing hypothesis. However, because there is significant overlap in the isotopic signatures of these end members, and because isotopic ratios of Surtsey lavas did not vary (beyond analytical uncertainty) during the course of the eruption^27^, isotopic ratios are non-discriminating of magma mixing processes at Surtsey.

Magma mixing as an alternative to crystal fractionation at Surtsey is a simple but attractive hypothesis for several reasons. In addition to explaining much of the variability in compositions listed above, it also explains major-element trends. Specifically, although Vestmannaeyjar is the SW extension of the alkali-olivine basalt region of Iceland^13^,^67^, only *Surtla* whole-rock samples plot unambiguously above the alkalic-tholeiitic divide in total alkali-silica space (not shown). The rest of the samples show a temporal trend through transitional space toward tholeiitic compositions. Petrographically, mixing does not require the crystallization of large amounts of clinopyroxene, which is absent except as a groundmass phase and in rare xenoliths; we consider it preferable to avoid drawing upon unseen processes, particularly when doing so results in a paradox. Physically, the injection of new, hot, primitive magma into a pre-existing body of more evolved magma is a well-known mechanism for triggering an eruption e.g,^68^ and for sustaining that eruption so long as the supply of new magma persists.

Perhaps the most important argument against fractionation of clinopyroxene in the Surtsey Magma Series involves the small size of the edifice, and the short duration of the eruption. The pyroxene paradox determined for MORB glasses has been defined over long ridge segments, on dredged materials, for which eruptive relationships cannot be accurately known^40^,^41^,^42^,^43^,^69^. In the Vestmannaeyjar, the lavas of Heimaey that are apparently related by clinopyroxene-dominated fractional crystallization are from ten volcanic centres erupted over tens of thousands of years throughout the Holocene, and into the Upper Pleistocene^38^,^39^,^51^. Surtsey’s eruption was an essentially continuous event lasting less than four years. Invocation of disparate crystallization histories for pulses of magma erupted only weeks apart from vents separated by only 100’s of metres indicates a physically intractable conception of rates and scales of fractionation. In contrast, successive injections of ridge-like magma into more shallowly stored alkalic magma (and other minor components) might be expected to continue through the brief duration of a single eruption.

### Regional perspectives on magma mixing at Surtsey

Magma mixing has been established as an important process where the MAR and a mantle plume interact in the Iceland region. Recognition of northward increasing chondrite-normalized La/Sm_N_ by Schilling^14^,^54^ was instrumental in drawing attention to increased ridge-plume interaction along the Reykjanes Ridge toward Iceland. Following this key finding, models of ridge-plume interaction, mantle heterogeneity, and geochemical structure of the Iceland plume have been greatly refined through intensive petrological work^14^,^32^,^49^,^50^,^52^,^54^,^55^,^56^,^57^,^58^,^59^,^60^,^61^,^65^. That the end-members for our mixing hypothesis at Surtsey are not rigorously matched by compositions from these studies is not surprising given the complexity of Iceland’s magmatic systems. Murton *et al*.^50^, for example, identified up to six mixing end members along the Reykjanes Ridge, and these interact with the Iceland plume that is itself heterogeneous^66^,^70^.

Evolution of propagating rifts involves magma that resides in small ephemeral magma chambers becoming progressively more connected as the rift propagates and magma supply increases, until full connectivity is achieved^71^,^72^. This model has been applied to explain the petrology of lavas at Heimaey^38^, where magma bodies that are still relatively isolated are envisaged to be undergoing polybaric fractionation. We suggest that Surtsey, which is further along the SW propagator than is Heimaey, represents an even more embryonic manifestation of magmatic diversity at a propagating tip. Although the more evolved lavas of Surtsey are geochemically similar to those from Heimaey, there are significant differences in some trace elements, including an offset to lower Nb/Zr (Fig. 5D), that suggest limited communication between the magma bodies of Surtsey and those of the Vestmannaeyjaar as a whole^73^. Furthermore, the irregularity of mixing proportions from one Surtsey vent to another (e.g., Th/Hf in Fig. 5C), suggests limited physical communication among the vents’ respective feeder systems.

Finally, the appearance of small proportions of a ridge-like mixing end member at Surtsey is consistent with the current tectonic environment of SW Iceland. The WVZ is now receding, the EVZ propagating, and the South Iceland Seismic Zone is accommodating an active ridge jump from the WVZ to the EVZ by transverse faulting^74^,^75^. This may even lead to the EVZ linking up with the submarine Reykjanes Ridge^38^ as it replaces the WVZ as the main rift in Southern Iceland.

### Local perspectives on magma mixing at Surtsey

The Surtsey Magma Series documents a temporal progression from more evolved to more primitive compositions. This could suggest evacuation of a vertically zoned magma chamber(s)^31^, but does not make explicit the origin of this zonation. We suggest that the injection of a primitive, ridge-like component into pre-existing, poorly connected volumes of broadly EVZ/Vestmannaeyjar-like alkali basaltic magma caused overpressure to develop in the stored magma system, and caused the eruption that produced Surtsey and its siblings.

Early eruptive products from *Surtla* (and to a lesser extent *Surtur I+II*) are the most EVZ-like in the suite (although lower in Nb/Zr), suggesting that they partially represent magma delivered by along-rift transport from the more established EVZ to the NE. However, although *Surtla* erupted the most evolved magma in the suite, but with 7.01 wt% MgO it is still relatively primitive, as are early lavas on Surtsey. This indicates a relatively short residence time of this magma in shallow storage below what would become the site of Surtsey Volcano - short enough to preclude significant closed-system crystallization and/or differentiation. The regional along-rift disturbance and deformation that would have accompanied early injection of EVZ-like magma into the Surtsey region was probably instrumental in initiating dike propagation from the ridge-like source along axis, into the same area. Injection of this depleted, ridge-like magma caused it to partially mix with ponded EVZ-like magma under Surtsey itself, and this is inferred to have been the most volumetrically significant stored sub-volume. The fact that *Syrtlingur* and *Jólnir* saw the eruption of very primitive magma probably indicates that the pre-eruption reservoirs of EVZ-type magma were minimal in these areas. The eruption of similarly primitive lava once activity returned to Surtsey, marked the impending exhaustion of all of the original EVZ-type magma.

Feeder dikes are common on the island of Surtsey, and thin dikes are present at two intervals in the 1979 drill core^15^. Also, gravity surveys on Surtsey have shown separate planar nonfragmental basaltic bodies (e.g, dikes) feeding the *Surtur* vents^76^. Injection of ridge-like magma into the subsurface at Surtsey would also have produced sub-vertical dikes, given the rift setting and fissure-like beginnings of the eruption^1^,^2^,^5^,^15^. If these were of similar small size as the feeder dikes (10 s of centimetres thickness), with short residence time available for mixing during an active eruption, it is not surprising that mixing should vary through the different times and vents of the Surtsey eruption.

## Conclusions

The 1963–67 eruption of Surtsey (Iceland) is the canonical example of shallow-to-emergent phreatomagmatic eruptions. Previously unpublished and new analyses of whole-rocks and sideromelane glass in tephra from time- and space-located samples throughout Surtsey’s eruption respectively define a Surtsey Magma Series and Surtsey Melt Series. Progressive changes in the geochemistry of eruptive products can be explained by mixing between ephemeral and poorly connected volumes of broadly EVZ-like alkalic basalt typical of Iceland’s Vestmannaeyjar, with primitive ridge-like basalt formed at the propagating ridge tip. Surtsey not only demonstrated the dynamics of magma-water interaction in subaqueous eruptions, but also exemplifies processes occurring at the extreme propagating tip of an off-axis ridge in the vicinity of the Iceland plume.

## Additional Information

**How to cite this article**: Schipper, C.I. *et al*. The Surtsey Magma Series. *Sci. Rep*. **5**, 11498; doi: 10.1038/srep11498 (2015).

## Supplementary Material

Supplementary Information

## Figures and Tables

**Table 1:** Samples.

No.	Type	Analysis		Erupted Date	Collected by	Notes
		WR	Glass			
***Surtla***						
4638	Scoria	1,2		28 Dec '63 - 6 Jan '64	K Thors	Dredge (H74-D13-1); 74 mbsl; 10 Aug '74
***Surtur I***						
Various depths		3,5,6,7	~8-14 Nov '63	SP Jakobsson	Sideromelane in tephra present in the 1979 Surtsey drill core.	
9809	Tephra		6,7	15 Nov '63	S Thorarinsson	Collected from fallout onboard v/s Albert
9810	Tephra		6,7	15 Nov '63	S Thorarinsson	Collected from fallout onboard v/s Albert
11236	Tephra		6,7	15 Nov '63	T Einarsson	Collected from unspecified vessel
9811	Tephra		4,6,7	16 Nov '63	S Thorarinsson	Collected during fallout onboard fishing vessel Haraldur
801	Tephra		6,7	17 Nov '63	G Kjartansson	Collected from fallout onboard v/s Odni, 3 km from Surtsey
9812	Tephra		6,7	21 Nov '63	Crew of v/s Ódinn	Collected from fallout onboard v/s Ódinn
9813	Tephra		6,7	23 Nov '63	S Thorarinsson	Collected from fallout onboard v/s Thor
9814	Tephra		4,6,7	25 Nov '63	S Thorarinsson	Collected during fallout onboard a fishing vessel
9815	Tephra		6,7	25 Nov '63	S Thorarinsson	Collected from fallout onboard v/s Albert
9817	Tephra		6,7	26 Nov '63	S Thorarinsson	Collected from fallout onboard v/s Thor
9816	Tephra		6,7	1 Dec '63	S Thorarinsson	Collected from fallout onboard v/s Ódinn at 19:00
11218	Tephra		6,7	14 Dec '63	S Thorarinsson	Collected from fallout onboard v/s Albert
9820	Tephra		6,7	Mid Dec '63	GE Sigvaldsson	Collected on NE Surtsey
11239	Tephra		6,7	16 Dec '63	Th Sigurgeirsson	Collected on Surtsey
4693	Lava	1,2		19-27 Aug '66	SP Jakobsson	Collected at NE coast of Surtsey; 28 Aug '66
9850	Lava	1,2		13 Oct '66	S Thorarinsson	Collected from E coast of Surtsey
9853	Lava	1,2		late Mar '67	S Thorarinsson	Collected from Surtur I
9854	Lava	1,2		~19 May '67	S Thorarinsson	Collected from Surtur I
***Surtur II***						
9822	Tephra		4,6,7	1-10 Feb '64	SP Jakobsson	From outer, W slope of Surtsey
9823	Bomb	1,2		14-28 Feb '64	SP Jakobsson	Collected from bomb fragment-rich layer; 15 Aug '64
9821	Tephra		6,7	19 Feb '64	Crew of m/s Haraldur	Collected during fallout onboard fishing vessel Haraldur
9826	Lava	1,2		Jul-Aug '64	SP Jakobsson	Collected from S of Surtur II vent; 15 Aug '64
9828	Lava	1,2		25-26 Feb '65	S Thorarinsson	Collected 27 Feb '65 from Surtur II
***Syrtlingur***						
9832	Tephra		4,6,7	May-June '65	?	Syrtlingur tephra collected at research hut on Surtsey
9833	Tephra		6,7	Jun-Jul '65	?	Syrtlingur tephra collected atop Surtur II lavas
11221	Tephra		6,7	Aug '65	S Thorarinsson	Syrtlingur tephra collected on Syrtlingur
8066	Bomb	1,2		Autumn '65	K.Thors, SP Jakobsson	Dredge (A82-D1); 30-40 mbsl; 4 Nov '82
8067	Bomb	1,2		Autumn '65	K.Thors, SP Jakobsson	Dredge (A82-D2); 60-80 mbsl; 4 Nov '82
9834	Tephra		6,7	16 Sept '65	S Thorarinsson	Syrtlingur tephra collected on W Surtsey
9831	Tephra		6,7	Sept-Oct '65	S Thorarinsson	Syrtlingur tephra collected on Surtsey
9835	Tephra		4,6,7	4 Oct '65	S Thorarinsson	Syrtlingur tephra collected at northern spit of Surtsey
***Jólnir***						
11222	Tephra		6,7	25 Feb '66	Crew of v/s Maria Júlia	Fallout onboard v/s Maria Júlia
9836	Tephra		4,6,7	8 Mar '66	Crew of v/s Maria Júlia	Collected from Jólnir during fallout
9837	Tephra		6,7	Mar '66	Crew of v/s Maria Júlia	Collected from Jólnir during fallout
9839	Bomb	1,2		24 May '66	S Thorarinsson	Surface sample collected on Jólnir
9842	Bomb	1,2		3 Jul '66	S Thorarinsson	Surface sample collected on Jólnir
9843	Tephra		4,6,7	18 Jul '66	SP Jakobsson	Collected during fallout on S shore of Surtsey
9846	Tephra		6,7	29 Aug '66	SP Jakobsson	Jólnir tephra collected on N Surtsey
***Satellite Vents***						
9851	Lava	1,2		1-3 Jan '67	SP Jakobsson	From vent on inner N wall of Surtur I

^1^Whole rock major element analysis by XRF at Greenland Geological Society, Copenhagen.

^2^Whole rock trace element analysis given in Furman *et al*. (1991).

^3^Glass major elements by EPMA at USGS Menlo Park, 1981, Jakobsson and Thorseth (in prep.).

^4^Glass major elements by EPMA at Massachussets Institute of Technology, 1987.

^5^Glass major elements by EPMA at U. of Otago, 2013.

^6^Glass major elements by EPMA at Victoria University of Wellington, 2014.

^7^Glass trace elements by LA-ICMPA at U. of Otago, 2014.

**Table 2:** The Surtsey Magma Series (Whole Rock).

No.	4638	9823	9826	9828	8066	8067	9839	9842	4693	9850	9851	9853	9854
**Date**	Late Dec 1963	Feb 14-28 1964	Jul-Aug 1964	Feb 25-26 1965	Autumn 1965	Autumn 1965	May 24 1966	Summer 1966	Aug 19-27 1966	Oct 13 1966	Jan 1-3 1967	Mar 31 1967	May 19 1967
**Day**	47	98	258	471	655	656	922	962	1013	1064	1144	1235	1285
**Vent**	Surtla	Surtur II	Surtur II	Surtur II	Syrtlingur	Syrtlingur	Jólnir	Jólnir	Surtur I	Surtur I	Surtur I Satellite Vent	Surtur I	Surtur I
**Form**	scoria	bomb	lava	lava	bomb	bomb	bomb	bomb	lava	lava	lava	lava	lava
**SiO**_**2**_	46.99	47.07	47.04	47.03	46.75	46.84	47.25	47.08	47.01	47.04	47.06	47.12	47.06
**TiO**_**2**_	2.38	2.20	2.02	1.84	1.99	2.03	2.07	2.05	2.04	2.02	2.12	2.18	2.04
**Al**_**2**_**O**_**3**_	16.22	15.69	15.40	15.14	14.13	14.17	13.22	13.30	13.31	13.53	14.52	14.86	13.61
**Fe**_**2**_**O**_**3**_	2.34	2.05	2.09	1.35	1.76	1.40	2.10	2.63	2.11	1.59	2.29	1.65	1.76
**FeO**	9.90	10.11	9.87	10.42	10.12	10.44	10.09	9.52	10.01	10.40	9.74	10.29	10.23
**MnO**	0.19	0.19	0.20	0.19	0.19	0.19	0.18	0.18	0.18	0.19	0.19	0.18	0.19
**MgO**	7.01	8.22	9.34	10.17	11.44	11.10	11.16	11.19	11.59	11.69	10.48	9.17	11.82
**CaO**	9.68	10.15	10.10	10.15	10.40	10.50	10.68	10.69	10.80	10.51	10.33	10.58	10.62
**Na**_**2**_**O**	3.80	3.21	3.00	2.92	2.80	2.72	2.52	2.53	2.27	2.40	2.79	2.85	2.45
**K**_**2**_**O**	0.68	0.60	0.53	0.43	0.43	0.41	0.33	0.32	0.35	0.33	0.41	0.49	0.35
**P**_**2**_**O**_**5**_	0.36	0.30	0.26	0.22	0.21	0.21	0.19	0.19	0.18	0.19	0.22	0.23	0.19
**LOI**	0.25	0.13	0.19	0.12	0.38	0.30	0.19	0.38	0.11	0.11	0.13	0.10	0.21
**Total**	99.80	99.92	100.04	99.98	100.60	100.31	99.98	100.06	99.96	100.00	100.28	99.70	100.53

Whole rock major element analysis by XRF at Greenland Geological Society, Copenhagen.

**Table 3:** The Surtsey Melt Series (Sideromelane Glass).

No.	9809	9810	11236	9811*	801	9812	9813	9814*	9815	9817	9816	11218	9820	11239	9822*	9821	9832*	9833	11221	9834	9831	9835*	11222	9836*	9837	9843*	9846
**Date**	15-Nov-63	15-Nov-63	15-Nov-63	16-Nov-63	17-Nov-63	21-Nov-63	23-Nov-63	25-Nov-63	25-Nov-63	26-Nov-63	1-Dec-63	14-Dec-63	mid-dec-63	16-Dec-63	5 Feb '64	19-Feb-64	May-Jun '65	Jun-Jul-65	Aug '65	16-Sep-65	Sept-Oct '65	4 Oct '65	25-Feb-66	8-Mar-66	8-Mar-66	18 Jul '66	29-Aug-66
**Day**	1	1	1	3	3	7	9	11	11	12	17	30	31	32	84	97	565	594	640	672	687	690	834	845	845	977	1019
**Vent**	Surtur I	Surtur I	Surtur I	Surtur I	Surtur I	Surtur I	Surtur I	Surtur I	Surtur I	Surtur I	Surtur I	Surtur I	Surtur I	Surtur I	Surtur II	Surtur II	Syrtlingur	Syrtlingur	Syrtlingur	Syrtlingur	Syrtlingur	Syrtlingur	Jólnir	Jólnir	Jólnir	Jólnir	Jólnir
***EPMA (wt%)***																											
**SiO**_**2**_	46.18	46.31	46.38	46.44	46.40	46.50	46.46	46.57	46.53	46.41	46.61	46.66	46.99	46.76	46.99	47.11	47.48	47.30	47.24	47.39	47.36	47.30	47.86	47.88	47.81	47.69	47.88
**TiO**_**2**_	3.07	3.00	3.02	3.02	2.95	2.92	2.91	2.75	2.80	2.80	2.66	2.56	2.55	2.63	2.54	2.76	2.24	2.21	2.25	2.28	2.23	2.30	2.36	2.35	2.35	2.36	2.36
**Al**_**2**_**O**_**3**_	15.68	15.88	15.92	15.94	16.06	15.90	15.94	16.17	15.98	16.09	16.16	16.28	16.16	16.47	15.92	15.87	15.54	15.33	15.57	15.57	15.49	15.45	15.34	15.26	15.23	14.81	14.84
**FeO**^**t**^	13.42	13.19	13.27	13.21	12.97	13.08	12.84	12.71	12.70	12.64	12.41	11.90	12.39	12.48	12.42	12.94	11.19	11.10	11.17	11.20	11.02	11.16	11.34	11.28	11.13	11.20	11.27
**MnO**	0.20	0.19	0.20	0.20	0.19	0.20	0.19	0.20	0.19	0.19	0.18	0.17	0.18	0.18	0.19	0.20	0.16	0.17	0.17	0.17	0.16	0.16	0.16	0.16	0.16	0.17	0.16
**MgO**	5.36	5.44	5.39	5.50	5.48	5.71	5.66	5.92	5.67	5.73	6.05	5.83	6.12	5.99	6.25	5.82	7.03	7.09	6.88	6.96	6.95	6.90	6.92	6.98	6.93	7.10	7.11
**CaO**	9.14	9.20	9.20	9.16	9.15	9.20	9.31	9.41	9.43	9.41	9.67	9.83	10.02	9.60	10.17	9.36	11.93	11.96	11.83	11.77	11.93	11.73	11.58	11.58	11.63	12.11	12.00
**Na**_**2**_**O**	4.13	4.05	4.04	4.13	4.06	4.04	4.09	3.92	3.88	3.92	3.86	3.77	3.58	3.72	3.54	3.82	2.90	3.07	2.93	3.00	2.87	3.01	3.00	2.92	2.93	2.77	2.78
**K**_**2**_**O**	0.86	0.83	0.83	0.83	0.82	0.81	0.81	0.75	0.78	0.77	0.74	0.74	0.61	0.64	0.59	0.70	0.44	0.41	0.45	0.46	0.43	0.45	0.42	0.41	0.41	0.38	0.36
**Cr**_**2**_**O**_**3**_	0.01	0.02	0.01	0.01	0.01	0.01	0.01	0.01	0.02	0.02	0.01	0.02	0.02	0.01	0.02	0.02	0.02	0.02	0.03	0.03	0.02	0.03	0.04	0.02	0.03	0.03	0.03
**P**_**2**_**O**_**5**_	0.29	0.28	0.28	0.28	0.28	0.28	0.28	0.25	0.26	0.27	0.26	0.26	0.20	0.20	0.20	0.21	0.18	0.12	0.17	0.19	0.19	0.18	0.19	0.18	0.18	0.16	0.17
**SO**_**3**_	0.13	0.14	0.13	0.12	0.13	0.12	0.12	0.12	0.12	0.11	0.12	0.12	0.11	0.12	0.11	0.10	0.13	0.10	0.13	0.12	0.13	0.13	0.12	0.12	0.12	0.13	0.13
**Total**	98.46	98.53	98.67	98.84	98.50	98.77	98.63	98.77	98.33	98.36	98.73	98.14	98.91	98.82	98.97	98.90	99.23	98.88	98.81	99.13	98.80	98.82	99.33	99.14	98.90	98.91	99.07
**n (spots/grains)**	30/10	30/10	29/10	30/10	30/10	30/10	27/9	30/10	30/10	29/10	30/10	30/10	30/10	30/10	26/9	26/9	30/10	25/9	30/10	26/9	29/10	29/10	18/7	30/10	30/10	27/9	27/9
***LA-ICPMS (ppm)***																											
**Sc**	29.04	27.29	28.75	29.42	26.65	27.65	27.25	27.48	28.50	28.00	28.19	28.35	28.28	29.00	30.72	32.57	36.36	35.71	35.54	35.73	35.92	37.37	34.52	35.61	35.71	36.83	37.40
**V**	301.08	292.72	302.33	299.09	281.90	277.56	271.36	266.23	268.16	285.08	269.85	261.13	267.68	276.92	276.00	293.39	318.97	318.60	315.64	313.23	321.83	325.71	335.33	322.78	321.53	334.71	335.36
**Co**	41.93	41.66	41.97	41.30	42.09	41.92	41.22	42.60	41.35	42.09	43.00	43.10	41.25	40.86	41.12	43.67	46.43	45.84	46.16	45.22	45.99	46.03	43.55	45.57	44.80	47.32	46.47
**Ni**	43.28	48.55	44.22	43.94	52.61	56.48	56.58	60.52	58.15	50.40	71.34	65.74	67.09	56.33	57.97	60.44	97.71	100.81	98.86	97.30	98.13	95.13	93.19	97.17	98.72	101.17	100.22
**Cu**	59.52	60.21	63.39	62.15	48.50	59.04	56.85	58.07	57.24	62.87	59.48	58.95	58.52	64.23	62.00	72.77	97.68	94.41	90.61	94.70	116.02	98.93	109.26	93.98	85.07	99.97	96.08
**Zn**	108.24	103.69	104.46	101.78	101.36	95.90	96.90	95.10	92.86	98.71	107.19	86.42	88.59	84.44	97.16	93.87	108.83	83.38	79.93	82.53	84.89	86.57	84.53	85.77	82.08	85.41	84.36
**Ga**	24.51	24.75	24.28	24.85	24.20	23.83	24.24	24.08	23.47	24.08	22.45	22.67	23.10	22.35	23.33	23.11	21.48	21.53	21.30	21.60	22.12	21.51	20.25	21.91	21.84	22.22	21.70
**Rb**	15.93	16.18	15.98	15.92	15.91	14.57	15.37	15.25	13.94	15.55	14.18	14.02	13.95	14.11	15.39	14.34	7.95	8.14	8.11	7.79	8.43	7.97	7.20	7.25	7.25	6.73	6.29
**Sr**	338.66	347.15	338.39	335.51	358.03	352.10	364.04	361.05	375.70	351.34	362.10	372.37	367.30	357.13	347.69	333.51	305.37	306.01	301.05	306.12	314.41	316.92	304.86	302.92	303.74	301.05	298.37
**Y**	38.80	37.89	38.77	38.77	37.17	37.09	35.46	34.26	34.86	38.66	34.80	34.01	34.55	34.29	36.63	35.35	26.41	26.86	26.14	26.90	28.05	28.53	25.92	27.52	28.12	26.45	27.16
**Zr**	217.61	210.02	218.90	219.71	207.11	208.13	202.53	195.22	199.22	213.27	191.76	193.62	199.25	191.35	209.99	187.46	129.80	130.00	127.96	131.47	136.37	137.83	127.33	129.57	131.92	122.33	124.95
**Nb**	26.38	25.33	26.37	26.48	25.22	25.53	24.75	23.63	23.77	26.32	23.59	23.42	23.97	23.00	25.09	22.36	14.23	14.37	14.00	14.19	14.96	15.01	13.89	13.49	13.90	12.81	12.67
**Ba**	181.20	178.75	185.35	182.92	177.85	177.12	172.32	169.85	172.85	177.72	163.94	168.03	166.07	165.65	173.34	157.44	96.88	96.18	96.62	98.44	103.51	103.60	94.76	90.87	92.34	85.62	80.75
**La**	20.48	19.46	20.53	20.40	19.40	19.83	19.10	18.26	18.62	19.91	17.58	18.15	18.16	17.79	19.83	17.45	11.41	11.27	11.16	11.36	11.80	11.77	11.43	10.75	10.91	10.03	9.81
**Ce**	48.78	47.53	49.00	49.93	46.27	47.27	45.92	45.20	44.23	47.59	42.24	43.61	44.18	43.03	46.83	42.00	28.17	28.08	27.92	28.31	30.09	29.49	27.86	26.86	26.95	25.54	25.15
**Pr**	6.56	6.22	6.66	6.57	6.24	6.14	6.36	5.68	6.08	6.47	5.85	5.83	5.92	5.61	6.42	5.54	3.78	3.77	3.84	3.92	4.10	3.95	3.97	3.80	3.70	3.60	3.53
**Nd**	29.93	28.27	30.00	30.28	28.46	27.76	27.89	27.63	28.36	29.42	26.17	26.69	27.12	25.95	28.04	25.13	18.29	18.20	17.74	18.72	19.59	19.25	18.79	18.16	18.33	17.59	17.58
**Sm**	7.16	6.90	7.81	7.28	6.70	6.91	7.08	6.87	6.67	7.07	6.33	6.43	6.69	6.41	6.74	6.21	4.86	4.90	5.05	4.79	4.64	5.19	4.55	5.03	4.78	4.70	4.87
**Eu**	2.35	2.33	2.35	2.47	2.26	2.34	2.31	2.25	2.24	2.33	2.21	2.22	2.24	2.08	2.22	2.16	1.65	1.75	1.56	1.67	1.85	1.80	1.70	1.71	1.74	1.75	1.74
**Gd**	7.56	7.19	7.11	7.91	7.39	7.63	7.31	7.27	7.42	7.81	6.97	7.05	6.79	6.97	6.99	7.10	5.25	5.18	5.41	5.14	5.63	6.01	5.16	5.62	5.23	5.22	5.60
**Tb**	1.16	1.11	1.19	1.28	1.13	1.19	1.15	1.04	1.10	1.17	1.03	0.99	0.98	1.02	1.16	1.02	0.79	0.81	0.76	0.81	0.85	0.88	0.77	0.92	0.88	0.87	0.90
**Dy**	7.32	6.73	7.75	7.55	7.04	7.26	7.02	6.79	6.52	7.33	6.57	6.86	6.68	6.67	7.40	6.65	5.43	5.21	5.19	5.23	5.61	5.73	5.16	5.61	5.56	5.26	5.43
**Ho**	1.40	1.33	1.51	1.52	1.38	1.39	1.32	1.24	1.29	1.38	1.30	1.32	1.27	1.28	1.38	1.22	0.98	0.97	1.00	0.97	1.06	1.08	1.06	1.06	0.99	1.02	1.04
**Er**	3.99	3.62	4.19	4.07	4.00	3.87	3.71	3.66	3.74	4.06	3.56	3.75	3.64	3.75	3.84	3.62	2.92	2.91	2.59	2.89	2.92	3.02	2.62	2.81	2.89	2.62	2.70
**Tm**	0.57	0.54	0.62	0.60	0.53	0.57	0.56	0.54	0.48	0.57	0.52	0.52	0.52	0.53	0.48	0.51	0.39	0.39	0.36	0.39	0.39	0.40	0.35	0.42	0.40	0.35	0.41
**Yb**	3.59	3.54	3.55	3.65	3.65	3.35	3.42	3.31	3.38	3.56	3.20	3.15	3.16	3.22	3.36	3.03	2.42	2.42	2.40	2.49	2.62	2.64	2.32	2.25	2.37	2.36	2.30
**Lu**	0.53	0.49	0.54	0.55	0.48	0.51	0.52	0.43	0.52	0.52	0.48	0.47	0.44	0.45	0.49	0.49	0.34	0.38	0.35	0.33	0.37	0.35	0.32	0.34	0.35	0.31	0.35
**Hf**	5.15	4.93	5.78	5.24	5.28	5.11	5.06	4.92	4.69	5.23	4.64	4.63	4.85	4.71	4.78	4.38	3.29	3.25	3.21	3.44	3.80	3.55	3.33	3.35	3.66	3.04	3.54
**Ta**	1.63	1.64	1.74	1.74	1.62	1.63	1.60	1.61	1.57	1.61	1.52	1.51	1.48	1.43	1.59	1.45	0.88	0.89	0.91	0.93	0.98	0.95	0.88	0.88	0.87	0.83	0.81
**Pb**	1.81	2.00	2.35	1.88	1.86	1.73	1.79	1.86	1.67	1.66	1.62	1.65	1.75	1.59	1.81	1.64	1.06	1.02	1.03	1.02	1.03	1.06	1.04	0.86	0.94	0.94	0.83
**Th**	1.78	1.65	1.73	1.76	1.64	1.75	1.54	1.63	1.43	1.69	1.55	1.52	1.59	1.53	1.62	1.46	0.91	0.98	0.95	0.92	0.95	0.90	0.90	0.98	0.86	0.82	0.80
**U**	0.54	0.51	0.54	0.56	0.53	0.52	0.51	0.49	0.49	0.54	0.45	0.50	0.51	0.48	0.49	0.48	0.32	0.32	0.32	0.31	0.28	0.32	0.29	0.29	0.27	0.26	0.24
**n (grains)**	3	3	3	3	3	3	3	2	3	3	2	3	3	3	3	3	3	3	3	3	3	3	3	3	3	3	3

EPMA at Victoria University of Wellington, cross-checked with analyses from Refs 3, 4 of Table 1.

LA-ICMPA at U. of Otago, 2014.

**Table 4:** The 1979 Surtsey Drill Core (Sideromelane Glass).

Depth (m)	19.3	48.85	140-141	150-151	158-159	169-170	170.3	176-177.7
***EPMA (wt%)***								
**SiO**_**2**_	46.72	47.03	47.00	46.83	46.69	46.61	46.20	46.27
**TiO**_**2**_	2.63	2.66	2.57	2.61	2.85	2.94	2.83	2.95
**Al**_**2**_**O**_**3**_	16.45	16.20	16.63	16.50	16.36	16.37	16.20	16.30
**FeO**^**t**^	12.02	12.18	11.83	12.25	12.77	12.94	12.79	12.96
**MnO**	0.18	0.19	0.19	0.19	0.20	0.20	0.20	0.19
**MgO**	5.94	5.98	6.08	6.11	5.84	5.56	5.66	5.54
**CaO**	9.79	9.60	9.76	9.74	9.37	9.19	9.30	9.21
**Na**_**2**_**O**	3.83	3.80	3.78	3.74	4.03	4.06	3.89	3.98
**K**_**2**_**O**	0.67	0.65	0.62	0.64	0.71	0.72	0.70	0.70
**Cr**_**2**_**O**_**3**_	0.02	0.01	0.02	0.02	0.01	0.01	0.01	0.01
**P**_**2**_**O**_**5**_	0.24	0.27	0.27	0.23	0.26	0.26	0.26	0.26
**SO**_**3**_	0.10	0.09	0.11	0.11	0.11	0.10	0.11	0.09
**Total**	98.61	98.67	98.86	98.97	99.18	98.98	98.16	98.47
**n (spots/grains)**	23/8	30/10	30/10	30/10	30/10	29/10	18/7	27/9
***LA-ICPMS (ppm)***								
**Sc**	29.84	27.76	29.32	27.77	28.04	27.34	28.41	26.53
**V**	280.80	264.41	274.41	273.16	279.75	278.86	275.07	279.85
**Co**	43.61	40.37	42.68	42.68	42.27	47.27	43.79	51.07
**Ni**	64.18	58.70	65.18	65.49	59.41	82.94	62.69	95.04
**Cu**	60.52	56.19	57.77	59.99	63.29	57.52	60.59	57.49
**Zn**	105.42	87.45	90.06	103.60	99.99	111.76	89.79	98.64
**Ga**	23.50	23.72	22.96	23.06	24.50	23.97	23.70	22.36
**Rb**	14.50	14.43	14.22	14.27	14.97	15.11	14.16	14.36
**Sr**	364.53	374.63	366.42	368.99	362.76	357.79	355.21	341.92
**Y**	34.65	34.32	35.83	35.30	36.90	38.14	34.65	36.15
**Zr**	195.35	195.64	196.67	199.49	204.58	215.38	195.00	205.98
**Nb**	23.54	23.55	23.67	23.43	25.04	25.53	23.83	25.34
**Ba**	163.69	176.21	165.52	170.09	172.81	174.53	165.67	174.40
**La**	18.40	18.53	18.45	18.43	19.30	19.64	17.76	18.92
**Ce**	43.49	44.10	43.60	43.90	46.00	47.63	43.89	46.13
**Pr**	5.96	5.87	5.69	5.83	6.10	6.28	5.99	6.14
**Nd**	26.72	26.04	25.97	26.74	27.88	28.25	26.88	27.54
**Sm**	6.49	6.06	6.53	6.49	6.10	6.62	6.71	6.81
**Eu**	2.20	2.17	1.98	2.25	2.22	2.28	2.11	2.31
**Gd**	6.69	7.15	7.04	6.87	6.85	7.28	7.08	7.21
**Tb**	1.04	0.97	1.13	1.17	1.15	1.18	1.06	1.12
**Dy**	6.74	6.43	6.72	6.86	6.79	7.32	6.59	6.87
**Ho**	1.37	1.27	1.17	1.33	1.42	1.43	1.31	1.34
**Er**	3.79	3.57	3.72	3.70	3.65	3.94	3.61	3.86
**Tm**	0.51	0.53	0.50	0.49	0.55	0.55	0.57	0.56
**Yb**	3.22	3.15	3.46	3.14	3.12	3.33	3.18	3.27
**Lu**	0.44	0.43	0.47	0.50	0.53	0.54	0.51	0.53
**Hf**	4.86	4.54	4.50	4.75	4.84	4.91	4.89	4.87
**Ta**	1.45	1.44	1.46	1.50	1.53	1.67	1.41	1.57
**Pb**	1.96	1.68	1.62	1.63	1.78	1.92	1.71	1.87
**Th**	1.57	1.58	1.44	1.56	1.58	1.71	1.52	1.54
**U**	0.47	0.48	0.47	0.48	0.47	0.52	0.48	0.48
**n (grains)**	3	3	3	3	3	3	2	3

EPMA at Victoria University of Wellington, cross-checked with analyses from Refs 3, 4, 5 of Table 1.

LA-ICMPA at U. of Otago, 2014.
