# Supplementary material for: The Surtsey Magma Series
Source: Sci Rep. 2015 Jun 26;5:11498. doi: 10.1038/srep11498 (PMC4481646; doi:10.1038/srep11498)
Supplement: Supplementary Information [file srep11498-s1.pdf]

Supplementary information accompanying:

The Surtsey Magma Series

C. Ian Schipper, Sveinn P. Jakobsson, James D.L. White, J. Michael Palin, Tim Bush-Marcinowski

Table S1. Crystal Chemistry

Olivine																	
**Mus. no.**	**Day**	**Eruption site**	**Date**	**Crystal**	**SiO2**	**TiO2**	**Al2O3**	**FeOt**	**MnO**	**MgO**	**CaO**	**Na2O**	**K2O**	**Cr2O5**	**NiO2**	**Total**	**Fo**
4638	47	Surtla, scoria	28-Dec-63 - 6-Jan-64	Core	38.60	0.02		19.40	0.30	41.40	0.24			0.01	0.16	100.13	79.2
4638	47	Surtla, scoria	28-Dec-63 - 6-Jan-64	Core	38.10	0.04		22.10	0.32	39.30	0.33			0.01	0.12	100.32	76.0
4638	47	Surtla, scoria	28-Dec-63 - 6-Jan-64	Core	38.40	0.04		20.10	0.28	40.90	0.29			0.04	0.19	100.24	78.4
4693	1013	Surtsey 66, lava	19.-27-Aug-66	Core	39.40	0.02		14.80	0.17	44.70	0.31			0.09	0.19	99.68	84.3
4693	1013	Surtsey 66, lava	19.-27-Aug-66	Core	34.50	0.17		41.50	0.56	22.20	0.56			0.03	0.04	99.56	48.8
4693	1013	Surtsey 66, lava	19.-27-Aug-66	Core	39.80	0.00		13.40	0.15	46.10	0.29			0.07	0.23	100.04	86.0
4693	1013	Surtsey 66, lava	19.-27-Aug-66	Core	39.30	0.01		15.90	0.21	43.80	0.30			0.06	0.18	99.76	83.1
4693	1013	Surtsey 66, lava	19.-27-Aug-66	Core	39.20	0.01		16.40	0.22	43.50	0.33			0.06	0.17	99.89	82.5
4693	1013	Surtsey 66, lava	19.-27-Aug-66	Core	39.30	0.02		17.40	0.23	42.80	0.32			0.05	0.14	100.25	81.4
9811	3	Surtsey 63, tephra	16-Nov-63	Core	40.60	0.02		14.70	0.21	46.30	0.25			0.08	0.24	102.40	84.9
9811	3	Surtsey 63, tephra	16-Nov-63	Core	39.10	0.06		22.60	0.38	39.90	0.28			0.04	0.14	102.50	75.9
9811	3	Surtsey 63, tephra	16-Nov-63	Core	38.90	0.07		23.20	0.39	39.10	0.34			0.04	0.13	102.17	75.0
9814	11	Surtsey 63, tephra	25-Nov-63	Core	39.50	0.00		15.90	0.23	44.10	0.25			0.08	0.22	100.28	83.2
9814	11	Surtsey 63, tephra	25-Nov-63	Core	39.00	0.03		20.10	0.32	40.80	0.27			0.03	0.19	100.74	78.3
9814	11	Surtsey 63, tephra	25-Nov-63	Core	39.70	0.00		15.70	0.22	44.40	0.26			0.07	0.26	100.61	83.4
9814	11	Surtsey 63, tephra	25-Nov-63	Core	38.80	0.04		20.10	0.30	40.70	0.31			0.03	0.19	100.47	78.3
9814	11	Surtsey 63, tephra	25-Nov-63	Core	39.30	0.00		16.50	0.24	43.80	0.30			0.07	0.17	100.38	82.6
9814	11	Surtsey 63, tephra	25-Nov-63	Core	39.70	0.02		15.40	0.22	44.80	0.29			0.08	0.25	100.76	83.8
9814	11	Surtsey 63, tephra	25-Nov-63	Core	38.60	0.03		21.10	0.33	40.00	0.28			0.03	0.16	100.59	77.2
9814	11	Surtsey 63, tephra	25-Nov-63	Core	37.30	0.08		28.80	0.45	33.80	0.38			0.05	0.05	100.92	67.7
9814	11	Surtsey 63, tephra	25-Nov-63	Core	38.80	0.04		20.10	0.33	40.80	0.30			0.07	0.18	100.62	78.3
9822	84	Surtsey 64, tephra	1.-10-Feb-64	Core	39.20	0.01		19.40	0.26	41.90	0.22			0.05	0.15	101.19	79.4
9822	84	Surtsey 64, tephra	1.-10-Feb-64	Core	40.00	0.00		15.90	0.22	44.90	0.25			0.09	0.20	101.56	83.4
9822	84	Surtsey 64, tephra	1.-10-Feb-64	Core	40.30	0.00		14.60	0.19	45.90	0.24			0.05	0.23	101.51	84.9
9826	258	Surtsey 64, lava	Jul-Aug-64	Core	39.40	0.01		15.00	0.20	44.41	0.27			0.05	0.16	99.50	84.1
9826	258	Surtsey 64, lava	Jul-Aug-64	Core	39.30	0.00		16.40	0.25	43.60	0.25			0.04	0.15	99.99	82.6
9826	258	Surtsey 64, lava	Jul-Aug-64	Core	39.40	0.01		15.90	0.21	43.90	0.26			0.07	0.18	99.93	83.1
9826	258	Surtsey 64, lava	Jul-Aug-64	Core	38.90	0.02		17.30	0.32	42.60	0.29			0.07	0.13	99.63	81.4
9828	471	Surtsey 64, lava	Jul-Aug-64	Core	39.30	0.01		19.20	0.27	42.30	0.31			0.00	0.14	101.53	79.7
9828	471	Surtsey 65, lava	27-Feb-65	Core	39.20	0.00		17.30	0.26	43.30	0.26			0.01	0.16	100.49	81.7
9828	471	Surtsey 65, lava	27-Feb-65	Core	38.70	0.03		21.20	0.33	40.00	0.37			0.01	0.09	100.73	77.1
9828	471	Surtsey 65, lava	27-Feb-65	Core	40.10	0.00		14.60	0.20	45.90	0.22			0.02	0.23	101.27	84.9
9828	471	Surtsey 65, lava	27-Feb-65	Core	38.90	0.03		22.40	0.34	39.50	0.46			0.01	0.07	101.71	75.9
9832	609	Syrtlingur, tephra	May-Jun 65	Core	39.70	0.00		13.20	0.16	45.80	0.21			0.04	0.23	99.34	86.1
9832	609	Syrtlingur, tephra	May-Jun 65	Core	39.40	0.02		14.40	0.22	45.10	0.27			0.08	0.23	99.72	84.8
9832	609	Syrtlingur, tephra	May-Jun 65	Core	39.20	0.01		16.30	0.25	43.80	0.30			0.03	0.17	100.06	82.7
9832	609	Syrtlingur, tephra	May-Jun 65	Core	39.50	0.01		14.30	0.21	45.20	0.27			0.07	0.22	99.78	84.9
9835	690	Syrtlingur, tephra	4-Oct-65	Core	39.40	0.01		15.30	0.21	44.60	0.27			0.07	0.21	100.07	83.9
9835	690	Syrtlingur, tephra	4-Oct-65	Core	39.50	0.01		14.30	0.20	45.30	0.25			0.06	0.21	99.83	85.0
9835	690	Syrtlingur, tephra	4-Oct-65	Core	39.30	0.04		16.60	0.27	42.80	0.37			0.05	0.15	99.58	82.1
9836	845	Jólnir, tephra	8-Mar-66	Core	39.30	0.02		15.70	0.26	44.00	0.25			0.02	0.20	99.75	83.3
9836	845	Jólnir, tephra	8-Mar-66	Core	39.20	0.02		16.50	0.25	43.20	0.29			0.02	0.20	99.68	82.4
9836	845	Jólnir, tephra	8-Mar-66	Core	39.50	0.03		15.80	0.23	44.20	0.27			0.04	0.21	100.28	83.3
9836	845	Jólnir, tephra	8-Mar-66	Core	39.20	0.04		16.60	0.24	43.10	0.32			0.02	0.18	99.70	82.2
9843	977	Jólnir, tephra	18-Jul-66	Core	39.70	0.02		14.10	0.22	45.40	0.26			0.06	0.21	99.97	85.2
9843	977	Jólnir, tephra	18-Jul-66	Core	39.70	0.04		15.50	0.19	44.50	0.31			0.06	0.16	100.46	83.7
9843	977	Jólnir, tephra	18-Jul-66	Core	39.20	0.03		16.50	0.23	43.40	0.33			0.04	0.15	99.88	82.4
9843	977	Jólnir, tephra	18-Jul-66	Core	39.30	0.02		15.90	0.23	43.80	0.28			0.04	0.21	99.78	83.1
9850	1064	Surtsey 66, lava	13-Oct-66	Core	39.60	0.02		13.40	0.17	45.90	0.26			0.07	0.26	99.68	85.9
9850	1064	Surtsey 66, lava	13-Oct-66	Core	39.70	0.03		15.60	0.21	44.50	0.28			0.06	0.21	100.59	83.6
9850	1064	Surtsey 66, lava	13-Oct-66	Core	39.40	0.04		16.30	0.25	44.10	0.30			0.05	0.20	100.64	82.8
9850	1064	Surtsey 66, lava	13-Oct-66	Core	39.60	0.02		14.80	0.18	45.10	0.26			0.05	0.23	100.24	84.5
9850	1064	Surtsey 66, lava	13-Oct-66	Core	39.80	0.02		13.30	0.17	46.30	0.26			0.07	0.29	100.21	86.1
9850	1064	Surtsey 66, lava	13-Oct-66	Core	39.00	0.03		17.80	0.23	42.40	0.36			0.04	0.15	100.01	80.9
9850	1064	Surtsey 66, lava	13-Oct-66	Core	39.50	0.03		16.40	0.24	44.00	0.31			0.05	0.18	100.71	82.7
9851	1144	Surtsey 67, lava	1.-3-Jan-67	Core	39.70	0.01		16.40	0.22	43.70	0.28			0.03	0.17	100.51	82.6
9851	1144	Surtsey 67, lava	1.-3-Jan-67	Core	39.90	0.00		16.30	0.22	44.20	0.30			0.03	0.19	101.14	82.9
9851	1144	Surtsey 67, lava	1.-3-Jan-67	Core	39.90	0.01		16.70	0.24	44.00	0.31			0.03	0.16	101.35	82.4
9851	1144	Surtsey 67, lava	1.-3-Jan-67	Core	39.50	0.00		17.90	0.27	42.90	0.33			0.04	0.13	101.07	81.0
9851	1144	Surtsey 67, lava	1.-3-Jan-67	Core	32.00	0.21		57.30	1.12	7.60	1.49			0.02	0.06	99.80	19.1
9851	1144	Surtsey 67, lava	1.-3-Jan-67	Core	39.90	0.00		15.70	0.21	44.10	0.27			0.07	0.17	100.42	83.4
9851	1144	Surtsey 67, lava	1.-3-Jan-67	Core	39.80	0.01		16.70	0.25	43.40	0.31			0.04	0.16	100.67	82.2
9851	1144	Surtsey 67, lava	1.-3-Jan-67	Core	39.60	0.01		17.30	0.25	43.30	0.33			0.03	0.17	99.99	81.7
9853	1235	Surtsey 67, lava	31-Mar-67	Core	39.70	0.00		15.50	0.19	44.70	0.27			0.07	0.24	100.66	83.7
9853	1235	Surtsey 67, lava	31-Mar-67	Core	40.50	0.02		13.80	0.20	46.80	0.27			0.05	0.18	101.82	85.8
9853	1235	Surtsey 67, lava	31-Mar-67	Core	39.50	0.02		16.50	0.20	44.20	0.24			0.03	0.24	100.97	82.7
9853	1235	Surtsey 67, lava	31-Mar-67	Core	39.80	0.02		16.10	0.19	44.70	0.24			0.05	0.26	101.35	83.2
9853	1235	Surtsey 67, lava	31-Mar-67	Core	39.80	0.02		17.20	0.24	44.10	0.29			0.08	0.20	101.83	82.0
9854	1285	Surtsey 67, lava	19-May-67	Core	39.50	0.00		13.50	0.17	45.50	0.25			0.08	0.29	99.28	85.7
9854	1285	Surtsey 67, lava	19-May-67	Core	39.80	0.00		12.80	0.18	46.50	0.25			0.06	0.28	99.97	86.6
9854	1285	Surtsey 67, lava	19-May-67	Core	39.30	0.00		14.80	0.17	44.30	0.28			0.04	0.22	99.11	84.2
9854	1285	Surtsey 67, lava	19-May-67	Core	39.40	0.01		16.00	0.21	43.90	0.28			0.06	0.20	100.06	83.0
4693	1013	Surtsey 66, lava	19.-27-Aug-66	Edge	38.40	0.06		21.80	0.32	39.10	0.50			0.06	0.10	100.34	76.2
9826	258	Surtsey 64, lava	Jul-Aug-64	Edge	35.00	0.14		39.80	0.74	23.60	0.62			0.01	0.21	100.12	51.4
9832	609	Syrtlingur, tephra	May-Jun 65	Edge	39.10	0.02		15.70	0.19	43.90	0.29			0.03	0.19	99.42	83.3
9836	845	Jólnir, tephra	8-Mar-66	Edge	39.10	0.05		16.50	0.24	43.00	0.38			0.03	0.15	99.45	82.3
9843	977	Jólnir, tephra	18-Jul-66	Edge	39.30	0.01		16.30	0.24	43.60	0.32			0.03	0.20	100.00	82.7
9850	1064	Surtsey 66, lava	13-Oct-66	Edge	38.70	0.07		20.20	0.29	40.80	0.44			0.05	0.15	100.70	78.3
9850	1064	Surtsey 66, lava	13-Oct-66	Edge	37.30	0.08		25.70	0.37	35.40	0.49			0.07	0.09	99.50	71.1
9851	1144	Surtsey 67, lava	1.-3-Jan-67	Edge	39.10	0.05		19.80	0.31	41.00	0.43			0.03	0.12	100.84	78.7
9843	977	Jólnir, tephra	18-Jul-66		38.20	0.08		20.10	0.31	39.00	0.68			0.03	0.12	98.52	77.6
9843	977	Jólnir, tephra	18-Jul-66		39.00	0.09		19.60	0.27	37.50	1.80			0.00		98.51	77.3
9843	977	Jólnir, tephra	18-Jul-66		39.20	0.31		18.80	0.23	37.90	1.78			0.02		98.24	78.2
9854	1285	Surtsey 67, lava	19-May-67		38.90	0.01		17.70	0.28	42.30	0.38			0.08	0.19	99.94	81.0
9854	1285	Surtsey 67, lava	19-May-67		38.50	0.04		20.10	0.31	40.40	0.45			0.04	0.13	99.97	78.2

Table S1. Crystal Chemistry (cont.)

Plagioclase

Mus. no.	Day	Eruption site	Date	Crystal	SiO2	TiO2	Al2O3	FeOt	MnO	MgO	CaO	Na2O	K2O	Cr2O5	NiO2	Total	Ab	An	Or
9814	11	Surtsey 63, tephra	25-Nov-63	Core	55.60		27.60	0.35		0.03	9.80	5.53	0.27			99.18	49.7	48.7	1.6
9854	1285	Surtsey 67, lava	19-May-67	Edge	54.50		27.60	0.79		0.10	10.90	5.12	0.26			99.27	45.3	53.2	1.5
9811	3	Surtsey 63, tephra	16. nóv. 63; "Séstey"		53.00		30.10	0.42		0.08	11.30	4.38	0.14			99.42	40.9	58.3	0.9
9814	11	Surtsey 63, tephra	25-Nov-63	Core	53.20		29.40	0.40		0.07	12.00	4.57	0.19			99.83	40.3	58.5	1.1
16449		Surtsey 63, tephra	drill core	Core	52.60		29.80	0.00		0.17	11.40	4.26	0.15			98.38	40.0	59.1	0.9
9853	1235	Surtsey 67, lava	31-Mar-67	Core	52.70		29.60	0.79		0.25	11.70	4.24	0.18			99.46	39.2	59.7	1.1
9826	258	Surtsey 64, lava	Jul-Aug-64	Edge	52.40		29.90	0.82		0.15	12.30	4.36	0.18			100.11	38.7	60.3	1.1
9814	11	Surtsey 63, tephra	25-Nov-63	Core	52.20		29.70	0.45		0.08	12.60	4.18	0.18			99.39	37.1	61.8	1.1
1986		Surtsey, phenocryst	loose in tephra	Core	51.80		30.10	0.43		0.08	12.50	4.03	0.17			99.11	36.5	62.5	1.0
16449		Surtsey 63, tephra	drill core	Core	51.70		30.20	1.10		0.19	12.20	3.93	0.15			99.47	36.5	62.6	0.9
10714		Surtsey, phenocryst	loose in tephra	Core	52.10		30.00	0.43		0.07	12.70	4.00	0.14			99.44	36.0	63.2	0.8
9828	471	Surtsey 65, lava	27-Feb-65	Core	51.60		30.50	0.89		0.20	12.30	3.76	0.12			99.37	35.4	63.9	0.7
9811	3	Surtsey 63, tephra	16. nóv. 63; "Séstey"	Core	51.90		31.00	0.81		0.12	12.50	3.77	0.10			100.20	35.1	64.3	0.6
9811	3	Surtsey 63, tephra	16. nóv. 63; "Séstey"	Core	52.10		31.20	0.67		0.13	12.40	3.68	0.11			100.29	34.7	64.6	0.7
4638	47	Surtla, scoria	28-Dec-63 - 6-Jan-64	Core	51.60		30.50	0.82		0.15	12.80	3.76	0.14			99.77	34.4	64.7	0.8
9826	258	Surtsey 64, lava	Jul-Aug-64	Core	51.60		30.70	0.69		0.15	13.20	3.85	0.14			100.33	34.3	64.9	0.8
9851	1144	Surtsey 67, lava	1.-3-Jan-67	Edge	51.90		30.60	0.84		0.24	12.80	3.70	0.10			100.18	34.1	65.3	0.6
4693	1013	Surtsey 66, lava	19.-27-Aug-66	Edge	51.00		30.50	0.61		0.19	13.40	3.83	0.10			99.63	33.9	65.5	0.6
4693	1013	Surtsey 66, lava	19.-27-Aug-66	Edge	51.50		29.70	1.35		0.29	13.30	3.79	0.09			100.02	33.8	65.6	0.5
4693	1013	Surtsey 66, lava	19.-27-Aug-66	Core	51.20		30.70	0.63		0.15	13.30	3.75	0.12			99.85	33.5	65.7	0.7
4693	1013	Surtsey 66, lava	19.-27-Aug-66	Core	51.10		30.90	0.80		0.24	13.60	3.75	0.10			100.49	33.1	66.3	0.6
9851	1144	Surtsey 67, lava	1.-3-Jan-67	Edge	51.80		30.70	0.60		0.23	13.10	3.55	0.10			100.08	32.7	66.7	0.6
9843	977	Jólnir, tephra	18-Jul-66		51.30		30.20	0.86		0.26	13.80	3.72	0.08			100.22	32.6	66.9	0.5
9843	977	Jólnir, tephra	18-Jul-66	Core	51.30		30.20	0.86		0.26	13.80	3.72	0.08			100.22	32.6	66.9	0.5
9853	1235	Surtsey 67, lava	31-Mar-67	Core	50.70		31.40	0.48		0.17	13.40	3.40	0.11			99.66	31.3	68.1	0.7
4638	47	Surtla, scoria	28-Dec-63 - 6-Jan-64	Core	50.50		30.90	0.70		0.12	13.60	3.43	0.10			99.35	31.1	68.3	0.6
9826	258	Surtsey 64, lava	Jul-Aug-64	Core	50.40		31.30	0.68		0.17	14.00	3.42	0.12			100.09	30.4	68.9	0.7
9854	1285	Surtsey 67, lava	19-May-67	Edge	50.70		30.30	0.72		0.23	14.20	3.48	0.10			99.73	30.5	68.9	0.6
9850	1064	Surtsey 66, lava	13-Oct-66	Core	50.90		31.00	0.59		0.24	14.00	3.43	0.01			100.17	30.7	69.2	0.1
9822	84	Surtsey 64, tephra	1.-10-Feb-64	Core	50.30		31.60	0.60		0.16	13.40	3.21	0.10			99.37	30.1	69.3	0.6
9822	84	Surtsey 64, tephra	1.-10-Feb-64	Edge	50.40		31.50	0.75		0.13	13.50	3.22	0.10			99.60	30.0	69.4	0.6
9853	1235	Surtsey 67, lava	31-Mar-67	Core	50.50		31.60	0.47		0.17	13.50	3.22	0.10			99.56	30.0	69.4	0.6
9851	1144	Surtsey 67, lava	1.-3-Jan-67	Core	50.70		31.30	0.60		0.23	13.70	3.25	0.11			99.89	29.8	69.5	0.7
9850	1064	Surtsey 66, lava	13-Oct-66	Core	50.60		31.20	0.63		0.21	14.00	3.32	0.10			100.06	29.8	69.6	0.6
9822	84	Surtsey 64, tephra	1.-10-Feb-64	Core	50.10		31.50	0.70		0.17	13.50	3.21	0.08			99.26	29.9	69.6	0.5
9851	1144	Surtsey 67, lava	1.-3-Jan-67		50.40		31.30	0.52		0.19	13.60	3.16	0.07			99.24	29.5	70.1	0.4
9851	1144	Surtsey 67, lava	1.-3-Jan-67		50.80		31.10	0.60		0.22	13.70	3.14	0.11			99.67	29.1	70.2	0.7
9850	1064	Surtsey 66, lava	13-Oct-66	Core	50.40		31.30	0.60		0.22	14.10	3.18	0.10			99.90	28.8	70.6	0.6
9850	1064	Surtsey 66, lava	13-Oct-66	Core	50.30		31.30	0.55		0.22	14.10	3.17	0.09			99.73	28.8	70.7	0.5
9836	845	Jólnir, tephra	8-Mar-66	Core	50.50		30.80	0.83		0.23	14.50	3.26	0.06			100.18	28.8	70.8	0.3
9851	1144	Surtsey 67, lava	1.-3-Jan-67	Core	50.90		31.70	0.59		0.19	13.90	3.10	0.09			100.47	28.6	70.9	0.5
9843	977	Jólnir, tephra	18-Jul-66	Core	50.80		31.00	0.80		0.20	14.50	3.26	0.05			100.61	28.8	70.9	0.3
9836	845	Jólnir, tephra	8-Mar-66	Core	50.60		30.90	0.85		0.24	14.60	3.24	0.07			100.50	28.5	71.1	0.4
9832	609	Syrtlingur	May-Jun 65	Core	49.70		30.70	0.72		0.22	14.70	3.14	0.07			99.25	27.8	71.8	0.4
9843	977	Jólnir, tephra	18-Jul-66	Edge	50.10		30.90	0.68		0.25	14.80	3.14	0.05			99.92	27.7	72.0	0.3
9836	845	Jólnir, tephra	8-Mar-66	Core	50.10		30.90	0.70		0.18	14.90	3.15	0.05			99.98	27.6	72.1	0.3
9851	1144	Surtsey 67, lava	1.-3-Jan-67	Core	50.40		31.80	0.59		0.20	14.20	2.92	0.08			100.19	27.0	72.5	0.5
9854	1285	Surtsey 67, lava	19-May-67	Core	49.60		31.00	0.60		0.17	15.00	3.01	0.09			99.47	26.5	73.0	0.5
9851	1144	Surtsey 67, lava	1.-3-Jan-67		50.80		32.00	0.65		0.21	14.20	2.83	0.09			100.78	26.4	73.1	0.6
9843	977	Jólnir, tephra	18-Jul-66	Core	49.90		30.90	0.78		0.27	14.90	2.98	0.06			99.79	26.5	73.2	0.4
9828	471	Surtsey 65, lava	27-Feb-65	Core	49.30		32.00	0.55		0.15	14.10	2.77	0.06			98.93	26.1	73.5	0.4
9832	609	Syrtlingur	May-Jun 65	Core	49.30		31.60	0.61		0.20	15.30	2.99	0.06			100.06	26.0	73.6	0.3
9843	977	Jólnir, tephra	18-Jul-66	Core	50.20		31.10	0.63		0.20	14.70	2.82	0.06			99.71	25.7	74.0	0.4
9843	977	Jólnir, tephra	18-Jul-66	Core	49.30		31.60	0.63		0.21	15.30	2.91	0.04			99.99	25.5	74.2	0.2
9851	1144	Surtsey 67, lava	1.-3-Jan-67	Edge	50.00		32.20	0.60		0.20	14.80	2.72	0.09			100.61	24.8	74.6	0.5
9854	1285	Surtsey 67, lava	19-May-67	Core	49.60		31.40	0.63		0.22	15.30	2.81	0.08			100.04	24.8	74.7	0.5
9854	1285	Surtsey 67, lava	19-May-67	Core	49.00		31.60	0.60		0.17	15.50	2.71	0.07			99.65	23.9	75.7	0.4
1986		Surtsey, phenocryst	loose in tephra	Edge	49.10		32.10	0.56		0.11	15.30	2.61	0.09			99.87	23.5	76.0	0.5
9851	1144	Surtsey 67, lava	1.-3-Jan-67		49.60		32.50	0.57		0.19	15.10	2.56	0.09			100.61	23.3	76.1	0.5
9826	258	Surtsey 64, lava	Jul-Aug-64	Core	48.80		32.60	0.54		0.14	15.40	2.57	0.06			100.11	23.1	76.5	0.4
9851	1144	Surtsey 67, lava	1.-3-Jan-67		49.70		32.40	0.55		0.17	14.80	2.46	0.06			100.14	23.0	76.6	0.4
9853	1235	Surtsey 67, lava	31-Mar-67	Core	48.90		32.90	0.58		0.15	14.50	2.39	0.06			99.48	22.9	76.7	0.4
9835	690	Syrtlingur	4-Oct-65	Core	48.70		31.90	0.61		0.18	15.70	2.58	0.06			99.73	22.8	76.8	0.3
9851	1144	Surtsey 67, lava	1.-3-Jan-67	Core	49.10		32.40	0.72		0.17	15.00	2.44	0.07			99.90	22.6	76.9	0.4
9835	690	Syrtlingur	4-Oct-65	Core	48.80		31.80	0.61		0.17	15.60	2.55	0.05			99.58	22.8	76.9	0.3
4693	1013	Surtsey 66, lava	19.-27-Aug-66	Core	48.50		32.80	0.74		0.19	15.70	2.55	0.04			100.52	22.7	77.1	0.2
9851	1144	Surtsey 67, lava	1.-3-Jan-67	Core	49.00		32.90	0.60		0.15	15.10	2.34	0.08			100.17	21.8	77.7	0.5
9832	609	Syrtlingur	May-Jun 65	Core	48.40		32.10	0.56		0.18	16.00	2.49	0.05			99.78	21.9	77.8	0.3
4693	1013	Surtsey 66, lava	19.-27-Aug-66	Edge	48.30		32.50	0.65		0.18	15.80	2.41	0.06			99.90	21.6	78.1	0.4
4693	1013	Surtsey 66, lava	19.-27-Aug-66	Core	48.20		32.50	0.51		0.20	15.80	2.37	0.04			99.62	21.3	78.5	0.2
9835	690	Syrtlingur	4-Oct-65	Core	48.20		32.20	0.68		0.16	16.20	2.35	0.06			99.85	20.7	78.9	0.3
9851	1144	Surtsey 67, lava	1.-3-Jan-67	Core	48.60		32.90	0.62		0.19	15.40	2.11	0.06			99.88	19.8	79.8	0.4
9843	977	Jólnir, tephra	18-Jul-66	Core	45.40		34.60	0.76		0.10	18.50	0.84	0.03			100.23	7.6	92.2	0.2

Clinopyroxene

Mus. no.	Day	Eruption site	Date	Crystal	SiO2	TiO2	Al2O3	FeOt	MnO	MgO	CaO	Na2O	K2O	Cr2O5	NiO2	Total	Wo	En	Fer
9843	977	Jólnir, tephra	18-Jul-66		47.50	1.96	5.89	7.30	0.18	14.00	20.50	0.35		1.11		98.79	44.9	42.6	12.5
9843	977	Jólnir, tephra	18-Jul-66		49.50	1.41	4.79	8.20	0.17	15.40	19.30	0.33		0.47		99.57	41.0	45.5	13.6

Major element analyses by EPMA at USGS Menlo Park, 1981

Table S2. Major- (EPMA) and trace- (LA-ICPMS) element analyses of individual tephra shards

EPMA No.	SiO2	TiO2	Al2O3	FeO	MnO	MgO	CaO	Na2O	K2O	Cr2O3	P2O5	SO3	Total	n
S-9809-01	46.03 (0.12)	3.04 (0.04)	15.65 (0.24)	13.5 (0.2)	0.22 (0.05)	5.46 (0.13)	9.09 (0.12)	4.09 (0.12)	0.8 (0.06)	0.01 (0)	0.27 (0.04)	0.13 (0.04)	98.29	3
S-9809-02	46.04 (0.58)	3.18 (0.04)	15.41 (0.15)	13.51 (0.25)	0.19 (0.02)	5.22 (0.12)	9.1 (0.08)	4.22 (0.06)	0.86 (0.01)	0.01 (0.01)	0.29 (0.02)	0.14 (0.02)	98.17	3
S-9809-03	46.16 (0.38)	2.91 (0.07)	16.04 (0.13)	13.16 (0.3)	0.18 (0)	5.47 (0.04)	9.15 (0.11)	4.03 (0.14)	0.79 (0.01)	0.01 (0.01)	0.29 (0.01)	0.13 (0.01)	98.32	3
S-9809-04	46.11 (0.51)	3.18 (0.07)	15.37 (0.2)	13.57 (0.5)	0.21 (0.03)	5.22 (0.15)	9.09 (0.02)	4.16 (0.13)	0.89 (0.07)	0.01 (0.01)	0.32 (0.05)	0.14 (0.01)	98.28	3
S-9809-05	46.06 (0.18)	3.17 (0.06)	15.36 (0.15)	13.74 (0.15)	0.22 (0.03)	5.13 (0.1)	9.16 (0.01)	4.13 (0.14)	0.88 (0.02)	0 (0.01)	0.3 (0.03)	0.14 (0)	98.30	3
S-9809-06	46.45 (0.52)	3.05 (0.03)	15.96 (0.08)	13.42 (0.11)	0.21 (0.01)	5.5 (0.17)	9.18 (0.07)	4.14 (0.02)	0.85 (0.07)	0.02 (0.02)	0.28 (0.05)	0.13 (0.01)	99.18	3
S-9809-07	45.75 (0.26)	3.32 (0.07)	14.75 (0.36)	14.3 (0.29)	0.22 (0.01)	5.25 (0.35)	9.06 (0.16)	4.33 (0.07)	1 (0.03)	0 (0)	0.29 (0.02)	0.14 (0)	98.40	3
S-9809-08	46.36 (0.22)	2.85 (0.02)	16.3 (0.22)	12.74 (0.15)	0.19 (0.02)	5.52 (0.13)	9.26 (0.1)	4.08 (0.16)	0.8 (0.06)	0.02 (0.01)	0.29 (0.04)	0.12 (0.02)	98.52	3
S-9809-09	46.37 (0.37)	2.91 (0.03)	16.32 (0.17)	12.83 (0.07)	0.19 (0.01)	5.45 (0.12)	9.22 (0.08)	4.03 (0.19)	0.8 (0.03)	0.02 (0.02)	0.28 (0.04)	0.12 (0.03)	98.55	3
S-9809-10	46.46 (0.33)	3.1 (0.05)	15.61 (0.26)	13.42 (0.07)	0.19 (0.02)	5.33 (0.07)	9.04 (0.19)	4.11 (0.06)	0.89 (0.06)	0.01 (0.02)	0.31 (0.02)	0.13 (0.02)	98.61	3
**S-9809**	**46.18 (0.23)**	**3.07 (0.15)**	**15.68 (0.49)**	**13.42 (0.44)**	**0.2 (0.02)**	**5.36 (0.14)**	**9.14 (0.07)**	**4.13 (0.09)**	**0.86 (0.06)**	**0.01 (0.01)**	**0.29 (0.01)**	**0.13 (0.01)**	**98.46**	**30**
S-9810-01	46.08 (0.34)	3.14 (0.04)	15.51 (0.27)	13.51 (0.16)	0.21 (0)	5.39 (0.17)	9.13 (0.12)	4.15 (0.07)	0.84 (0.04)	0.02 (0.01)	0.31 (0.04)	0.12 (0.02)	98.42	3
S-9810-02	46.03 (0.5)	2.8 (0.01)	16.07 (0.18)	13.16 (0.5)	0.19 (0.01)	5.61 (0.27)	9.28 (0.05)	4.03 (0.2)	0.79 (0.02)	0.02 (0.01)	0.28 (0.02)	0.14 (0.03)	98.39	3
S-9810-03	46.23 (0.13)	2.83 (0.04)	16.29 (0.22)	12.89 (0.06)	0.17 (0.01)	5.49 (0.14)	9.24 (0.13)	3.96 (0.14)	0.78 (0.01)	0.02 (0.03)	0.26 (0.03)	0.15 (0.01)	98.32	3
S-9810-04	46.63 (0.17)	2.94 (0.06)	16.19 (0.21)	12.78 (0.24)	0.21 (0.04)	5.43 (0.17)	9.21 (0.13)	3.88 (0.06)	0.83 (0.03)	0.02 (0)	0.27 (0.02)	0.12 (0.01)	98.51	3
S-9810-05	46.43 (0.23)	2.95 (0.02)	16.07 (0.22)	12.81 (0.57)	0.17 (0.03)	5.49 (0.25)	9.32 (0.01)	4.03 (0.08)	0.81 (0.03)	0.02 (0.02)	0.27 (0.05)	0.14 (0.04)	98.50	3
S-9810-06	46.33 (0.52)	2.97 (0.03)	16.03 (0.24)	13.08 (0.3)	0.2 (0.04)	5.5 (0.1)	9.17 (0.04)	4.05 (0.08)	0.85 (0.07)	0.01 (0.01)	0.27 (0.03)	0.13 (0.01)	98.58	3
S-9810-07	46.48 (0.28)	3.21 (0.02)	15.48 (0.27)	13.63 (0.11)	0.23 (0.02)	5.17 (0.18)	9.14 (0.05)	4.17 (0.16)	0.88 (0.04)	0.01 (0.01)	0.29 (0.03)	0.15 (0.02)	98.83	3
S-9810-08	46.36 (0.21)	2.99 (0.07)	16.01 (0.5)	13.16 (0.59)	0.17 (0.01)	5.44 (0.14)	9.2 (0.09)	3.95 (0.19)	0.82 (0.01)	0 (0.01)	0.28 (0.03)	0.14 (0)	98.52	3
S-9810-09	46.32 (0.09)	3.07 (0.06)	15.63 (0.17)	13.36 (0.62)	0.2 (0.03)	5.4 (0.21)	9.17 (0.04)	4.13 (0.22)	0.84 (0.03)	0.02 (0)	0.28 (0.04)	0.13 (0.03)	98.56	3
S-9810-10	46.21 (0.08)	3.11 (0.06)	15.52 (0.19)	13.51 (0.22)	0.18 (0.02)	5.48 (0.19)	9.15 (0.05)	4.16 (0.11)	0.85 (0.04)	0.01 (0.02)	0.3 (0.08)	0.14 (0.02)	98.62	3
**S-9810**	**46.31 (0.18)**	**3 (0.13)**	**15.88 (0.31)**	**13.19 (0.31)**	**0.19 (0.02)**	**5.44 (0.11)**	**9.2 (0.06)**	**4.05 (0.1)**	**0.83 (0.03)**	**0.02 (0.01)**	**0.28 (0.02)**	**0.14 (0.01)**	**98.53**	**30**
S-11236-01	46.65 (0.25)	2.87 (0.04)	16.27 (0.1)	12.85 (0.22)	0.17 (0.01)	5.51 (0.08)	9.21 (0.08)	3.89 (0.04)	0.78 (0.01)	0.01 (0.02)	0.25 (0.01)	0.13 (0.01)	98.59	2
S-11236-02	46.31 (0.48)	3.2 (0.07)	15.32 (0.32)	14.09 (0.4)	0.19 (0.05)	5.17 (0.2)	9.21 (0.11)	4.01 (0.2)	0.86 (0.05)	0.01 (0.01)	0.29 (0.05)	0.14 (0.02)	98.81	3
S-11236-03	46.45 (0.15)	3.23 (0.02)	15.6 (0.16)	13.88 (0.23)	0.23 (0.02)	5.2 (0.07)	9.11 (0.12)	4.23 (0.13)	0.88 (0.07)	0.02 (0.03)	0.31 (0.06)	0.15 (0)	99.29	3
S-11236-04	46.51 (0.19)	2.93 (0.03)	16.28 (0.05)	12.88 (0.04)	0.19 (0.02)	5.5 (0.2)	9.33 (0.05)	4.14 (0.17)	0.79 (0.02)	0.01 (0.01)	0.26 (0.02)	0.13 (0.02)	98.96	3
S-11236-05	46.16 (0.31)	2.98 (0.04)	16.01 (0.31)	13.29 (0.13)	0.22 (0.01)	5.46 (0.17)	9.22 (0.11)	4.03 (0.16)	0.82 (0.04)	0.01 (0.02)	0.26 (0.04)	0.14 (0.01)	98.60	3
S-11236-06	46.31 (0.16)	2.96 (0)	16.03 (0.22)	12.86 (0.17)	0.19 (0.01)	5.47 (0.11)	9.21 (0.02)	4 (0.08)	0.83 (0.04)	0.01 (0.01)	0.28 (0.07)	0.12 (0.02)	98.27	3
S-11236-07	46.32 (0.21)	3.2 (0.02)	15.5 (0.07)	13.55 (0.03)	0.21 (0.01)	5.19 (0.2)	9.17 (0.06)	4.18 (0.12)	0.88 (0.05)	0.01 (0.01)	0.28 (0.06)	0.14 (0)	98.63	3
S-11236-08	46.28 (0.36)	2.9 (0.04)	16.15 (0.23)	12.8 (0.21)	0.19 (0.04)	5.54 (0.1)	9.16 (0.09)	3.91 (0.06)	0.79 (0.01)	0.02 (0.01)	0.28 (0.01)	0.14 (0)	98.16	3
S-11236-09	46.42 (0.07)	2.93 (0.06)	16.09 (0.11)	13.21 (0.03)	0.18 (0.05)	5.46 (0.14)	9.2 (0.11)	4 (0.19)	0.82 (0.02)	0 (0.01)	0.29 (0.05)	0.13 (0.02)	98.73	3
S-11236-10	46.37 (0.22)	3.01 (0.04)	15.95 (0.05)	13.29 (0.01)	0.2 (0.03)	5.39 (0.07)	9.15 (0.03)	4.04 (0.07)	0.84 (0.01)	0.01 (0.01)	0.29 (0.02)	0.13 (0.01)	98.68	3
**S-11236**	**46.38 (0.14)**	**3.02 (0.14)**	**15.92 (0.33)**	**13.27 (0.46)**	**0.2 (0.02)**	**5.39 (0.15)**	**9.2 (0.06)**	**4.04 (0.11)**	**0.83 (0.04)**	**0.01 (0.01)**	**0.28 (0.02)**	**0.13 (0.01)**	**98.67**	**29**
S-9811-01	46.36 (0.16)	2.98 (0.06)	16.05 (0.19)	13.16 (0.36)	0.2 (0)	5.77 (0.13)	9.2 (0.1)	4.15 (0.21)	0.82 (0.02)	0.02 (0.03)	0.25 (0.08)	0.12 (0.02)	99.07	3
S-9811-02	46.53 (0.16)	2.86 (0.01)	16.28 (0.26)	12.78 (0.29)	0.17 (0.01)	5.59 (0.16)	9.23 (0.02)	4.16 (0.08)	0.78 (0.01)	0.01 (0.01)	0.29 (0.03)	0.12 (0.01)	98.80	3
S-9811-03	46.27 (0.09)	3.13 (0.05)	15.62 (0.14)	13.76 (0.14)	0.21 (0.02)	5.42 (0.11)	9.02 (0.1)	4.08 (0.13)	0.9 (0.06)	0.02 (0.03)	0.33 (0.04)	0.11 (0.02)	98.88	3
S-9811-04	46.28 (0.02)	3.21 (0.01)	15.39 (0.13)	13.44 (0.27)	0.21 (0.04)	5.31 (0.22)	9.14 (0.04)	4.12 (0.05)	0.84 (0.02)	0 (0)	0.34 (0.07)	0.15 (0.01)	98.44	3
S-9811-05	46.42 (0.21)	3.02 (0.08)	16.04 (0.22)	13.22 (0.06)	0.2 (0.05)	5.55 (0.2)	9.21 (0.06)	4.12 (0.04)	0.82 (0.04)	0 (0.01)	0.26 (0.03)	0.12 (0.02)	98.98	3
S-9811-06	46.32 (0.09)	3.14 (0.02)	15.54 (0.1)	13.52 (0.29)	0.21 (0.05)	5.47 (0.07)	9.05 (0.08)	4.17 (0.21)	0.87 (0.03)	0.01 (0.02)	0.29 (0.03)	0.14 (0.01)	98.72	3
S-9811-07	45.98 (0.6)	3.22 (0.01)	15.23 (0.29)	13.44 (0.43)	0.22 (0.01)	5.11 (0.13)	9.02 (0.12)	4.19 (0.06)	0.89 (0.05)	0 (0)	0.28 (0.01)	0.14 (0.03)	97.72	3
S-9811-08	46.91 (0.21)	2.82 (0.06)	16.5 (0.07)	12.94 (0.2)	0.19 (0.03)	5.63 (0.15)	9.27 (0.04)	4.06 (0.14)	0.79 (0.06)	0.01 (0.01)	0.27 (0.09)	0.1 (0.02)	99.49	3
S-9811-09	46.7 (0.13)	2.94 (0.01)	16.25 (0.26)	12.97 (0.12)	0.18 (0.02)	5.58 (0.17)	9.22 (0.05)	4.12 (0.13)	0.79 (0.05)	0.01 (0.01)	0.26 (0.06)	0.11 (0.02)	99.13	3
S-9811-10	46.64 (0.32)	2.86 (0.01)	16.47 (0.12)	12.85 (0.48)	0.19 (0.02)	5.6 (0.11)	9.26 (0.09)	4.09 (0.14)	0.78 (0.05)	0.01 (0.01)	0.27 (0.02)	0.12 (0)	99.13	3
**S-9811**	**46.44 (0.26)**	**3.02 (0.15)**	**15.94 (0.46)**	**13.21 (0.32)**	**0.2 (0.01)**	**5.5 (0.18)**	**9.16 (0.1)**	**4.13 (0.04)**	**0.83 (0.05)**	**0.01 (0.01)**	**0.28 (0.03)**	**0.12 (0.01)**	**98.84**	**30**
S-801-01	46.79 (0.52)	2.93 (0.06)	15.99 (0.18)	12.98 (0.11)	0.19 (0.03)	5.57 (0.11)	9.12 (0.1)	4.11 (0.06)	0.83 (0.03)	0 (0)	0.28 (0.04)	0.13 (0.01)	98.91	3
S-801-02	46.39 (0.13)	2.96 (0.03)	16.18 (0.24)	12.97 (0.14)	0.19 (0.03)	5.47 (0.06)	9.16 (0.04)	4.07 (0.17)	0.82 (0.05)	0.02 (0.01)	0.28 (0.04)	0.14 (0.03)	98.66	3
S-801-03	46.47 (0.18)	2.85 (0.05)	16.33 (0.28)	12.28 (0.61)	0.2 (0)	5.56 (0.12)	9.24 (0.07)	4.07 (0.18)	0.8 (0.03)	0 (0.01)	0.3 (0.08)	0.13 (0.01)	98.22	3
S-801-04	46.34 (0.61)	2.95 (0.03)	16.04 (0.52)	13.04 (0.23)	0.17 (0.05)	5.41 (0.08)	9.12 (0.08)	3.97 (0.08)	0.79 (0.02)	0.02 (0.02)	0.31 (0.05)	0.16 (0.02)	98.30	3
S-801-05	46.23 (0.68)	2.86 (0.08)	16.22 (0.39)	12.67 (0.32)	0.19 (0.05)	5.48 (0.09)	9.2 (0.01)	3.97 (0.05)	0.76 (0.06)	0.02 (0)	0.25 (0.02)	0.11 (0.02)	97.97	3
S-801-06	46.16 (0.4)	2.94 (0.04)	15.91 (0.51)	13.01 (0.59)	0.19 (0.04)	5.45 (0.14)	9.13 (0.12)	3.97 (0.04)	0.82 (0.06)	0 (0.01)	0.26 (0.02)	0.11 (0.01)	97.95	3
S-801-07	46.4 (0.26)	2.96 (0.03)	16.11 (0.27)	13.26 (0.29)	0.19 (0.02)	5.49 (0.04)	9.17 (0.01)	4.08 (0.06)	0.81 (0.02)	0 (0.01)	0.27 (0.01)	0.13 (0.03)	98.87	3
S-801-08	46.39 (0.31)	2.92 (0.02)	16.09 (0.11)	13.1 (0.45)	0.2 (0.01)	5.48 (0.11)	9.09 (0.07)	4.12 (0.13)	0.83 (0.04)	0.01 (0.02)	0.27 (0.08)	0.14 (0.05)	98.63	3
S-801-09	46.4 (0.46)	2.89 (0.04)	16.28 (0.31)	12.83 (0.26)	0.18 (0.01)	5.61 (0.03)	9.17 (0.05)	4.08 (0.04)	0.82 (0.05)	0.01 (0.01)	0.28 (0.05)	0.15 (0.02)	98.69	3
S-801-10	46.45 (0.08)	3.22 (0.03)	15.49 (0.35)	13.52 (0.2)	0.2 (0.03)	5.26 (0.19)	9.07 (0.06)	4.2 (0.14)	0.91 (0.02)	0.01 (0.01)	0.32 (0.02)	0.13 (0.01)	98.79	3
**S-801**	**46.4 (0.17)**	**2.95 (0.1)**	**16.06 (0.24)**	**12.97 (0.33)**	**0.19 (0.01)**	**5.48 (0.1)**	**9.15 (0.05)**	**4.06 (0.08)**	**0.82 (0.04)**	**0.01 (0.01)**	**0.28 (0.02)**	**0.13 (0.01)**	**98.50**	**30**
S-9812-01	46.55 (0.27)	2.84 (0.04)	16.12 (0.23)	13.43 (0.08)	0.2 (0.01)	5.7 (0.02)	9.18 (0.04)	3.89 (0.09)	0.7 (0.02)	0.01 (0.01)	0.21 (0.02)	0.09 (0.01)	98.92	3
S-9812-02	46.54 (0.13)	2.82 (0.03)	15.94 (0.1)	12.82 (0.25)	0.21 (0.03)	6.06 (0.21)	9.19 (0.13)	4.06 (0.14)	0.81 (0.05)	0.03 (0.03)	0.27 (0.02)	0.13 (0.01)	98.88	3
S-9812-03	46.34 (0.23)	3.03 (0.03)	15.42 (0.17)	13.44 (0.24)	0.2 (0.02)	5.58 (0.17)	9.14 (0.04)	4.02 (0.05)	0.85 (0.05)	0.02 (0.02)	0.32 (0.02)	0.1 (0.01)	98.47	3
S-9812-04	46.22 (0.41)	3.18 (0.05)	15.44 (0.26)	13.81 (0.25)	0.17 (0.01)	5.34 (0.02)	9.07 (0.04)	4.27 (0.12)	0.88 (0.02)	0.01 (0.02)	0.29 (0.02)	0.13 (0.01)	98.80	3
S-9812-05	46.72 (0.17)	2.87 (0.04)	16.08 (0.11)	13.06 (0.07)	0.2 (0.02)	5.89 (0.09)	9.28 (0.07)	4.06 (0.16)	0.79 (0.03)	0.01 (0.01)	0.27 (0.02)	0.13 (0.01)	99.34	3
S-9812-06	46.45 (0.13)	3.08 (0.02)	15.96 (0.04)	13.03 (0.46)	0.21 (0.02)	5.3 (0.3)	9.16 (0.17)	4.05 (0.22)	0.84 (0.02)	0.02 (0.02)	0.29 (0.04)	0.13 (0.02)	98.52	3
S-9812-07	46.48 (0.2)	2.86 (0.04)	16.03 (0.3)	12.72 (0.37)	0.18 (0.03)	5.82 (0.14)	9.27 (0.03)	4.02 (0.11)	0.8 (0.05)	0 (0)	0.28 (0.02)	0.12 (0.03)	98.59	3
S-9812-08	46.46 (0.19)	2.86 (0.06)	15.91 (0.11)	12.82 (0.35)	0.2 (0.01)	5.84 (0.08)	9.17 (0.06)	4.07 (0.06)	0.82 (0.05)	0 (0)	0.3 (0.05)	0.13 (0.03)	98.57	3
S-9812-09	46.55 (0.2)	2.86 (0.05)	15.92 (0.19)	12.92 (0.2)	0.2 (0.02)	5.82 (0.18)	9.27 (0.11)	3.93 (0.13)	0.8 (0.04)	0.02 (0.02)	0.3 (0.02)	0.13 (0.02)	98.71	3
S-9812-10	46.74 (0.22)	2.77 (0.02)	16.16 (0.32)	12.73 (0.3)	0.2 (0.03)	5.76 (0.16)	9.3 (0.08)	4.03 (0.11)	0.79 (0.02)	0.01 (0.01)	0.29 (0.02)	0.13 (0.02)	98.92	3
**S-9812**	**46.5 (0.16)**	**2.92 (0.13)**	**15.9 (0.26)**	**13.08 (0.37)**	**0.2 (0.01)**	**5.71 (0.24)**	**9.2 (0.07)**	**4.04 (0.1)**	**0.81 (0.05)**	**0.01 (0.01)**	**0.28 (0.03)**	**0.12 (0.01)**	**98.77**	**30**
S-9813-01	46.36 (0.08)	2.92 (0.02)	16.21 (0.17)	13.06 (0.19)	0.17 (0.01)	5.41 (0.08)	9.29 (0.02)	4.11 (0.07)	0.77 (0.02)	0.01 (0.02)	0.27 (0.05)	0.15 (0.02)	98.72	3
S-9813-02	46.4 (0.1)	2.96 (0.03)	15.83 (0.23)	13.17 (0.11)	0.2 (0.01)	5.65 (0.16)	9.38 (0.06)	4.13 (0.12)	0.83 (0.06)	0.02 (0.03)	0.29 (0.04)	0.11 (0.02)	98.96	3
S-9813-03	46.26 (0.46)	3 (0.05)	15.93 (0.22)	12.86 (0.67)	0.17 (0.02)	5.32 (0.04)	9.23 (0.12)	4.12 (0.11)	0.85 (0.04)	0 (0.01)	0.27 (0.03)	0.13 (0.01)	98.15	3
S-9813-04	46.61 (0.11)	3.07 (0.02)	15.6 (0.13)	13.11 (0.6)	0.18 (0.02)	5.54 (0.09)	9.32 (0.02)	4.27 (0.14)	0.85 (0.01)	0 (0)	0.29 (0.02)	0.11 (0.01)	98.95	3
S-9813-05	46.61 (0.27)	2.78 (0.03)	16.2 (0.45)	12.62 (0.26)	0.2 (0.05)	5.82 (0.08)	9.32 (0.01)	4.06 (0.09)	0.79 (0.02)	0.01 (0.01)	0.28 (0.02)	0.12 (0.01)	98.80	3
S-9813-06	46.52 (0.45)	2.8 (0.05)	16.14 (0.32)	12.45 (0.12)	0.18 (0.01)	5.75 (0.16)	9.33 (0.09)	4.06 (0.08)	0.78 (0.01)	0.01 (0.02)	0.29 (0.06)	0.1 (0.01)	98.40	3
S-9813-07	46.42 (0.26)	2.85 (0.06)	16.03 (0.22)	12.6 (0.45)	0.18 (0.05)	5.91 (0.16)	9.36 (0.06)	3.92 (0.02)	0.8 (0.04)	0.04 (0.01)	0.25 (0.05)	0.12 (0.02)	98.47	3
S-9813-08	46.45 (0.45)	2.88 (0.09)	15.87 (0.39)	12.66 (0.56)	0.2 (0.01)	5.86 (0.09)	9.31 (0.07)	4.08 (0.1)	0.78 (0.05)	0.02 (0.02)	0.29 (0.04)	0.11 (0.03)	98.51	3
S-9813-09	46.51 (0.09)	2.91 (0.06)	15.67 (0.12)	13.02 (0.15)	0.2 (0.03)	5.68 (0.17)	9.25 (0.06)	4.1 (0.13)	0.87 (0.07)	0.01 (0.01)	0.33 (0.03)	0.13 (0.02)	98.69	3
**S-9813**	**46.46 (0.12)**	**2.91 (0.09)**	**15.94 (0.22)**	**12.84 (0.26)**	**0.19 (0.01)**	**5.66 (0.2)**	**9.31 (0.05)**	**4.09 (0.09)**	**0.81 (0.04)**	**0.01 (0.01)**	**0.28 (0.02)**	**0.12 (0.01)**	**98.63**	**27**
S-9814-01	46.45 (0.14)	2.7 (0.02)	16.27 (0.16)	12.37 (0.12)	0.2 (0.03)	5.95 (0.1)	9.55 (0.03)	4.01 (0.13)	0.76 (0.01)	0.02 (0.01)	0.23 (0.01)	0.13 (0.01)	98.64	3
S-9814-02	46.92 (0.23)	2.73 (0.05)	16.39 (0.09)	12.98 (0.22)	0.21 (0.05)	5.9 (0.04)	9.42 (0.05)	3.76 (0.14)	0.64 (0.02)	0.01 (0.02)	0.19 (0.04)	0.11 (0.02)	99.25	3
S-9814-03	46.54 (0.12)	2.73 (0.04)	15.92 (0.03)	12.72 (0.12)	0.21 (0.04)	6 (0.21)	9.4 (0.04)	3.95 (0.06)	0.77 (0.03)	0.01 (0.01)	0.26 (0.04)	0.12 (0.02)	98.63	3
S-9814-04	46.55 (0.18)	2.92 (0.06)	15.71 (0.23)	13.52 (0.22)	0.21 (0.04)	5.75 (0.46)	9.19 (0.14)	3.83 (0.15)	0.72 (0.03)	0.01 (0.01)	0.22 (0.04)	0.07 (0.02)	98.71	3
S-9814-05	46.66 (0.27)	2.68 (0.01)	16.34 (0.25)	12.63 (0.32)	0.2 (0.02)	5.97 (0.15)	9.48 (0.05)	3.84 (0.06)	0.76 (0.02)	0.01 (0.01)	0.27 (0.04)	0.14 (0.04)	98.98	3
S-9814-06	46.59 (0.13)	2.85 (0.03)	16.01 (0.17)	12.69 (0.27)	0.19 (0.02)	5.9 (0.18)	9.28 (0.08)	4.05 (0.2)	0.8 (0.02)	0 (0.01)	0.29 (0.03)	0.11 (0.02)	98.75	3
S-9814-07	46.53 (0.14)	2.72 (0.02)	16.25 (0.27)	12.28 (0.24)	0.19 (0.04)	5.94 (0.08)	9.49 (0.04)	3.88 (0.08)	0.76 (0.06)	0.02 (0.02)	0.25 (0.03)	0.11 (0.02)	98.42	3
S-9814-08	46.27 (0.44)	2.74 (0.02)	16.07 (0.18)	12.65 (0.42)	0.17 (0.01)	6.01 (0.12)	9.27 (0.12)	4.03 (0.08)	0.81 (0.05)	0.01 (0.01)	0.28 (0.03)	0.15 (0.03)	98.46	3
S-9814-09	46.5 (0.19)	2.71 (0.03)	16.37 (0.16)	12.5 (0.32)	0.19 (0.01)	5.94 (0.11)	9.5 (0.08)	3.93 (0.07)	0.76 (0.05)	0.01 (0.02)	0.26 (0.05)	0.13 (0.01)	98.80	3
S-9814-10	46.7 (0.31)	2.71 (0.02)	16.34 (0.14)	12.73 (0.2)	0.2 (0.01)	5.88 (0.12)	9.48 (0.07)	3.89 (0.04)	0.75 (0.03)	0.02 (0.01)	0.27 (0.04)	0.12 (0.02)	99.09	3
**S-9814**	**46.57 (0.17)**	**2.75 (0.08)**	**16.17 (0.23)**	**12.71 (0.35)**	**0.2 (0.01)**	**5.92 (0.07)**	**9.41 (0.12)**	**3.92 (0.09)**	**0.75 (0.05)**	**0.01 (0.01)**	**0.25 (0.03)**	**0.12 (0.02)**	**98.77**	**30**
S-9815-01	46.59 (0.18)	2.78 (0.05)	15.91 (0.4)	12.88 (0.24)	0.19 (0.01)	5.79 (0.25)	9.45 (0.07)	4.02 (0.03)	0.8 (0.03)	0 (0)	0.26 (0.04)	0.11 (0.01)	98.79	3
S-9815-02	46.78 (0.12)	2.7 (0.03)	16.35 (0.24)	12.51 (0.08)	0.16 (0.01)	5.85 (0.13)	9.52 (0.14)	3.84 (0.22)	0.77 (0.03)	0.01 (0.01)	0.26 (0)	0.12 (0.02)	98.88	3
S-9815-03	46.8 (0.15)	2.72 (0.01)	16.07 (0.13)	12.35 (0.34)	0.19 (0.04)	5.82 (0.23)	9.47 (0.19)	3.79 (0.06)	0.78 (0.02)	0.03 (0.02)	0.25 (0.03)	0.12 (0.02)	98.39	3
S-9815-04	46.26 (0.4)	2.8 (0.04)	15.72 (0.22)	13.01 (0.12)	0.2 (0.03)	5.68 (0.14)	9.45 (0.1)	3.89 (0.12)	0.81 (0.03)	0.01 (0.02)	0.25 (0.02)	0.12 (0.01)	98.21	3
S-9815-05	46.21 (0.44)	2.95 (0.03)	15.9 (0.34)	12.9 (0.35)	0.2 (0.01)	5.26 (0.17)	9.17 (0.16)	3.83 (0.43)	0.82 (0.03)	0.01 (0.01)	0.24 (0)	0.14 (0.02)	97.64	3
S-9815-06	46.7 (0.11)	2.82 (0.04)	15.77 (0.31)	12.66 (0.13)	0.19 (0.03)	5.82 (0.16)	9.42 (0.05)	3.94 (0.06)	0.81 (0.01)	0.04 (0.03)	0.26 (0.04)	0.11 (0.01)	98.53	3
S-9815-07	46.55 (0.37)	2.7 (0.04)	16.1 (0.34)	12.23 (0.05)	0.17 (0.02)	5.76 (0.18)	9.51 (0.03)	3.84 (0.04)	0.77 (0.02)	0 (0)	0.26 (0)	0.13 (0.02)	98.03	3
S-9815-08	46.58 (0.12)	2.73 (0.02)	16.23 (0.19)	12.43 (0.19)	0.19 (0.03)	5.77 (0.23)	9.54 (0.18)	3.87 (0.07)	0.79 (0.02)	0.02 (0.01)	0.26 (0.02)	0.12 (0.01)	98.54	3
S-9815-09	46.33 (0.21)	3.12 (0.01)	15.73 (0.1)	13.9 (0.27)	0.22 (0.02)	5.2 (0.05)	9.13 (0.07)	4.05 (0.09)	0.71 (0.03)	0.01 (0.02)	0.24 (0.05)	0.13 (0.01)	98.77	3
S-9815-10	46.49 (0.3)	2.71 (0.03)	16.03 (0.31)	12.18 (0.03)	0.19 (0.02)	5.77 (0.14)	9.59 (0.1)	3.78 (0.1)	0.77 (0.02)	0.01 (0.01)	0.26 (0.04)	0.13 (0.02)	97.56	3
**S-9815**	**46.53 (0.21)**	**2.8 (0.14)**	**15.98 (0.21)**	**12.7 (0.51)**	**0.19 (0.02)**	**5.67 (0.24)**	**9.43 (0.15)**	**3.88 (0.09)**	**0.78 (0.03)**	**0.02 (0.01)**	**0.26 (0.01)**	**0.12 (0.01)**	**98.33**	**30**
S-9817-01	45.98 (0.25)	2.86 (0.06)	16.13 (0.32)	12.71 (0.06)	0.18 (0)	5.5 (0.06)	9.14 (0.04)	4.03 (0.11)	0.82 (0.02)	0.03 (0.03)	0.29 (0.02)	0.14 (0.01)	97.79	3
S-9817-02	46.04 (0.45)	2.7 (0.04)	15.99 (0.22)	12.33 (0.12)	0.17 (0.02)	5.83 (0.05)	9.46 (0.17)	3.93 (0.12)	0.75 (0.05)	0.01 (0.02)	0.29 (0.02)	0.12 (0.04)	97.616	3
S-9817-03	46.37 (0.26)	2.69 (0.05)	16.2 (0.12)	12.54 (0.05)	0.18 (0.03)	6 (0.14)	9.49 (0.02)	3.94 (0.23)	0.76 (0.02)	0.03 (0.03)	0.28 (0.02)	0.11 (0.01)	98.60	3
S-9817-04	46.67 (0.13)	2.7 (0.03)	16.31 (0.23)	12.27 (0.05)	0.18 (0.01)	5.93 (0.14)	9.57 (0.03)	3.93 (0.07)	0.76 (0.05)	0.02 (0)	0.29 (0.03)	0.11 (0.03)	98.75	3
S-9817-05	46.49 (0.44)	2.74 (0.04)	16.18 (0.26)	12.41 (0.43)	0.18 (0.01)	5.88 (0.1)	9.56 (0.08)	3.78 (0.1)	0.74 (0.03)	0.01 (0.01)	0.29 (0.01)	0.1 (0.03)	98.35	3
S-9817-06	46.52 (0.13)	2.75 (0.01)	16.2 (0.16)	12.29 (0.39)	0.19 (0.01)	5.85 (0.11)	9.43 (0.08)	3.94 (0.08)	0.77 (0.02)	0.03 (0.02)	0.24 (0.05)	0.11 (0.02)	98.32	3
S-9817-07	46.51 (0.15)	2.89 (0.05)	16.05 (0.22)	12.89 (0.19)	0.18 (0.04)	5.57 (0.21)	9.21 (0.05)	4.01 (0.17)	0.81 (0.03)	0 (0.01)	0.26 (0.01)	0.11 (0.02)	98.49	3
S-9817-08	46.48 (0.22)	2.7 (0.04)	16.23 (0.11)	12.71 (0.09)	0.2 (0.03)	5.78 (0.2)	9.63 (0.08)	3.81 (0.1)	0.75 (0.06)	0.01 (0.01)	0.27 (0.03)	0.1 (0.02)	98.65	3
S-9817-09	46.48 (0.22)	2.74 (0.01)	16.22 (0.09)	12.23 (0.18)	0.19 (0)	5.9 (0.09)	9.56 (0.03)	3.84 (0.12)	0.82 (0.08)	0.02 (0.01)	0.28 (0.01)	0.11 (0.02)	98.39	2
S-9817-10	46.55 (0.14)	3.28 (0.04)	15.36 (0.05)	13.97 (0.12)	0.21 (0.01)	5.06 (0.07)	9.05 (0.04)	4.02 (0.03)	0.76 (0.03)	0 (0)	0.23 (0.02)	0.12 (0.02)	98.62	3
**S-9817**	**46.41 (0.22)**	**2.8 (0.18)**	**16.09 (0.27)**	**12.64 (0.52)**	**0.19 (0.01)**	**5.73 (0.28)**	**9.41 (0.2)**	**3.92 (0.09)**	**0.77 (0.03)**	**0.02 (0.01)**	**0.27 (0.02)**	**0.11 (0.01)**	**98.36**	**29**
S-9816-01	46.56 (0.07)	2.69 (0.04)	16.03 (0.15)	12.51 (0.05)	0.17 (0.05)	6.01 (0.16)	9.73 (0)	3.87 (0.11)	0.74 (0.05)	0.01 (0.02)	0.26 (0.02)	0.09 (0.02)	98.66	3
S-9816-02	46.7 (0.12)	2.6 (0.05)	16.29 (0.3)	12.25 (0.03)	0.18 (0.05)	6.09 (0.12)	9.72 (0.02)	3.89 (0.1)	0.75 (0.03)	0.01 (0)	0.25 (0.02)	0.12 (0.02)	98.84	3
S-9816-03	46.51 (0.07)	2.61 (0.03)	16.29 (0.28)	12.38 (0.25)	0.19 (0.02)	6.24 (0.09)	9.83 (0.04)	3.78 (0.21)	0.73 (0.03)	0.03 (0.01)	0.26 (0.02)	0.14 (0.02)	98.98	3
S-9816-04	46.53 (0.13)	2.61 (0.06)	16.29 (0.11)	11.89 (0.23)	0.18 (0.01)	6.21 (0.16)	9.73 (0.03)	3.78 (0.24)	0.74 (0.03)	0.02 (0.01)	0.23 (0.03)	0.12 (0.01)	98.33	3
S-9816-05	46.46 (0.1)	2.59 (0.01)	16.14 (0.29)	12.41 (0.16)	0.15 (0.02)	6.12 (0.02)	9.65 (0.22)	3.88 (0.2)	0.73 (0.02)	0.02 (0.02)	0.27 (0.02)	0.11 (0.01)	98.53	3
S-9816-06	46.71 (0.14)	2.66 (0.06)	16.1 (0.01)	12.21 (0.25)	0.2 (0.02)	6.15 (0.18)	9.78 (0.03)	3.92 (0.13)	0.78 (0.03)	0.01 (0.01)	0.28 (0.01)	0.11 (0.02)	98.89	3
S-9816-07	46.75 (0.1)	2.61 (0.07)	16.23 (0.19)	12.24 (0.17)	0.17 (0.02)	6.25 (0.27)	9.75 (0.06)	3.82 (0.08)	0.74 (0.05)	0.01 (0.01)	0.27 (0.02)	0.12 (0.01)	98.97	3
S-9816-08	46.79 (0.16)	2.61 (0.02)	16.3 (0.19)	12.27 (0.11)	0.18 (0.01)	6.25 (0.18)	9.74 (0.03)	3.84 (0.16)	0.74 (0)	0.01 (0.01)	0.25 (0.01)	0.11 (0.02)	99.09	3
S-9816-09	46.46 (0.21)	2.55 (0.02)	16.31 (0.16)	11.9 (0.68)	0.19 (0.03)	6.11 (0.21)	9.69 (0.09)	3.77 (0.18)	0.71 (0.03)	0.01 (0.01)	0.26 (0.05)	0.13 (0.02)	98.09	3
S-9816-10	46.6 (0.26)	3.12 (0.08)	15.68 (0.09)	14.01 (0.19)	0.21 (0.02)	5.07 (0.03)	9.05 (0.03)	4.05 (0.15)	0.72 (0.01)	0 (0)	0.23 (0.03)	0.14 (0.02)	98.88	3
**S-9816**	**46.61 (0.12)**	**2.66 (0.17)**	**16.16 (0.2)**	**12.41 (0.6)**	**0.18 (0.02)**	**6.05 (0.35)**	**9.67 (0.22)**	**3.86 (0.08)**	**0.74 (0.02)**	**0.01 (0.01)**	**0.26 (0.02)**	**0.12 (0.01)**	**98.73**	**30**
S-11218-01	46.85 (0.23)	2.54 (0.01)	16.52 (0.15)	11.74 (0.28)	0.17 (0.03)	5.73 (0.19)	9.9 (0.14)	3.82 (0.2)	0.74 (0.01)	0.01 (0.01)	0.27 (0.03)	0.11 (0.01)	98.38	3
S-11218-02	46.46 (0.02)	2.59 (0.06)	16.06 (0.12)	12.15 (0.17)	0.19 (0.01)	5.88 (0.18)	9.78 (0.16)	3.74 (0.1)	0.73 (0.05)	0.03 (0.02)	0.27 (0.01)	0.11 (0.02)	97.97	3
S-11218-03	46.72 (0.03)	2.54 (0.04)	16.3 (0.29)	11.93 (0.08)	0.14 (0.01)	5.84 (0.28)	9.77 (0.2)	3.73 (0.07)	0.73 (0.04)	0.01 (0.01)	0.26 (0.04)	0.11 (0.01)	98.10	3
S-11218-04	46.85 (0.37)	2.51 (0.03)	16.42 (0.28)	11.41 (0.2)	0.15 (0.02)	5.77 (0.26)	9.85 (0.06)	3.76 (0.06)	0.72 (0.03)	0.03 (0)	0.24 (0)	0.14 (0.02)	97.85	3
S-11218-05	46.36 (0.14)	2.55 (0.05)	16.3 (0.18)	11.98 (0.17)	0.17 (0.01)	5.86 (0.18)	9.83 (0.15)	3.77 (0.12)	0.75 (0.05)	0.02 (0.03)	0.27 (0.02)	0.12 (0.03)	97.99	3
S-11218-06	46.72 (0.1)	2.56 (0.07)	16.4 (0.06)	11.89 (0.1)	0.17 (0.04)	5.78 (0.56)	9.89 (0.32)	3.84 (0.04)	0.75 (0.03)	0.02 (0.02)	0.27 (0.02)	0.13 (0.03)	98.39	3
S-11218-07	46.77 (0.41)	2.54 (0.03)	16.32 (0.29)	11.89 (0.08)	0.17 (0.03)	5.93 (0.31)	9.79 (0.15)	3.71 (0.03)	0.76 (0.02)	0 (0.01)	0.29 (0.03)	0.13 (0)	98.29	3
S-11218-08	46.48 (0.2)	2.6 (0.03)	16.15 (0.09)	11.8 (0.37)	0.16 (0.01)	5.81 (0.47)	9.86 (0.15)	3.93 (0.31)	0.75 (0.02)	0.01 (0)	0.23 (0.03)	0.14 (0.02)	97.91	3
S-11218-09	46.64 (0.22)	2.65 (0.05)	16.08 (0.13)	12.26 (0.2)	0.19 (0.04)	5.89 (0.34)	9.76 (0.16)	3.78 (0.01)	0.75 (0.03)	0.01 (0.02)	0.24 (0.04)	0.12 (0.01)	98.36	3
S-11218-10	46.78 (0.2)	2.53 (0.05)	16.27 (0.24)	11.91 (0.09)	0.17 (0.04)	5.81 (0.35)	9.85 (0.11)	3.62 (0.05)	0.77 (0.02)	0.02 (0.02)	0.25 (0.07)	0.15 (0)	98.12	3
**S-11218**	**46.66 (0.17)**	**2.56 (0.04)**	**16.28 (0.15)**	**11.9 (0.23)**	**0.17 (0.01)**	**5.83 (0.06)**	**9.83 (0.05)**	**3.77 (0.08)**	**0.74 (0.02)**	**0.02 (0.01)**	**0.26 (0.02)**	**0.12 (0.01)**	**98.14**	**30**
S-9820-01	47.04 (0.11)	2.47 (0.05)	16.08 (0.09)	12.35 (0.22)	0.19 (0.04)	6.2 (0.19)	10.28 (0)	3.4 (0.08)	0.56 (0.02)	0.02 (0.02)	0.21 (0.02)	0.11 (0.02)	98.89	3
S-9820-02	46.5 (0.13)	2.59 (0.08)	16.42 (0.03)	12.56 (0.25)	0.19 (0.03)	6.15 (0.1)	9.71 (0.08)	3.61 (0.01)	0.63 (0.01)	0.02 (0.02)	0.18 (0.04)	0.1 (0.01)	98.65	3
S-9820-03	46.79 (0.31)	2.67 (0.04)	16.44 (0.14)	12.62 (0.27)	0.18 (0)	5.89 (0.16)	9.74 (0.07)	3.83 (0.08)	0.65 (0.04)	0.01 (0.01)	0.19 (0)	0.07 (0)	99.09	3
S-9820-04	46.95 (0.14)	2.51 (0.04)	16.14 (0.16)	12.24 (0.18)	0.18 (0.02)	6.07 (0.18)	10.26 (0.07)	3.57 (0.05)	0.59 (0.02)	0.03 (0.03)	0.18 (0.02)	0.1 (0.03)	98.83	3
S-9820-05	46.98 (0.04)	2.49 (0.03)	16.03 (0.07)	12.36 (0.18)	0.2 (0.03)	6.26 (0.04)	10.31 (0.1)	3.39 (0.13)	0.58 (0.01)	0.02 (0.02)	0.18 (0.03)	0.12 (0.02)	98.91	3
S-9820-06	47.1 (0.07)	2.45 (0.11)	16.64 (0.22)	12.07 (0.07)	0.19 (0.01)	5.98 (0.04)	9.75 (0.09)	3.76 (0.14)	0.63 (0.03)	0.01 (0.01)	0.23 (0)	0.11 (0.02)	98.92	3
S-9820-07	47.27 (0.2)	2.51 (0.02)	16.06 (0.11)	12.19 (0.38)	0.18 (0.04)	6.19 (0.08)	10.15 (0.11)	3.55 (0.06)	0.57 (0.02)	0.01 (0.01)	0.2 (0.06)	0.13 (0)	99.00	3
S-9820-08	47.23 (0.09)	2.75 (0.05)	15.78 (0.06)	12.94 (0.38)	0.2 (0.02)	5.92 (0.16)	9.33 (0.06)	3.7 (0.01)	0.71 (0.03)	0.02 (0.01)	0.24 (0.06)	0.11 (0.03)	98.94	3
S-9820-09	47.13 (0.15)	2.51 (0.04)	15.99 (0.19)	12.38 (0.17)	0.17 (0.02)	6.32 (0.11)	10.32 (0.08)	3.46 (0.14)	0.56 (0.02)	0.01 (0.01)	0.19 (0.04)	0.11 (0.01)	99.14	3
S-9820-10	46.91 (0.1)	2.54 (0.02)	16.01 (0.09)	12.21 (0.25)	0.17 (0.01)	6.22 (0.11)	10.31 (0.02)	3.51 (0.12)	0.58 (0.02)	0.01 (0.01)	0.2 (0.03)	0.11 (0.02)	98.78	3
**S-9820**	**46.99 (0.23)**	**2.55 (0.1)**	**16.16 (0.26)**	**12.39 (0.26)**	**0.18 (0.01)**	**6.12 (0.15)**	**10.02 (0.35)**	**3.58 (0.15)**	**0.61 (0.05)**	**0.02 (0.01)**	**0.2 (0.02)**	**0.11 (0.01)**	**98.91**	**30**
S-11239-01	46.88 (0.08)	2.6 (0.02)	16.55 (0.15)	12.01 (0.35)	0.18 (0.01)	5.95 (0.23)	9.66 (0.05)	3.75 (0.14)	0.65 (0.02)	0.01 (0.03)	0.17 (0.03)	0.1 (0.02)	98.53	3
S-11239-02	46.66 (0.16)	2.55 (0.02)	16.52 (0.03)	12.33 (0.35)	0.19 (0.03)	5.93 (0.02)	9.62 (0.08)	3.75 (0.09)	0.65 (0.01)	0.02 (0.01)	0.22 (0.01)	0.22 (0.18)	98.67	3
S-11239-03	46.79 (0.09)	2.61 (0.01)	16.52 (0.2)	12.17 (0.13)	0.17 (0.02)	6.12 (0.02)	9.68 (0.03)	3.72 (0.02)	0.63 (0.04)	0.01 (0)	0.25 (0.01)	0.11 (0)	98.77	3
S-11239-04	46.48 (0.14)	2.97 (0.04)	16.24 (0.15)	13.56 (0.12)	0.21 (0.03)	5.65 (0.07)	9.08 (0.03)	3.86 (0.08)	0.72 (0.02)	0.01 (0.01)	0.18 (0.02)	0.11 (0.01)	99.07	3
S-11239-05	46.81 (0.08)	2.54 (0.06)	16.52 (0.16)	12.34 (0.25)	0.19 (0.04)	6.01 (0.08)	9.67 (0.08)	3.65 (0.02)	0.62 (0.02)	0 (0)	0.19 (0.01)	0.11 (0)	98.65	3
S-11239-06	46.75 (0.08)	2.61 (0.04)	16.57 (0.08)	12.14 (0.25)	0.18 (0.01)	5.97 (0.1)	9.71 (0.06)	3.69 (0.09)	0.63 (0.03)	0.01 (0.01)	0.2 (0.03)	0.09 (0.02)	98.56	3
S-11239-07	46.65 (0.11)	2.67 (0.04)	16.38 (0.02)	12.57 (0.2)	0.17 (0.03)	6.09 (0.04)	9.62 (0.04)	3.7 (0.09)	0.63 (0.02)	0.03 (0.03)	0.19 (0.02)	0.1 (0.02)	98.80	3
S-11239-08	46.93 (0.06)	2.52 (0.08)	16.5 (0.06)	12.58 (0.01)	0.18 (0.04)	6.06 (0.07)	9.68 (0.03)	3.72 (0.08)	0.63 (0.01)	0.02 (0.01)	0.22 (0.03)	0.11 (0.02)	99.14	3
S-11239-09	46.88 (0.07)	2.56 (0.03)	16.61 (0.05)	12.34 (0.06)	0.21 (0.02)	6.04 (0.06)	9.65 (0.04)	3.65 (0.09)	0.64 (0.03)	0.02 (0)	0.2 (0.05)	0.12 (0.02)	98.93	3
S-11239-10	46.79 (0.19)	2.66 (0.04)	16.25 (0.1)	12.78 (0.18)	0.17 (0.04)	6.12 (0.06)	9.66 (0.06)	3.74 (0.16)	0.63 (0.02)	0.01 (0)	0.22 (0.01)	0.11 (0.01)	99.12	3
**S-11239**	**46.76 (0.13)**	**2.63 (0.13)**	**16.47 (0.13)**	**12.48 (0.44)**	**0.18 (0.02)**	**5.99 (0.14)**	**9.6 (0.19)**	**3.72 (0.06)**	**0.64 (0.03)**	**0.01 (0.01)**	**0.2 (0.02)**	**0.12 (0.04)**	**98.82**	**30**

EPMA No.	LA-ICPMS Sc	V	Co	Ni	Cu	Zn	Ga	Ge	Rb	Sr	Y	Zr	Nb	Ba	La	Ce	Pr	Nd	Sm	Eu	Gd	Tb	Dy	Ho	Er	Tm	Yb	Lu	Hf	Ta	Pb	Th	U
S-9809-01	29.59 (1.01)	296.46 (6.19)	41.39 (1.14)	41.14 (2.11)	56.99 (2.13)	106.93 (4.8)	24.73 (0.92)		15.45 (0.45)	335.78 (5.72)	39.25 (0.78)	215.22 (4)	26.18 (0.53)	178.06 (4.32)	20.78 (0.54)	47.4 (0.9)	6.44 (0.15)	29.12 (0.85)	6.68 (0.39)	2.14 (0.11)	7.56 (0.48)	1.12 (0.06)	7.28 (0.32)	1.34 (0.07)	3.85 (0.18)	0.6 (0.04)	3.7 (0.3)	0.5 (0.05)	5.18 (0.23)	1.53 (0.05)	1.6 (0.13)	1.88 (0.13)	0.53 (0.04)
S-9809-04	30.77 (0.8)	319.87 (5.37)	40.69 (0.95)	35.24 (1.41)	62.22 (1.6)	104.52 (3.46)	23.55 (0.65)	1.57 (0.39)	16.86 (0.45)	327.83 (4.67)	41.11 (0.64)	231.32 (3.36)	27.82 (0.44)	190.4 (4.12)	21.66 (0.45)	51.98 (0.88)	6.93 (0.17)	32.52 (0.84)	7.71 (0.37)	2.43 (0.1)	7.73 (0.36)	1.23 (0.06)	7.73 (0.24)	1.53 (0.06)	4.28 (0.16)	0.61 (0.03)	3.77 (0.19)	0.59 (0.03)	5.49 (0.25)	1.77 (0.05)	1.8 (0.1)	1.8 (0.07)	0.56 (0.04)
S-9809-09	26.76 (0.78)	286.92 (4.72)	43.71 (0.87)	53.46 (1.7)	59.34 (1.63)	113.25 (4.33)	25.25 (0.7)	2.1 (0.49)	15.49 (0.42)	352.37 (5.09)	36.04 (0.59)	206.29 (3.22)	25.14 (0.39)	175.15 (3.42)	19 (0.38)	46.96 (0.8)	6.32 (0.17)	28.15 (0.89)	7.09 (0.42)	2.49 (0.11)	7.39 (0.39)	1.14 (0.05)	6.95 (0.29)	1.35 (0.07)	3.84 (0.14)	0.51 (0.04)	3.29 (0.19)	0.5 (0.04)	4.79 (0.22)	1.57 (0.05)	2.04 (0.12)	1.67 (0.09)	0.53 (0.04)
**S-9809**	**29.04 (1.87)**	**301.08 (16.51)**	**41.93 (1.56)**	**43.28 (8.93)**	**59.52 (2.35)**	**108.24 (5.65)**	**24.51 (0.85)**		**15.93 (0.83)**	**338.66 (11.63)**	**38.8 (2.3)**	**217.61 (11.52)**	**26.38 (0.99)**	**181.2 (7.02)**	**20.48 (1.26)**	**48.78 (2.48)**	**6.56 (0.29)**	**29.93 (2.09)**	**7.16 (0.48)**	**2.35 (0.16)**	**7.56 (0.15)**	**1.16 (0.05)**	**7.32 (0.36)**	**1.4 (0.1)**	**3.99 (0.22)**	**0.57 (0.05)**	**3.59 (0.25)**	**0.53 (0.05)**	**5.15 (0.33)**	**1.63 (0.09)**	**1.81 (0.22)**	**1.78 (0.1)**	**0.54 (0.01)**
S-9810-02	26.33 (0.66)	291.07 (4.5)	41.66 (0.8)	49.99 (1.52)	57.65 (1.49)	100.13 (3.82)	24.68 (0.63)		15.07 (0.48)	367.67 (5.27)	38.16 (0.66)	212.58 (3.14)	25.71 (0.35)	177.71 (3.53)	20.13 (0.42)	47.67 (0.75)	6.52 (0.13)	28.56 (0.77)	6.99 (0.43)	2.46 (0.11)	7.3 (0.33)	1.16 (0.05)	6.97 (0.27)	1.45 (0.07)	3.55 (0.17)	0.56 (0.04)	3.87 (0.21)	0.58 (0.04)	4.91 (0.23)	1.64 (0.05)	1.94 (0.1)	1.59 (0.08)	0.52 (0.04)
S-9810-08	26.04 (1.03)	281.85 (6.59)	42.54 (1.37)	52.34 (2.85)	64.82 (3.43)	108.03 (4.78)	23.8 (1.23)		15.95 (0.66)	343.42 (7.43)	36.55 (0.86)	198.77 (3.93)	25.2 (0.63)	170.58 (5.82)	18.48 (0.56)	45 (1.16)	5.78 (0.23)	25.6 (0.96)	7.12 (0.49)	2.2 (0.14)	6.42 (0.52)	0.86 (0.06)	6.6 (0.36)	1.14 (0.07)	3.03 (0.2)	0.6 (0.05)	2.84 (0.35)	0.41 (0.05)	4.84 (0.43)	1.49 (0.05)	1.62 (0.14)	1.55 (0.11)	0.49 (0.04)
S-9810-09	30.05 (1.24)	301.9 (5.69)	41.08 (1.66)	43.32 (2.28)	60.62 (1.85)	97.99 (4.65)	25.42 (0.8)		16.38 (0.77)	338.68 (6.47)	40.64 (0.83)	225.43 (4.28)	25.73 (0.57)	186.41 (4.64)	20.16 (0.54)	50.03 (0.97)	6.35 (0.23)	29.49 (0.96)	7.13 (0.49)	2.34 (0.17)	7.67 (0.39)	1.17 (0.06)	6.95 (0.33)	1.38 (0.09)	3.91 (0.17)	0.51 (0.05)	3.58 (0.21)	0.52 (0.04)	5.13 (0.36)	1.76 (0.05)	2.04 (0.14)	1.78 (0.11)	0.58 (0.05)
**S-9810**	**27.47 (2.03)**	**291.6 (9.77)**	**41.76 (0.73)**	**48.55 (4.49)**	**61.03 (3.23)**	**102.05 (6.63)**	**24.63 (0.79)**		**15.8 (0.69)**	**349.92 (14.44)**	**38.45 (1.85)**	**212.26 (12.1)**	**25.54 (0.22)**	**178.24 (6.88)**	**19.59 (0.89)**	**47.57 (2.25)**	**6.22 (0.34)**	**27.88 (1.85)**	**7.08 (0.07)**	**2.33 (0.11)**	**7.13 (0.57)**	**1.06 (0.16)**	**6.84 (0.19)**	**1.32 (0.16)**	**3.5 (0.4)**	**0.55 (0.04)**	**3.43 (0.51)**	**0.5 (0.08)**	**4.96 (0.14)**	**1.63 (0.09)**	**1.87 (0.22)**	**1.64 (0.12)**	**0.53 (0.04)**
S-11236-03	29.74 (0.92)	315.58 (5.44)	41.19 (1.01)	38.28 (1.79)	68.68 (1.85)	109.56 (4.05)	25.59 (0.82)		16.91 (0.45)	321.5 (4.97)	40.57 (0.79)	225.44 (3.68)	27.32 (0.46)	191.78 (4.59)	20.83 (0.44)	51.16 (0.85)	6.89 (0.19)	32.29 (0.7)	8.2 (0.46)	2.21 (0.11)	7.27 (0.41)	1.27 (0.07)	8.21 (0.28)	1.61 (0.07)	4.28 (0.18)	0.59 (0.03)	3.66 (0.23)	0.57 (0.04)	5.8 (0.27)	1.79 (0.05)	2.26 (0.13)	1.79 (0.08)	0.61 (0.06)
S-11236-09	28.74 (0.81)	294.79 (4.93)	43.24 (0.99)	48.48 (2.12)	60.09 (1.59)	108.08 (4.71)	24.04 (0.66)		15.25 (0.39)	354.94 (5.44)	38.29 (0.68)	217.5 (3.36)	25.79 (0.45)	180.71 (3.8)	20.82 (0.37)	47.97 (0.79)	6.76 (0.22)	28.41 (0.85)	7.76 (0.39)	2.46 (0.11)	6.69 (0.3)	1.13 (0.05)	7.65 (0.33)	1.41 (0.06)	4.05 (0.18)	0.66 (0.04)	3.22 (0.18)	0.52 (0.04)	5.55 (0.26)	1.61 (0.05)	2.84 (0.15)	1.66 (0.08)	0.51 (0.04)
S-11236-10	27.76 (0.84)	296.63 (5.09)	41.47 (0.98)	45.9 (1.65)	61.39 (2.24)	95.74 (3.18)	23.19 (0.64)	1.45 (0.5)	15.78 (0.48)	338.74 (5.02)	37.45 (0.66)	213.75 (2.98)	26.01 (0.47)	183.55 (3.86)	19.94 (0.41)	47.87 (0.69)	6.35 (0.15)	29.31 (0.93)	7.48 (0.42)	2.38 (0.11)	7.36 (0.35)	1.17 (0.05)	7.41 (0.28)	1.52 (0.07)	4.24 (0.16)	0.61 (0.04)	3.77 (0.22)	0.53 (0.03)	6 (0.27)	1.82 (0.05)	1.94 (0.1)	1.75 (0.07)	0.5 (0.03)
**S-11236**	**28.75 (0.9)**	**302.33 (11.21)**	**41.97 (1.1)**	**44.22 (5.09)**	**63.39 (4.15)**	**104.46 (9.51)**	**24.28 (1.19)**		**15.98 (0.87)**	**338.39 (15.53)**	**38.77 (1.45)**	**218.9 (5.42)**	**26.37 (0.61)**	**185.35 (4.99)**	**20.53 (0.48)**	**49 (1.67)**	**6.66 (0.25)**	**30 (1.85)**	**7.81 (0.34)**	**2.35 (0.11)**	**7.11 (0.32)**	**1.19 (0.07)**	**7.75 (0.38)**	**1.51 (0.1)**	**4.19 (0.11)**	**0.62 (0.03)**	**3.55 (0.28)**	**0.54 (0.02)**	**5.78 (0.21)**	**1.74 (0.08)**	**2.35 (0.46)**	**1.73 (0.06)**	**0.54 (0.06)**
	S1G-Sc	S1G-V	S1G-Co		S1G-Cu	S1G-Zn	S1G-Ga		S1G-Rb	S1G-Sr	S1G-Y	S1G-Zr	S1G-Nb	S1G-Ba	S1G-La	S1G-Ce	S1G-Pr	S1G-Nd	S1G-Sm	S1G-Eu	S1G-Gd	S1G-Tb	S1G-Dy	S1G-Ho	S1G-Er	S1G-Tm	S1G-Yb	S1G-Lu	S1G-Hf	S1G-Ta	S1G-Pb	S1G-Th	S1G-U
S-9811-03	30.37 (0.72)	311.08 (4.77)	42.82 (0.89)	45.99 (1.52)	67.91 (1.98)	112.14 (4.47)	26.4 (0.72)	1.25 (0.46)	17.16 (0.43)	331.62 (4.62)	40.49 (0.76)	230.78 (3.41)	27.71 (0.41)	185.23 (3.85)	21.54 (0.46)	52.07 (0.82)	6.8 (0.15)	30.52 (0.84)	7.73 (0.43)	2.67 (0.12)	8.1 (0.36)	1.27 (0.06)	7.88 (0.31)	1.58 (0.07)	4.05 (0.16)	0.65 (0.04)	3.77 (0.19)	0.56 (0.04)	5.6 (0.27)	1.77 (0.04)	1.94 (0.11)	1.7 (0.07)	0.61 (0.05)
S-9811-04	31.01 (0.79)	306.18 (5.82)	40.19 (1.04)	35.33 (1.77)	58.96 (1.99)	94.12 (3.5)	24.46 (0.68)	1.66 (0.58)	15.5 (0.55)	331.63 (4.92)	39.39 (0.77)	223.67 (3.86)	26.88 (0.45)	188.84 (3.88)	20.73 (0.44)	51.11 (0.87)	6.71 (0.15)	31 (0.78)	8.04 (0.41)	2.48 (0.11)	7.79 (0.38)	1.27 (0.06)	7.8 (0.29)	1.56 (0.06)	4.44 (0.15)	0.6 (0.04)	3.51 (0.21)	0.57 (0.04)	5.4 (0.25)	1.89 (0.06)	1.92 (0.11)	1.9 (0.09)	0.56 (0.04)
S-9811-09	26.87 (0.74)	280.02 (4.87)	40.91 (1.03)	50.5 (2.24)	59.58 (2.04)	99.09 (3.65)	23.7 (0.74)	2.04 (0.64)	15.1 (0.49)	343.26 (5.47)	36.43 (0.73)	204.69 (3.37)	24.85 (0.44)	174.67 (3.63)	18.92 (0.36)	46.63 (0.76)	6.19 (0.14)	29.33 (0.81)	6.08 (0.33)	2.25 (0.1)	7.85 (0.36)	1.29 (0.06)	6.99 (0.28)	1.42 (0.06)	3.71 (0.16)	0.56 (0.04)	3.68 (0.19)	0.52 (0.03)	4.72 (0.24)	1.55 (0.05)	1.79 (0.12)	1.67 (0.07)	0.52 (0.04)
**S-9811**	**29.42 (2.02)**	**299.09 (16.26)**	**41.3 (1.35)**	**43.94 (7.48)**	**62.15 (4.47)**	**101.78 (11.67)**	**24.85 (1.36)**		**15.92 (1.12)**	**335.51 (6.24)**	**38.77 (1.88)**	**219.71 (12.24)**	**26.48 (1.08)**	**182.92 (6.39)**	**20.4 (1.25)**	**49.93 (2.59)**	**6.57 (0.29)**	**30.28 (0.78)**	**7.28 (0.98)**	**2.47 (0.19)**	**7.91 (0.15)**	**1.28 (0.01)**	**7.55 (0.46)**	**1.52 (0.08)**	**4.07 (0.33)**	**0.6 (0.04)**	**3.65 (0.13)**	**0.55 (0.03)**	**5.24 (0.43)**	**1.74 (0.12)**	**1.88 (0.08)**	**1.76 (0.12)**	**0.56 (0.04)**
S-801-02	26.86 (0.75)	282.82 (4.44)	41.36 (0.78)	51.22 (1.69)	53.11 (1.42)	107.7 (12.98)	25.19 (0.62)	1.51 (0.45)	16.47 (0.43)	372.53 (5.14)	38.49 (0.65)	216.38 (3.09)	26.46 (0.37)	180.22 (3.54)	20.26 (0.36)	47.93 (0.73)	6.54 (0.14)	30.21 (0.76)	7.12 (0.34)	2.43 (0.09)	8.26 (0.32)	1.23 (0.06)	7.41 (0.3)	1.41 (0.06)	4.34 (0.17)	0.56 (0.03)	3.4 (0.19)	0.53 (0.03)	5.07 (0.24)	1.63 (0.04)	2.07 (0.11)	1.73 (0.07)	0.55 (0.04)
S-801-03	26.31 (0.68)	278.95 (4.82)	41.43 (0.95)	54.98 (2.24)	32.02 (1.09)	93.23 (3.42)	23.07 (0.63)		15.48 (0.46)	355.83 (5.4)	36.42 (0.66)	201.15 (3)	24.49 (0.41)	176.62 (3.43)	18.71 (0.35)	45.26 (0.7)	6.01 (0.14)	26.45 (0.71)	7.08 (0.35)	2.11 (0.09)	6.81 (0.36)	1.07 (0.05)	6.79 (0.26)	1.5 (0.06)	3.92 (0.18)	0.53 (0.04)	3.68 (0.2)	0.44 (0.04)	5.27 (0.23)	1.56 (0.04)	1.73 (0.1)	1.59 (0.07)	0.55 (0.04)
S-801-06	26.78 (0.68)	283.92 (4.81)	43.49 (1.05)	51.64 (2.36)	60.37 (2.05)	103.16 (4.02)	24.35 (0.77)	1.67 (0.69)	15.8 (0.45)	345.74 (5.45)	36.61 (0.61)	203.81 (3.25)	24.7 (0.43)	176.7 (3.41)	19.24 (0.42)	45.62 (0.7)	6.15 (0.15)	28.73 (0.79)	5.9 (0.33)	2.25 (0.1)	7.1 (0.33)	1.08 (0.05)	6.92 (0.3)	1.23 (0.06)	3.75 (0.16)	0.51 (0.04)	3.88 (0.21)	0.47 (0.03)	5.51 (0.27)	1.66 (0.05)	1.77 (0.11)	1.59 (0.06)	0.49 (0.04)
**S-801**	**26.65 (0.27)**	**281.9 (2.54)**	**42.09 (1.2)**	**52.61 (1.98)**	**48.5 (13.19)**	**101.36 (9.28)**	**24.2 (1.04)**		**15.91 (0.52)**	**358.03 (12.56)**	**37.17 (1.03)**	**207.11 (7.38)**	**25.22 (0.79)**	**177.85 (1.79)**	**19.4 (0.73)**	**46.27 (1.29)**	**6.24 (0.24)**	**28.46 (1.72)**	**6.7 (0.64)**	**2.26 (0.14)**	**7.39 (0.68)**	**1.13 (0.08)**	**7.04 (0.3)**	**1.38 (0.13)**	**4 (0.27)**	**0.53 (0.02)**	**3.65 (0.23)**	**0.48 (0.04)**	**5.28 (0.2)**	**1.62 (0.03)**	**1.86 (0.18)**	**1.64 (0.08)**	**0.53 (0.04)**
S-9812-05	28.13 (0.73)	276.65 (4.96)	42.76 (1)	56.25 (1.82)	58.97 (1.7)	93.84 (3.51)	23.73 (0.71)	1.38 (0.58)	14.42 (0.45)	346.48 (5.27)	37.26 (0.62)	205.59 (2.98)	25.43 (0.39)	178.83 (3.72)	19.89 (0.37)	47.2 (0.79)	6.1 (0.16)	26.75 (0.78)	6.81 (0.35)	2.3 (0.09)	7.59 (0.38)	1.17 (0.06)	7.13 (0.28)	1.37 (0.06)	3.63 (0.15)	0.59 (0.03)	3.04 (0.19)	0.52 (0.03)	5.32 (0.24)	1.66 (0.05)	1.67 (0.11)	1.77 (0.08)	0.52 (0.04)
S-9812-09	27.37 (0.74)	277.14 (4.41)	41.84 (0.87)	55.73 (1.84)	59.9 (1.99)	97.46 (3.39)	24.32 (0.74)	1.41 (0.52)	15.27 (0.48)	341.11 (5.01)	36.46 (0.6)	205.92 (2.86)	25.3 (0.37)	176.78 (3.47)	19.33 (0.33)	47.44 (0.72)	6.14 (0.14)	28.47 (0.82)	6.93 (0.41)	2.38 (0.11)	7.48 (0.35)	1.17 (0.05)	7.15 (0.31)	1.39 (0.06)	3.95 (0.16)	0.59 (0.03)	3.51 (0.21)	0.48 (0.03)	4.91 (0.21)	1.63 (0.05)	1.82 (0.11)	1.68 (0.07)	0.49 (0.04)
S-9812-10	27.44 (0.71)	278.88 (4.12)	41.15 (0.82)	57.48 (1.67)	58.26 (1.49)	96.4 (3.85)	23.42 (0.58)	1.66 (0.44)	14.01 (0.39)	368.7 (4.98)	37.55 (0.59)	212.89 (3.08)	25.87 (0.37)	175.75 (3.55)	20.26 (0.36)	47.15 (0.71)	6.18 (0.16)	28.06 (0.72)	6.99 (0.34)	2.34 (0.12)	7.81 (0.34)	1.22 (0.05)	7.51 (0.28)	1.4 (0.05)	4.04 (0.17)	0.53 (0.03)	3.5 (0.19)	0.52 (0.04)	5.1 (0.23)	1.59 (0.04)	1.7 (0.12)	1.8 (0.08)	0.55 (0.04)
**S-9812**	**27.65 (0.38)**	**277.56 (1.14)**	**41.92 (0.8)**	**56.48 (0.86)**	**59.04 (0.74)**	**95.9 (2.34)**	**23.83 (0.45)**		**14.57 (0.66)**	**352.1 (13.58)**	**37.09 (0.51)**	**208.13 (3.74)**	**25.53 (0.22)**	**177.12 (1.36)**	**19.83 (0.43)**	**47.27 (0.14)**	**6.14 (0.04)**	**27.76 (0.81)**	**6.91 (0.08)**	**2.34 (0.04)**	**7.63 (0.15)**	**1.19 (0.02)**	**7.26 (0.2)**	**1.39 (0.01)**	**3.87 (0.19)**	**0.57 (0.03)**	**3.35 (0.26)**	**0.51 (0.02)**	**5.11 (0.19)**	**1.63 (0.02)**	**1.73 (0.08)**	**1.75 (0.06)**	**0.52 (0.03)**
S-9813-05	28.21 (0.68)	277.88 (4.23)	41.77 (0.83)	54.83 (1.9)	55.16 (1.43)	92.74 (3.77)	24.02 (0.64)	1.57 (0.43)	15.35 (0.37)	372.4 (5)	37.58 (0.64)	211.2 (3.16)	26.17 (0.39)	174.03 (3.1)	19.91 (0.37)	46.68 (0.71)	6.44 (0.15)	28.89 (0.77)	7.03 (0.33)	2.44 (0.1)	7.37 (0.37)	1.15 (0.05)	7.22 (0.23)	1.4 (0.06)	3.96 (0.17)	0.58 (0.04)	4.05 (0.22)	0.51 (0.03)	5.53 (0.19)	1.56 (0.04)	1.75 (0.12)	1.64 (0.07)	0.51 (0.04)
S-9813-07	24.96 (0.63)	265.99 (4.53)	40.45 (0.89)	56.42 (1.95)	57.03 (1.72)	101.69 (14.08)	23.89 (0.6)		15.09 (0.5)	371.19 (5.44)	33.72 (0.55)	194.1 (2.99)	23.74 (0.44)	168.83 (3.32)	18.17 (0.38)	45.23 (0.82)	6.26 (0.15)	26.94 (0.75)	7.02 (0.41)	2.25 (0.08)	7.1 (0.32)	1.09 (0.06)	6.93 (0.24)	1.21 (0.06)	3.39 (0.16)	0.49 (0.03)	3.07 (0.19)	0.5 (0.03)	5.07 (0.23)	1.6 (0.05)	1.61 (0.1)	1.42 (0.07)	0.48 (0.04)
S-9813-08	28.57 (0.78)	270.2 (4.57)	41.45 (0.98)	58.51 (1.99)	58.35 (1.93)	96.25 (3.42)	24.82 (0.78)	1.2 (0.48)	15.68 (0.44)	348.54 (4.97)	35.08 (0.57)	202.3 (3.13)	24.33 (0.41)	174.09 (3.22)	19.23 (0.42)	45.84 (0.68)	6.39 (0.16)	27.82 (0.64)	7.18 (0.4)	2.26 (0.1)	7.45 (0.38)	1.21 (0.06)	6.92 (0.26)	1.35 (0.05)	3.78 (0.16)	0.6 (0.03)	3.12 (0.17)	0.56 (0.04)	4.59 (0.25)	1.63 (0.04)	2.01 (0.1)	1.55 (0.08)	0.56 (0.04)
**S-9813**	**27.25 (1.8)**	**271.36 (5.87)**	**41.22 (0.68)**	**56.59 (1.78)**	**56.85 (1.43)**	**96.9 (5.65)**	**24.24 (0.5)**		**15.37 (0.31)**	**364.04 (12.48)**	**35.46 (1.75)**	**202.53 (7.77)**	**24.75 (0.93)**	**172.32 (2.62)**	**19.1 (0.82)**	**45.92 (0.65)**	**6.36 (0.08)**	**27.89 (0.89)**	**7.08 (0.08)**	**2.31 (0.1)**	**7.31 (0.16)**	**1.15 (0.06)**	**7.02 (0.16)**	**1.32 (0.09)**	**3.71 (0.26)**	**0.56 (0.05)**	**3.42 (0.53)**	**0.52 (0.03)**	**5.06 (0.44)**	**1.6 (0.02)**	**1.79 (0.2)**	**1.54 (0.11)**	**0.51 (0.04)**
S-9814-01	27.94 (0.92)	272.56 (4.63)	43.33 (1)	61.64 (2.16)	59.33 (1.85)	90.97 (3.65)	23.87 (0.82)		15.07 (0.49)	361.22 (5.2)	34.32 (0.57)	198.06 (2.97)	23.9 (0.45)	175.17 (3.85)	17.94 (0.38)	45.26 (0.81)	5.71 (0.15)	26.99 (0.85)	6.82 (0.36)	2.38 (0.12)	7.53 (0.38)	1.09 (0.05)	7.44 (0.27)	1.29 (0.06)	3.77 (0.17)	0.59 (0.03)	3.5 (0.21)	0.44 (0.03)	5.06 (0.25)	1.61 (0.05)	1.7 (0.1)	1.62 (0.09)	0.48 (0.04)
S-9814-07	27.01 (0.81)	259.91 (5.02)	41.87 (1.12)	59.4 (2.52)	56.82 (3.17)	99.22 (3.6)	24.29 (0.89)		15.44 (0.44)	360.88 (6.19)	34.2 (0.61)	192.39 (3.12)	23.36 (0.44)	164.53 (3.63)	18.57 (0.42)	45.14 (0.79)	5.65 (0.16)	28.27 (0.88)	6.92 (0.4)	2.12 (0.12)	7.01 (0.29)	0.99 (0.05)	6.14 (0.29)	1.2 (0.06)	3.55 (0.16)	0.5 (0.04)	3.12 (0.19)	0.43 (0.04)	4.79 (0.24)	1.6 (0.05)	2.01 (0.14)	1.63 (0.09)	0.5 (0.03)
**S-9814**	**27.48 (0.6)**	**266.23 (8.71)**	**42.6 (1.02)**	**60.52 (1.52)**	**58.07 (1.59)**	**95.1 (7.31)**	**24.08 (0.29)**		**15.25 (0.27)**	**361.05 (0.23)**	**34.26 (0.08)**	**195.22 (3.64)**	**23.63 (0.28)**	**169.85 (6.53)**	**18.26 (0.42)**	**45.2 (0.08)**	**5.68 (0.04)**	**27.63 (0.83)**	**6.87 (0.07)**	**2.25 (0.16)**	**7.27 (0.33)**	**1.04 (0.07)**	**6.79 (0.86)**	**1.24 (0.06)**	**3.66 (0.14)**	**0.54 (0.06)**	**3.31 (0.26)**	**0.43 (0)**	**4.92 (0.18)**	**1.61 (0)**	**1.86 (0.22)**	**1.63 (0.01)**	**0.49 (0.01)**
S-9815-01	29.33 (0.74)	283.06 (4.33)	42.67 (0.92)	56.23 (2.01)	61.2 (1.64)	95.77 (3.64)	23.17 (0.6)		14.6 (0.42)	352.4 (4.71)	37.64 (0.64)	214.42 (3.22)	25.24 (0.41)	180.04 (3.5)	19.64 (0.4)	46.55 (0.73)	6.12 (0.14)	30.25 (0.7)	7.08 (0.34)	2.41 (0.1)	8.1 (0.45)	1.15 (0.07)	6.73 (0.2)	1.37 (0.05)	4.15 (0.18)	0.5 (0.03)	3.82 (0.2)	0.55 (0.03)	5.18 (0.24)	1.72 (0.04)	1.68 (0.11)	1.48 (0.07)	0.6 (0.04)
S-9815-08	29.51 (0.83)	271.45 (4.79)	42.58 (1.02)	59.18 (1.97)	58.55 (1.99)	98.34 (4.95)	23.29 (0.64)	1.47 (0.5)	14.17 (0.41)	369.07 (5.78)	35.63 (0.62)	201.38 (3.36)	24.37 (0.43)	174.58 (3.55)	19.05 (0.38)	44.95 (0.74)	6.26 (0.14)	29.4 (0.82)	7.32 (0.36)	2.11 (0.09)	7.43 (0.33)	1.14 (0.05)	6.56 (0.25)	1.29 (0.06)	3.57 (0.15)	0.47 (0.03)	3.32 (0.2)	0.52 (0.03)	4.83 (0.25)	1.6 (0.05)	1.89 (0.1)	1.42 (0.06)	0.45 (0.03)
S-9815-10	26.67 (0.87)	249.96 (5.89)	38.81 (1.11)	59.03 (2.67)	51.96 (1.92)	84.47 (3.36)	23.94 (0.7)	1.6 (0.59)	13.04 (0.53)	405.64 (7.41)	31.32 (0.84)	181.87 (4.66)	21.7 (0.53)	163.92 (3.61)	17.18 (0.45)	41.21 (1.01)	5.85 (0.17)	25.44 (0.86)	5.6 (0.35)	2.2 (0.1)	6.73 (0.32)	1.01 (0.05)	6.27 (0.31)	1.22 (0.06)	3.5 (0.18)	0.48 (0.03)	2.98 (0.18)	0.48 (0.03)	4.06 (0.25)	1.39 (0.05)	1.42 (0.09)	1.38 (0.08)	0.42 (0.04)
**S-9815**	**28.5 (1.44)**	**268.16 (16.35)**	**41.35 (2.18)**	**58.15 (1.6)**	**57.24 (4.26)**	**92.86 (9.25)**	**23.47 (0.41)**		**13.94 (0.83)**	**375.7 (25.29)**	**34.86 (2.9)**	**199.22 (14.87)**	**23.77 (1.35)**	**172.85 (7.12)**	**18.62 (1.2)**	**44.23 (2.45)**	**6.08 (0.18)**	**28.36 (2.34)**	**6.67 (0.86)**	**2.24 (0.14)**	**7.42 (0.61)**	**1.1 (0.07)**	**6.52 (0.22)**	**1.29 (0.07)**	**3.74 (0.32)**	**0.48 (0.01)**	**3.38 (0.41)**	**0.52 (0.03)**	**4.69 (0.54)**	**1.57 (0.11)**	**1.67 (0.24)**	**1.43 (0.05)**	**0.49 (0.1)**
S-9817-03	25.97 (0.75)	270.33 (4.69)	43.93 (1.07)	57.4 (2.11)	60.44 (2.22)	90.35 (2.82)	23.52 (0.82)	2.14 (0.51)	15.12 (0.42)	358.14 (5.21)	36.28 (0.62)	195.89 (2.8)	24.32 (0.41)	172.95 (3.09)	18.48 (0.34)	45.09 (0.7)	6.07 (0.14)	27.49 (0.75)	6.68 (0.36)	2.24 (0.1)	7.34 (0.31)	1.1 (0.05)	6.94 (0.29)	1.23 (0.06)	3.93 (0.14)	0.5 (0.03)	3.53 (0.17)	0.47 (0.03)	4.81 (0.2)	1.58 (0.05)	1.37 (0.08)	1.64 (0.07)	0.53 (0.04)
S-9817-04	28.27 (0.83)	268.09 (4.5)	41.42 (1)	62.24 (2.25)	61.72 (1.95)	93.81 (3.75)	24.25 (0.73)	1.93 (0.54)	14.42 (0.41)	360.08 (5.01)	35.34 (0.56)	198.29 (3.03)	24.69 (0.38)	161.7 (3.2)	18.4 (0.32)	43.17 (0.69)	5.83 (0.13)	26.87 (0.7)	5.67 (0.3)	2.16 (0.11)	7.22 (0.33)	1.04 (0.05)	6.79 (0.28)	1.29 (0.05)	3.59 (0.17)	0.49 (0.03)	3.31 (0.21)	0.46 (0.03)	4.92 (0.23)	1.46 (0.04)	1.66 (0.1)	1.49 (0.06)	0.53 (0.04)
S-9817-10	29.76 (0.74)	316.82 (5.02)	40.93 (0.82)	31.57 (1.15)	66.44 (1.82)	111.98 (10.42)	24.46 (0.64)		17.13 (0.45)	335.82 (4.33)	44.37 (0.73)	245.62 (3.67)	29.94 (0.41)	198.51 (3.93)	22.85 (0.44)	54.51 (0.79)	7.51 (0.17)	33.89 (0.81)	8.87 (0.39)	2.6 (0.12)	8.87 (0.39)	1.37 (0.06)	8.26 (0.26)	1.62 (0.07)	4.67 (0.19)	0.73 (0.04)	3.82 (0.25)	0.64 (0.03)	5.97 (0.25)	1.79 (0.05)	1.94 (0.11)	1.93 (0.09)	0.58 (0.04)
**S-9817**	**28 (1.73)**	**285.08 (26.78)**	**42.09 (1.6)**	**50.4 (15.84)**	**62.87 (2.83)**	**98.71 (14.57)**	**24.08 (0.48)**		**15.55 (1.45)**	**351.34 (12.52)**	**38.66 (4.45)**	**213.27 (25.46)**	**26.32 (2.3)**	**177.72 (16.36)**	**19.91 (2.37)**	**47.59 (5.41)**	**6.47 (0.8)**	**29.42 (3.54)**	**7.07 (1.52)**	**2.33 (0.21)**	**7.81 (0.82)**	**1.17 (0.17)**	**7.33 (0.75)**	**1.38 (0.2)**	**4.06 (0.5)**	**0.57 (0.12)**	**3.56 (0.25)**	**0.52 (0.09)**	**5.23 (0.59)**	**1.61 (0.11)**	**1.66 (0.28)**	**1.69 (0.22)**	**0.54 (0.03)**
S-9816-04	28.22 (0.74)	269.83 (4.11)	41.72 (0.79)	71.62 (2.1)	59.22 (1.77)	87.16 (3.6)	22.53 (0.57)		13.6 (0.33)	362.92 (4.84)	34.14 (0.52)	193.75 (2.72)	23.17 (0.39)	160.91 (3.13)	18.05 (0.34)	42.9 (0.66)	5.91 (0.13)	26.17 (0.71)	6.57 (0.34)	2.14 (0.09)	6.77 (0.29)	1 (0.04)	6.85 (0.26)	1.36 (0.07)	3.37 (0.13)	0.47 (0.03)	3.28 (0.17)	0.46 (0.03)	4.51 (0.24)	1.5 (0.04)	1.55 (0.09)	1.5 (0.06)	0.45 (0.04)
S-9816-07	27.7 (0.77)	272.59 (4.56)	43.68 (1.09)	69.08 (2.33)	58.86 (1.81)	145.41 (42.65)	22.78 (0.72)	1.5 (0.57)	14.46 (0.44)	361.51 (4.95)	35.22 (0.6)	190.14 (2.87)	23.44 (0.42)	165.34 (3.75)	17.21 (0.4)	41.67 (0.66)	5.87 (0.15)	24.66 (0.68)	6.58 (0.38)	2.31 (0.09)	7.37 (0.35)	1.05 (0.05)	6.39 (0.25)	1.3 (0.06)	3.6 (0.18)	0.51 (0.03)	3.06 (0.19)	0.46 (0.04)	4.54 (0.21)	1.52 (0.05)	1.78 (0.12)	1.58 (0.07)	0.36 (0.03)
S-9816-09	28.64 (0.82)	267.13 (4.44)	43.58 (1.06)	73.33 (2.37)	60.35 (1.7)	89 (3.1)	22.04 (0.71)		14.46 (0.45)	361.88 (4.83)	35.04 (0.71)	191.38 (3)	24.16 (0.41)	165.57 (3.39)	17.48 (0.4)	42.15 (0.72)	5.77 (0.16)	27.68 (0.75)	5.83 (0.3)	2.18 (0.1)	6.78 (0.31)	1.04 (0.05)	6.46 (0.23)	1.25 (0.06)	3.72 (0.14)	0.58 (0.04)	3.26 (0.22)	0.51 (0.03)	4.86 (0.25)	1.54 (0.04)	1.52 (0.11)	1.56 (0.07)	0.53 (0.04)
**S-9816**	**25.56**	**262.66**	**42.57**	**68.53**	**53.28**	**134.41**	**21.95**		**14.62**	**336.29**	**31.20**	**174.10**	**17.27**	**142.23**	**16.35**	**37.68**	**5.15**	**23.81**	**5.89**	**1.97**	**6.18**	**0.97**	**6.09**	**1.25**	**3.21**	**0.46**	**3.09**	**0.43**	**4.31**	**1.03**	**1.62**	**1.49**	**0.442504518**
S-11218-01	28.68 (0.69)	265.9 (4.27)	42.68 (0.8)	66.46 (2.1)	59.65 (1.61)	84.94 (3.35)	22.54 (0.59)	1.99 (0.42)	14.03 (0.35)	380.51 (5.41)	34.65 (0.53)	197.53 (3)	23.68 (0.36)	167.16 (3.41)	18.87 (0.39)	44.59 (0.77)	5.96 (0.14)	26.25 (0.6)	7.02 (0.37)	2.35 (0.09)	7.52 (0.35)	0.96 (0.05)	7.13 (0.3)	1.31 (0.06)	3.72 (0.15)	0.5 (0.03)	3.12 (0.2)	0.44 (0.03)	4.75 (0.27)	1.54 (0.05)	1.5 (0.12)	1.37 (0.08)	0.46 (0.04)
S-11218-07	29.54 (0.87)	260.02 (4.16)	43.54 (1.11)	66.35 (2.04)	62.15 (1.98)	83.65 (3.23)	21.72 (0.71)	1.55 (0.62)	14.01 (0.44)	370.2 (5.79)	34.04 (0.62)	191.03 (3.06)	22.52 (0.39)	166.72 (3.21)	18.06 (0.37)	43.24 (0.68)	5.89 (0.16)	27.28 (0.82)	6.34 (0.38)	2.07 (0.09)	6.48 (0.34)	0.95 (0.04)	6.95 (0.3)	1.29 (0.06)	3.81 (0.16)	0.55 (0.03)	3.18 (0.19)	0.49 (0.04)	4.42 (0.21)	1.56 (0.05)	1.74 (0.09)	1.6 (0.08)	0.56 (0.04)
S-11218-10	26.83 (0.83)	257.48 (4.44)	43.07 (1.07)	64.41 (2.34)	55.04 (1.69)	90.68 (3.15)	23.74 (0.88)	2.39 (0.64)	14.04 (0.41)	366.41 (5.13)	33.32 (0.67)	192.29 (3.22)	24.06 (0.42)	170.2 (3.37)	17.54 (0.38)	42.98 (0.81)	5.64 (0.14)	26.52 (0.77)	5.92 (0.34)	2.25 (0.11)	7.14 (0.44)	1.05 (0.05)	6.51 (0.26)	1.36 (0.08)	3.71 (0.17)	0.52 (0.03)	3.16 (0.18)	0.48 (0.03)	4.72 (0.2)	1.42 (0.05)	1.73 (0.1)	1.6 (0.07)	0.48 (0.03)
**S-11218**	**28.35 (1.26)**	**261.13 (4.2)**	**43.1 (0.43)**	**65.74 (1.1)**	**58.95 (3.23)**	**86.42 (4.69)**	**22.67 (0.99)**		**14.02 (0.02)**	**372.37 (6.78)**	**34.01 (0.6)**	**193.62 (3.13)**	**23.42 (0.59)**	**168.03 (1.64)**	**18.15 (0.62)**	**43.61 (0.77)**	**5.83 (0.15)**	**26.69 (0.49)**	**6.43 (0.52)**	**2.22 (0.13)**	**7.05 (0.47)**	**0.99 (0.05)**	**6.86 (0.29)**	**1.32 (0.03)**	**3.75 (0.05)**	**0.52 (0.02)**	**3.15 (0.03)**	**0.47 (0.02)**	**4.63 (0.17)**	**1.51 (0.05)**	**1.65 (0.14)**	**1.52 (0.13)**	**0.5 (0.05)**
S-9820-04	26.95 (0.77)	264.89 (4.46)	42.27 (1.09)	58.95 (2.27)	59.35 (1.73)	83.56 (2.75)	21.56 (0.73)	1.32 (0.5)	13.82 (0.37)	371.98 (4.92)	33.75 (0.65)	190.18 (3.29)	23.3 (0.38)	165.39 (3.09)	17.34 (0.4)	42.1 (0.79)	5.53 (0.15)	26.74 (0.82)	6.01 (0.33)	2.08 (0.09)	7.08 (0.33)	0.99 (0.05)	6.59 (0.26)	1.29 (0.06)	3.64 (0.14)	0.53 (0.03)	3.53 (0.2)	0.45 (0.03)	4.67 (0.22)	1.39 (0.04)	1.71 (0.1)	1.46 (0.07)	0.42 (0.03)
S-9820-08	27.55 (0.94)	265.18 (7.07)	36.64 (1.13)	44.56 (1.91)	60.22 (2.12)	85.02 (3.55)	23.3 (0.65)	1.28 (0.52)	15.1 (0.53)	366.24 (6.58)	35.29 (0.9)	200.91 (5.58)	24.34 (0.55)	181.02 (4.15)	18.86 (0.54)	46.54 (1.16)	5.92 (0.2)	27.38 (0.81)	6.53 (0.37)	2.08 (0.1)	6.66 (0.31)	1.01 (0.05)	6.59 (0.34)	1.3 (0.06)	3.69 (0.16)	0.49 (0.03)	3.16 (0.2)	0.45 (0.03)	4.72 (0.23)	1.57 (0.05)	1.56 (0.1)	1.68 (0.09)	0.54 (0.04)
S-9820-09	32.51 (0.68)	300.7 (4.75)	43.66 (0.84)	65.47 (1.75)	73.11 (1.76)	84.72 (3.73)	22.19 (0.6)	1.46 (0.51)	13.42 (0.35)	333.17 (4.52)	33.84 (0.57)	182.96 (2.6)	21.35 (0.34)	150.55 (2.96)	17.19 (0.33)	40.46 (0.61)	5.37 (0.13)	23.74 (0.64)	6.69 (0.33)	2.09 (0.09)	7.17 (0.27)	1.06 (0.04)	6.82 (0.23)	1.27 (0.06)	3.91 (0.15)	0.56 (0.03)	2.98 (0.16)	0.44 (0.03)	4.74 (0.21)	1.34 (0.04)	1.51 (0.11)	1.44 (0.06)	0.49 (0.04)
**S-9820**	**29 (3.05)**	**276.92 (20.59)**	**40.86 (3.72)**	**56.33 (10.7)**	**64.23 (7.71)**	**84.44 (0.77)**	**22.35 (0.88)**		**14.11 (0.88)**	**357.13 (20.94)**	**34.29 (0.87)**	**191.35 (9.03)**	**23 (1.52)**	**165.65 (15.23)**	**17.79 (0.92)**	**43.03 (3.15)**	**5.61 (0.29)**	**25.95 (1.95)**	**6.41 (0.36)**	**2.08 (0.01)**	**6.97 (0.27)**	**1.02 (0.03)**	**6.67 (0.13)**	**1.28 (0.01)**	**3.75 (0.15)**	**0.53 (0.03)**	**3.22 (0.28)**	**0.45 (0)**	**4.71 (0.04)**	**1.43 (0.12)**	**1.59 (0.11)**	**1.53 (0.13)**	**0.48 (0.06)**
	S2G-Sc	S2G-V	S2G-Co		S2G-Cu	S2G-Zn	S2G-Ga		S2G-Rb	S2G-Sr	S2G-Y	S2G-Zr	S2G-Nb	S2G-Ba	S2G-La	S2G-Ce	S2G-Pr	S2G-Nd	S2G-Sm	S2G-Eu	S2G-Gd	S2G-Tb	S2G-Dy	S2G-Ho	S2G-Er	S2G-Tm	S2G-Yb	S2G-Lu	S2G-Hf	S2G-Ta	S2G-Pb	S2G-Th	S2G-U
S-11239-03	29.25 (0.83)	268.02 (4.66)	41.63 (0.97)	66.45 (2.35)	63.77 (2.55)	90.73 (3.34)	23.02 (0.67)		14.57 (0.47)	367.98 (5.04)	34.73 (0.67)	199.5 (3.69)	23.79 (0.42)	163.86 (3.46)	17.77 (0.41)	43.24 (0.77)	6.01 (0.16)	26.5 (0.67)	6.47 (0.35)	2.22 (0.12)	6.84 (0.34)	1.03 (0.06)	7.22 (0.29)	1.28 (0.07)	3.73 (0.16)	0.52 (0.03)	3.47 (0.21)	0.42 (0.03)	4.68 (0.19)	1.49 (0.05)	1.67 (0.1)	1.59 (0.07)	0.57 (0.04)
S-11239-08	27.75 (0.9)	268.23 (8.02)	40.72 (1.75)	69.08 (3.28)	52.23 (3.1)	88.81 (4.56)	23.02 (1.01)	1.23 (1.32)	13.26 (0.51)	361.78 (8.69)	34.16 (1.03)	200.91 (5.57)	24.3 (0.75)	167.08 (5.66)	17.86 (0.71)	44.3 (1.49)	5.62 (0.18)	27.6 (1.38)	7.22 (0.96)	2.21 (0.16)	6.1 (0.51)	0.91 (0.07)	6.27 (0.4)	1.24 (0.08)	3.46 (0.24)	0.49 (0.05)	2.86 (0.31)	0.44 (0.07)	5.36 (0.54)	1.49 (0.06)	1.82 (0.13)	1.59 (0.15)	0.51 (0.09)
S-11239-09	27.84 (0.65)	266.8 (4.05)	41.4 (0.95)	65.73 (1.87)	59.57 (1.47)	86.24 (3.64)	23.26 (0.6)	1.4 (0.45)	14 (0.39)	372.13 (5.23)	34.76 (0.57)	197.33 (3.16)	23.83 (0.37)	167.28 (3.22)	18.85 (0.39)	44.99 (0.65)	6.15 (0.17)	27.25 (0.66)	6.39 (0.37)	2.28 (0.09)	7.42 (0.35)	1.01 (0.05)	6.57 (0.26)	1.3 (0.06)	3.74 (0.15)	0.55 (0.04)	3.16 (0.19)	0.45 (0.03)	4.52 (0.23)	1.46 (0.05)	1.75 (0.12)	1.6 (0.07)	0.44 (0.04)
**S-11239**	**28.28 (0.76)**	**267.68 (0.75)**	**41.25 (0.47)**	**67.09 (1.7)**	**58.52 (5.23)**	**88.59 (2.83)**	**23.1 (0.13)**		**13.95 (0.68)**	**367.3 (4.83)**	**34.55 (0.3)**	**199.25 (1.64)**	**23.97 (0.21)**	**166.07 (1.66)**	**18.16 (0.55)**	**44.18 (0.79)**	**5.92 (0.24)**	**27.12 (0.51)**	**6.69 (0.43)**	**2.24 (0.03)**	**6.79 (0.59)**	**0.98 (0.06)**	**6.68 (0.45)**	**1.27 (0.03)**	**3.64 (0.14)**	**0.52 (0.03)**	**3.16 (0.29)**	**0.44 (0.01)**	**4.85 (0.42)**	**1.48 (0.01)**	**1.75 (0.07)**	**1.59 (0.01)**	**0.51 (0.06)**

S-9822-01	46.97 (0.2)	2.45 (0.06)	16.12 (0.01)	12.35 (0.38)	0.19 (0.01)	6.31 (0.01)	10.27 (0.04)	3.49 (0.06)	0.59 (0.02)	0.02 (0.02)	0.17 (0.06)	0.12 (0.04)	99.04	3
S-9822-02	47.18 (0.13)	2.51 (0.02)	15.94 (0.02)	12.38 (0.22)	0.2 (0.05)	6.28 (0.04)	10.2 (0.1)	3.63 (0.16)	0.58 (0.02)	0.04 (0.01)	0.16 (0.05)	0.11 (0.03)	99.20	3
S-9822-03	46.84 (0.1)	2.51 (0.04)	15.87 (0.05)	12.73 (0.23)	0.22 (0.04)	6.35 (0.02)	10.22 (0.1)	3.42 (0.11)	0.58 (0.01)	0.01 (0.01)	0.22 (0.03)	0.11 (0.01)	99.09	2
S-9822-04	47.09 (0.2)	2.54 (0.04)	15.94 (0.06)	12.52 (0.17)	0.21 (0.01)	6.24 (0.1)	10.26 (0.03)	3.49 (0.05)	0.6 (0.02)	0.02 (0.02)	0.19 (0.05)	0.11 (0)	99.20	3
S-9822-05	46.89 (0.16)	2.52 (0.06)	16.01 (0.1)	12.13 (0.16)	0.18 (0.02)	6.27 (0.07)	10.17 (0.02)	3.54 (0.09)	0.61 (0.01)	0.02 (0.02)	0.21 (0.03)	0.11 (0.01)	98.64	3
S-9822-07	46.92 (0.06)	2.67 (0.02)	15.65 (0.02)	12.65 (0.27)	0.18 (0.04)	6.06 (0.09)	9.91 (0.08)	3.56 (0.11)	0.64 (0.04)	0.03 (0.01)	0.23 (0.05)	0.1 (0.01)	98.59	3
S-9822-08	46.98 (0.1)	2.61 (0.06)	15.9 (0.16)	12.47 (0.42)	0.21 (0.02)	6.21 (0.02)	10.03 (0.07)	3.67 (0.08)	0.59 (0.04)	0.02 (0)	0.21 (0.04)	0.11 (0.01)	99.00	3
S-9822-09	46.9 (0.16)	2.52 (0.14)	15.95 (0.07)	12.15 (0.33)	0.17 (0.03)	6.34 (0.1)	10.26 (0.06)	3.51 (0.06)	0.57 (0.02)	0.03 (0.04)	0.2 (0.03)	0.11 (0.02)	98.72	3
S-9822-10	47.18 (0.14)	2.51 (0.02)	15.92 (0.08)	12.44 (0.27)	0.19 (0.01)	6.22 (0.12)	10.24 (0.06)	3.6 (0.02)	0.6 (0.01)	0.03 (0.02)	0.19 (0.03)	0.09 (0.03)	99.21	3
**S-9822**	**46.99 (0.13)**	**2.54 (0.06)**	**15.92 (0.12)**	**12.42 (0.2)**	**0.19 (0.02)**	**6.25 (0.09)**	**10.17 (0.12)**	**3.54 (0.08)**	**0.59 (0.02)**	**0.02 (0.01)**	**0.2 (0.02)**	**0.11 (0.01)**	**98.97**	**26**
S-9821-02	47.38 (0.17)	2.79 (0.02)	15.79 (0.12)	12.72 (0.54)	0.21 (0.02)	5.86 (0.11)	9.28 (0.02)	3.9 (0.07)	0.7 (0.01)	0.03 (0.01)	0.19 (0.02)	0.1 (0.01)	98.96	3
S-9821-03	47.22 (0.08)	2.81 (0.03)	15.71 (0.09)	13.09 (0.34)	0.19 (0.01)	5.76 (0.05)	9.29 (0.08)	3.83 (0.16)	0.71 (0.04)	0.02 (0.02)	0.21 (0.02)	0.1 (0.01)	98.94	3
S-9821-04	46.36 (0.36)	2.92 (0.04)	15.9 (0.05)	12.79 (0.02)	0.19 (0.01)	5.78 (0.08)	9.3 (0.06)	3.96 (0.16)	0.69 (0)	0.03 (0.01)	0.19 (0.02)	0.1 (0)	98.20	2
S-9821-05	47.24 (0.02)	2.78 (0.03)	15.81 (0.05)	12.88 (0.35)	0.21 (0.03)	5.66 (0.03)	9.23 (0.08)	3.85 (0.13)	0.69 (0.01)	0.03 (0.03)	0.24 (0.01)	0.1 (0.02)	98.71	3
S-9821-06	47.39 (0.14)	2.74 (0.04)	15.84 (0.09)	13.2 (0.14)	0.18 (0.01)	5.69 (0.13)	9.32 (0.1)	3.8 (0.06)	0.73 (0.02)	0.01 (0.01)	0.2 (0.02)	0.1 (0.02)	99.19	3
S-9821-07	46.75 (0.1)	2.64 (0.05)	16.45 (0.14)	12.7 (0.06)	0.2 (0.02)	5.98 (0.15)	9.69 (0.07)	3.7 (0.09)	0.65 (0.01)	0.03 (0.01)	0.19 (0.03)	0.1 (0.01)	99.08	3
S-9821-08	47.36 (0.02)	2.78 (0.12)	15.73 (0.02)	13.39 (0.2)	0.2 (0.04)	5.66 (0.07)	9.29 (0.08)	3.8 (0.09)	0.71 (0.01)	0.01 (0.02)	0.22 (0.04)	0.1 (0.01)	99.24	3
S-9821-09	47.14 (0.2)	2.62 (0.04)	15.9 (0.08)	12.5 (0.05)	0.19 (0.02)	5.96 (0.06)	9.66 (0.04)	3.74 (0.08)	0.66 (0.01)	0.02 (0.03)	0.22 (0.01)	0.1 (0.02)	98.71	3
S-9821-10	47.12 (0.13)	2.78 (0.02)	15.67 (0.02)	13.21 (0.24)	0.18 (0.02)	6.01 (0.44)	9.2 (0.18)	3.83 (0.07)	0.73 (0.04)	0.03 (0.02)	0.21 (0.02)	0.1 (0.01)	99.07	3
**S-9821**	**47.11 (0.34)**	**2.76 (0.09)**	**15.87 (0.23)**	**12.94 (0.29)**	**0.2 (0.01)**	**5.82 (0.14)**	**9.36 (0.18)**	**3.82 (0.08)**	**0.7 (0.03)**	**0.02 (0.01)**	**0.21 (0.02)**	**0.1 (0)**	**98.90**	**26**
Syrt-9832-01	47.44 (0.08)	2.24 (0.04)	15.54 (0.11)	11.16 (0.31)	0.17 (0.02)	7.05 (0.12)	11.91 (0.17)	2.95 (0.15)	0.46 (0)	0.04 (0.03)	0.19 (0.05)	0.11 (0.01)	99.26	3
Syrt-9832-02	47.44 (0.24)	2.26 (0.04)	15.59 (0.02)	11.23 (0.4)	0.14 (0)	7.04 (0.18)	11.95 (0.17)	2.83 (0.07)	0.43 (0.02)	0.01 (0.01)	0.17 (0.06)	0.16 (0.02)	99.25	3
Syrt-9832-03	47.44 (0.14)	2.25 (0.02)	15.55 (0.12)	11.1 (0.29)	0.15 (0.01)	6.92 (0.12)	11.93 (0.13)	2.84 (0.04)	0.44 (0.01)	0.01 (0.02)	0.18 (0.03)	0.11 (0.01)	98.93	3
Syrt-9832-04	47.29 (0.16)	2.16 (0.01)	15.54 (0.18)	11.2 (0.12)	0.17 (0.03)	6.97 (0.1)	11.95 (0.19)	2.9 (0.03)	0.44 (0.02)	0.02 (0.02)	0.19 (0.06)	0.15 (0.01)	98.98	3
Syrt-9832-05	47.47 (0.14)	2.24 (0.02)	15.54 (0.12)	11.29 (0.2)	0.18 (0.03)	7.07 (0.13)	11.89 (0.18)	2.91 (0.16)	0.43 (0.04)	0.01 (0.01)	0.16 (0.08)	0.12 (0.02)	99.31	3
Syrt-9832-06	47.5 (0.34)	2.22 (0.04)	15.51 (0.14)	11.17 (0.13)	0.16 (0.02)	7.1 (0.05)	11.89 (0.2)	2.87 (0.12)	0.45 (0.01)	0.05 (0.01)	0.18 (0.03)	0.12 (0.01)	99.21	3
Syrt-9832-07	47.75 (0.12)	2.28 (0.02)	15.52 (0.12)	11.28 (0.72)	0.15 (0.02)	7.14 (0.11)	11.93 (0.26)	3.09 (0.09)	0.43 (0.01)	0.03 (0.04)	0.18 (0.07)	0.12 (0.01)	99.89	3
Syrt-9832-08	47.37 (0.31)	2.25 (0.03)	15.54 (0.22)	10.97 (0.37)	0.18 (0.01)	6.96 (0.19)	11.9 (0.18)	2.86 (0.09)	0.44 (0.02)	0.02 (0)	0.18 (0.08)	0.12 (0.02)	98.80	3
Syrt-9832-09	47.43 (0.32)	2.26 (0.02)	15.54 (0.14)	11.28 (0.07)	0.14 (0.02)	7.04 (0.15)	11.97 (0.17)	2.81 (0.1)	0.42 (0.02)	0.01 (0.01)	0.2 (0.06)	0.14 (0.02)	99.23	3
Syrt-9832-10	47.67 (0.48)	2.23 (0.05)	15.53 (0.16)	11.24 (0.12)	0.16 (0.01)	7.01 (0.21)	11.94 (0.13)	2.92 (0.04)	0.44 (0.03)	0.03 (0)	0.18 (0.04)	0.13 (0.01)	99.48	3
**Syrt-9832**	**47.48 (0.14)**	**2.24 (0.03)**	**15.54 (0.02)**	**11.19 (0.1)**	**0.16 (0.01)**	**7.03 (0.07)**	**11.93 (0.03)**	**2.9 (0.08)**	**0.44 (0.01)**	**0.02 (0.01)**	**0.18 (0.01)**	**0.13 (0.02)**	**99.23**	**30**
Syrt-9833-01	46.81 (0.18)	2.24 (0.05)	15.22 (0.3)	11.1 (0.07)	0.15 (0.04)	7.11 (0.16)	11.88 (0.04)	3.09 (0.06)	0.4 (0.02)	0.02 (0.02)	0.13 (0.03)	0.09 (0.01)	98.25	3
Syrt-9833-02	47.11 (0)	2.23 (0)	15.4 (0)	11.29 (0)	0.15 (0)	6.73 (0)	12.01 (0)	3 (0)	0.46 (0)	0.03 (0)	0.12 (0)	0.1 (0)	98.63	1
Syrt-9833-03	47.52 (0.71)	2.22 (0)	15.42 (0.19)	11.02 (0.12)	0.14 (0.04)	6.88 (0.13)	11.93 (0.08)	3.06 (0.09)	0.4 (0.01)	0.02 (0.01)	0.14 (0.06)	0.1 (0)	98.86	3
Syrt-9833-04	47.39 (0.69)	2.18 (0.07)	15.27 (0.04)	11.14 (0.15)	0.2 (0.07)	7.3 (0.28)	11.91 (0.12)	3.07 (0.08)	0.39 (0.02)	0.02 (0.03)	0.1 (0.02)	0.1 (0.01)	99.08	3
Syrt-9833-05	47.2 (0.61)	2.2 (0.01)	15.11 (0.23)	11.18 (0.08)	0.19 (0.01)	7.14 (0.1)	12.18 (0.03)	3.04 (0.03)	0.39 (0.03)	0.04 (0.01)	0.09 (0.01)	0.09 (0.04)	98.83	3
Syrt-9833-06	47.41 (0.93)	2.21 (0.03)	15.41 (0.11)	11.1 (0.12)	0.17 (0.05)	7.14 (0.22)	11.91 (0.16)	3 (0.08)	0.4 (0.03)	0.02 (0.02)	0.13 (0.06)	0.12 (0.01)	99.03	3
Syrt-9833-07	47.33 (0.75)	2.23 (0.02)	15.42 (0.1)	11.08 (0.16)	0.2 (0.05)	7.19 (0.04)	11.95 (0.08)	3.19 (0.12)	0.41 (0.05)	0.02 (0.01)	0.12 (0.04)	0.12 (0)	99.25	3
Syrt-9833-08	47.31 (0.9)	2.18 (0.06)	15.28 (0.14)	11.03 (0.13)	0.16 (0.02)	7.21 (0.14)	11.92 (0.08)	3.12 (0.13)	0.4 (0.01)	0.03 (0.03)	0.12 (0.02)	0.09 (0.02)	98.85	3
Syrt-9833-09	47.66 (0.5)	2.2 (0.02)	15.45 (0.09)	10.94 (0.42)	0.17 (0.01)	7.14 (0.06)	11.91 (0.12)	3.06 (0.08)	0.41 (0.02)	0.02 (0.02)	0.11 (0.03)	0.11 (0)	99.17	3
**Syrt-9833**	**47.3 (0.25)**	**2.21 (0.02)**	**15.33 (0.12)**	**11.1 (0.1)**	**0.17 (0.02)**	**7.09 (0.18)**	**11.96 (0.09)**	**3.07 (0.06)**	**0.41 (0.02)**	**0.02 (0.01)**	**0.12 (0.02)**	**0.1 (0.01)**	**98.88**	**25**
Syrt-11221-01	47.12 (0.19)	2.26 (0.02)	15.78 (0.13)	11.34 (0.09)	0.15 (0)	6.5 (0.07)	11.92 (0.27)	2.95 (0.03)	0.47 (0.04)	0.06 (0.02)	0.18 (0.05)	0.22 (0.02)	98.94	3
Syrt-11221-02	47.25 (0.06)	2.25 (0.04)	15.53 (0.08)	11.15 (0.31)	0.17 (0)	6.95 (0.13)	11.77 (0.19)	2.96 (0.04)	0.48 (0.02)	0.04 (0.01)	0.17 (0.03)	0.14 (0.01)	98.86	3
Syrt-11221-03	47.29 (0.11)	2.26 (0.07)	15.65 (0.15)	11.26 (0.11)	0.16 (0.03)	6.92 (0.06)	11.82 (0.18)	2.89 (0.16)	0.47 (0.01)	0.04 (0.01)	0.18 (0.03)	0.12 (0.05)	99.08	3
Syrt-11221-04	47.35 (0.03)	2.25 (0.01)	15.51 (0.06)	11 (0.21)	0.17 (0.03)	6.84 (0.25)	11.88 (0.13)	2.97 (0.17)	0.43 (0.02)	0.03 (0.02)	0.17 (0.06)	0.13 (0.01)	98.73	3
Syrt-11221-05	47.38 (0.14)	2.26 (0.04)	15.57 (0.19)	11.27 (0.13)	0.19 (0.01)	6.95 (0.1)	11.78 (0.14)	2.94 (0.09)	0.46 (0)	0.02 (0.01)	0.18 (0.05)	0.11 (0.02)	99.11	3
Syrt-11221-06	47.86 (0.08)	2.23 (0.03)	15.76 (0.12)	11.53 (0.09)	0.17 (0.04)	7.07 (0.05)	11.73 (0.02)	2.78 (0.06)	0.37 (0.02)	0.03 (0.01)	0.13 (0.01)	0.11 (0)	99.77	3
Syrt-11221-07	47.01 (0.42)	2.23 (0.02)	15.49 (0.16)	10.75 (0.49)	0.19 (0.04)	6.93 (0.16)	11.8 (0.14)	2.97 (0.02)	0.45 (0.01)	0.02 (0.02)	0.16 (0.07)	0.13 (0.02)	98.13	3
Syrt-11221-08	46.89 (0.58)	2.23 (0.03)	15.47 (0.12)	11.11 (0.44)	0.18 (0.03)	6.96 (0.02)	11.77 (0.11)	2.93 (0.08)	0.43 (0.01)	0.06 (0.02)	0.15 (0.04)	0.13 (0.01)	98.29	3
Syrt-11221-09	47.1 (0.18)	2.25 (0.02)	15.48 (0.12)	11.25 (0.09)	0.14 (0.02)	6.93 (0.05)	11.82 (0.14)	2.98 (0.17)	0.46 (0.03)	0.02 (0.01)	0.17 (0.04)	0.12 (0.01)	98.71	3
Syrt-11221-10	47.17 (0.32)	2.27 (0.01)	15.45 (0.08)	11.02 (0.27)	0.17 (0.03)	6.74 (0.14)	11.97 (0.08)	2.89 (0.11)	0.45 (0.02)	0.04 (0.01)	0.2 (0.02)	0.11 (0.03)	98.48	3
**Syrt-11221**	**47.24 (0.27)**	**2.25 (0.01)**	**15.57 (0.12)**	**11.17 (0.21)**	**0.17 (0.01)**	**6.88 (0.16)**	**11.83 (0.08)**	**2.93 (0.06)**	**0.45 (0.03)**	**0.03 (0.01)**	**0.17 (0.02)**	**0.13 (0.03)**	**98.81**	**30**
Syrt-9834-01	47.52 (0.03)	2.28 (0.02)	15.6 (0.1)	11.4 (0.23)	0.18 (0.03)	6.99 (0.05)	11.79 (0.17)	2.99 (0.05)	0.45 (0.01)	0.02 (0.02)	0.18 (0.06)	0.12 (0.01)	99.52	3
Syrt-9834-02	47.51 (0.08)	2.3 (0.04)	15.59 (0.13)	11.32 (0.1)	0.16 (0.02)	6.9 (0.05)	11.74 (0.17)	3.01 (0.05)	0.47 (0.03)	0.01 (0.01)	0.2 (0.09)	0.13 (0.02)	99.33	3
Syrt-9834-03	47.53 (0.49)	2.26 (0.03)	15.67 (0.08)	11.15 (0.15)	0.19 (0.03)	7.05 (0.13)	11.86 (0.2)	2.96 (0.12)	0.43 (0.02)	0.06 (0.03)	0.17 (0.04)	0.1 (0.02)	99.42	3
Syrt-9834-04	47.31 (0.13)	2.21 (0.11)	15.55 (0.01)	10.93 (0.11)	0.17 (0.03)	7 (0.01)	11.66 (0.03)	2.94 (0.07)	0.47 (0)	0.01 (0.02)	0.23 (0.05)	0.14 (0.02)	98.63	2
Syrt-9834-06	47.3 (0.23)	2.28 (0.03)	15.62 (0.02)	11.52 (0.12)	0.15 (0.01)	6.89 (0.09)	11.76 (0.17)	3.01 (0.07)	0.47 (0)	0.03 (0.02)	0.2 (0.06)	0.13 (0.01)	99.36	3
Syrt-9834-07	47.32 (0.22)	2.3 (0.06)	15.45 (0.19)	11.17 (0.15)	0.19 (0)	6.92 (0.13)	11.73 (0.15)	3.04 (0.08)	0.47 (0.01)	0.02 (0.02)	0.16 (0.05)	0.11 (0.01)	98.88	3
Syrt-9834-08	47.62 (0.25)	2.31 (0.06)	15.59 (0.1)	10.82 (0.05)	0.16 (0.01)	7.02 (0.07)	11.69 (0.2)	3.01 (0.03)	0.46 (0.03)	0.03 (0.04)	0.21 (0.04)	0.12 (0.01)	99.04	3
Syrt-9834-09	47.21 (0.46)	2.31 (0.06)	15.44 (0.25)	11.26 (0.09)	0.17 (0.01)	6.94 (0.07)	11.9 (0.24)	2.98 (0.06)	0.42 (0.02)	0.04 (0.01)	0.18 (0.06)	0.14 (0.04)	99.00	3
Syrt-9834-10	47.18 (0.08)	2.3 (0.06)	15.58 (0.13)	11.21 (0.34)	0.17 (0.02)	6.92 (0.03)	11.82 (0.08)	3.05 (0.03)	0.47 (0.02)	0.04 (0.02)	0.19 (0.03)	0.1 (0.03)	99.04	3
**Syrt-9834**	**47.39 (0.16)**	**2.28 (0.03)**	**15.57 (0.07)**	**11.2 (0.22)**	**0.17 (0.01)**	**6.96 (0.06)**	**11.77 (0.08)**	**3 (0.03)**	**0.46 (0.02)**	**0.03 (0.01)**	**0.19 (0.02)**	**0.12 (0.01)**	**99.13**	**26**
Syrt-9831-01	47.38 (0.58)	2.19 (0.05)	15.48 (0.23)	10.95 (0.25)	0.16 (0.02)	7.02 (0.02)	11.9 (0.24)	2.92 (0.09)	0.44 (0.01)	0.03 (0.02)	0.2 (0.04)	0.12 (0.03)	98.78	3
Syrt-9831-02	47.44 (0.16)	2.25 (0.02)	15.54 (0.02)	11.11 (0.31)	0.16 (0.01)	7.03 (0.09)	11.98 (0.16)	2.91 (0.21)	0.44 (0.02)	0.02 (0.02)	0.18 (0.04)	0.12 (0.02)	99.18	3
Syrt-9831-03	47.38 (0.27)	2.27 (0.03)	15.53 (0.11)	10.99 (0.18)	0.16 (0)	6.72 (0.33)	11.89 (0.18)	2.9 (0.14)	0.44 (0.02)	0.04 (0.03)	0.18 (0.04)	0.12 (0)	98.63	3
Syrt-9831-04	47.21 (0.08)	2.21 (0.07)	15.46 (0.13)	10.81 (0.61)	0.16 (0.04)	7.02 (0.08)	11.92 (0.17)	2.87 (0.11)	0.42 (0.02)	0.03 (0.01)	0.17 (0.08)	0.13 (0.02)	98.40	3
Syrt-9831-05	47.16 (0.33)	2.21 (0.01)	15.39 (0.18)	10.75 (0.41)	0.15 (0.02)	7.04 (0.32)	11.69 (0.45)	2.9 (0.03)	0.45 (0.02)	0.02 (0)	0.23 (0.02)	0.15 (0.04)	98.14	2
Syrt-9831-06	47.44 (0.09)	2.23 (0.03)	15.51 (0.15)	11.13 (0.19)	0.18 (0.02)	6.92 (0.27)	11.98 (0.17)	2.83 (0.09)	0.42 (0.03)	0.01 (0.01)	0.2 (0.08)	0.13 (0.02)	98.99	3
Syrt-9831-07	47.38 (0.45)	2.2 (0.07)	15.52 (0.19)	11.04 (0.46)	0.14 (0.02)	6.93 (0.09)	11.97 (0.15)	2.86 (0.17)	0.42 (0)	0.02 (0.02)	0.21 (0.06)	0.13 (0.02)	98.81	3
Syrt-9831-08	47.3 (0.29)	2.25 (0.02)	15.52 (0.14)	11.11 (0.52)	0.19 (0.02)	6.84 (0.46)	12.13 (0.45)	2.75 (0.04)	0.41 (0.05)	0.02 (0.02)	0.18 (0.07)	0.18 (0.07)	98.88	3
Syrt-9831-09	47.52 (0.58)	2.28 (0.07)	15.57 (0.25)	11.08 (0.24)	0.17 (0.02)	6.94 (0.06)	11.95 (0.11)	2.93 (0.05)	0.45 (0.01)	0.03 (0.02)	0.17 (0.08)	0.11 (0.01)	99.18	3
Syrt-9831-10	47.43 (0.44)	2.24 (0.04)	15.4 (0.09)	11.23 (0)	0.15 (0.03)	7.01 (0.18)	11.94 (0.18)	2.87 (0.06)	0.44 (0.02)	0.02 (0.01)	0.16 (0.03)	0.1 (0.01)	98.98	3
**Syrt-9831**	**47.36 (0.11)**	**2.23 (0.03)**	**15.49 (0.06)**	**11.02 (0.15)**	**0.16 (0.01)**	**6.95 (0.1)**	**11.93 (0.11)**	**2.87 (0.05)**	**0.43 (0.01)**	**0.02 (0.01)**	**0.19 (0.02)**	**0.13 (0.02)**	**98.80**	**29**
Syrt-9835-01	47.4 (0.17)	2.29 (0.04)	15.43 (0.07)	11.24 (0.35)	0.17 (0.02)	6.81 (0.42)	11.78 (0.35)	3.04 (0.1)	0.48 (0.04)	0.05 (0.02)	0.17 (0.03)	0.13 (0.02)	98.99	3
Syrt-9835-02	47.1 (0.4)	2.33 (0.01)	15.33 (0.25)	11.17 (0.04)	0.15 (0.02)	6.95 (0.06)	11.66 (0.08)	3.01 (0.07)	0.47 (0.01)	0.03 (0)	0.17 (0.04)	0.14 (0.03)	98.53	3
Syrt-9835-03	47.27 (0.36)	2.31 (0.04)	15.43 (0.11)	11.43 (0.14)	0.17 (0.04)	6.82 (0.12)	11.75 (0.1)	3.08 (0.1)	0.45 (0.02)	0.02 (0.03)	0.19 (0.03)	0.12 (0)	99.05	3
Syrt-9835-04	46.85 (0.52)	2.3 (0.04)	15.41 (0.14)	11.22 (0.08)	0.17 (0.05)	6.91 (0.08)	11.71 (0.17)	3.01 (0.11)	0.45 (0.03)	0.03 (0.02)	0.19 (0.05)	0.13 (0.01)	98.37	3
Syrt-9835-05	47.44 (0.26)	2.32 (0.03)	15.39 (0.08)	11.16 (0.06)	0.16 (0.01)	6.88 (0.02)	11.77 (0.19)	3.04 (0.01)	0.47 (0.01)	0.02 (0.02)	0.17 (0.02)	0.12 (0.02)	98.95	3
Syrt-9835-06	47.51 (0.07)	2.31 (0.05)	15.53 (0.14)	11.09 (0.14)	0.16 (0.01)	6.95 (0.03)	11.77 (0.14)	3.07 (0.05)	0.46 (0.02)	0.02 (0.02)	0.18 (0.02)	0.12 (0)	99.14	3
Syrt-9835-07	47.37 (0.01)	2.29 (0.07)	15.48 (0.06)	10.89 (0.37)	0.15 (0.02)	6.91 (0.05)	11.71 (0.07)	2.86 (0.08)	0.44 (0.02)	0.04 (0.05)	0.21 (0.02)	0.14 (0.01)	98.49	2
Syrt-9835-08	47.39 (0.26)	2.2 (0.09)	15.6 (0.16)	11.16 (0.07)	0.15 (0.03)	6.99 (0.08)	11.73 (0.1)	3.04 (0.12)	0.45 (0.01)	0.01 (0.01)	0.19 (0.05)	0.15 (0.02)	99.08	3
Syrt-9835-09	47.38 (0.17)	2.34 (0.02)	15.48 (0.09)	11.16 (0.22)	0.19 (0.02)	6.92 (0.08)	11.75 (0.14)	3 (0.1)	0.44 (0.02)	0.02 (0)	0.17 (0.01)	0.15 (0.03)	99.00	3
Syrt-9835-10	47.31 (0.13)	2.35 (0.02)	15.42 (0.15)	11.08 (0.47)	0.16 (0.01)	6.88 (0.05)	11.69 (0.19)	2.88 (0.11)	0.45 (0.02)	0.02 (0.02)	0.2 (0.06)	0.13 (0.03)	98.57	3
**Syrt-9835**	**47.3 (0.19)**	**2.3 (0.04)**	**15.45 (0.08)**	**11.16 (0.14)**	**0.16 (0.01)**	**6.9 (0.06)**	**11.73 (0.04)**	**3.01 (0.07)**	**0.45 (0.01)**	**0.03 (0.01)**	**0.18 (0.01)**	**0.13 (0.01)**	**98.82**	**29**
Jol-11222-01	47.76 (0.04)	2.39 (0)	15.29 (0.07)	11.29 (0.16)	0.12 (0.01)	6.93 (0.07)	11.54 (0.18)	2.94 (0.08)	0.41 (0.03)	0.03 (0.02)	0.18 (0.05)	0.11 (0.01)	98.99	3
Jol-11222-02	47.76 (0.4)	2.36 (0.06)	15.31 (0.26)	11.39 (0.23)	0.14 (0)	6.89 (0.18)	11.51 (0)	3.13 (0.01)	0.41 (0.04)	0.05 (0.02)	0.2 (0.03)	0.11 (0.02)	99.26	2
Jol-11222-04	48.03 (0.36)	2.36 (0.04)	15.31 (0.22)	11.21 (0.11)	0.17 (0.02)	6.93 (0.11)	11.55 (0.18)	3.03 (0.09)	0.4 (0.01)	0.03 (0.02)	0.18 (0.04)	0.1 (0.01)	99.29	3
Jol-11222-05	47.72 (0.42)	2.34 (0.02)	15.28 (0.26)	11.55 (0.22)	0.18 (0.01)	7 (0)	11.72 (0.08)	2.8 (0.21)	0.41 (0.03)	0.04 (0.03)	0.16 (0.01)	0.13 (0)	99.31	2
Jol-11222-06	47.79 (0.55)	2.33 (0.05)	15.35 (0.22)	11.38 (0.2)	0.19 (0.03)	6.84 (0.14)	11.59 (0.25)	2.89 (0.1)	0.42 (0.02)	0.11 (0.06)	0.17 (0.04)	0.13 (0.02)	99.20	3
Jol-11222-07	47.98 (0.09)	2.34 (0.02)	15.47 (0)	11.38 (0.09)	0.14 (0.02)	6.88 (0.02)	11.59 (0.03)	3.14 (0.05)	0.45 (0.01)	0.02 (0.02)	0.25 (0)	0.14 (0.02)	99.80	2
Jol-11222-08	48 (0.15)	2.42 (0.01)	15.35 (0.09)	11.17 (0.17)	0.17 (0.02)	6.96 (0.08)	11.58 (0.05)	3.06 (0.09)	0.43 (0.04)	0.02 (0.02)	0.18 (0.06)	0.1 (0.04)	99.43	3
**Jol-11222**	**47.86 (0.14)**	**2.36 (0.03)**	**15.34 (0.07)**	**11.34 (0.13)**	**0.16 (0.02)**	**6.92 (0.05)**	**11.58 (0.07)**	**3 (0.13)**	**0.42 (0.02)**	**0.04 (0.03)**	**0.19 (0.03)**	**0.12 (0.02)**	**99.33**	**18**
Jol-9836-01	47.85 (0.27)	2.38 (0.05)	15.26 (0.28)	11.27 (0.06)	0.16 (0.02)	7.04 (0.21)	11.62 (0.07)	2.85 (0.07)	0.4 (0.04)	0.02 (0.03)	0.16 (0.02)	0.12 (0.01)	99.14	3
Jol-9836-02	47.86 (0.15)	2.22 (0.15)	15.31 (0.17)	11.26 (0.25)	0.17 (0.04)	6.96 (0.08)	11.51 (0.15)	2.87 (0.1)	0.38 (0.04)	0 (0.01)	0.19 (0.05)	0.12 (0.02)	98.85	3
Jol-9836-03	47.93 (0.12)	2.36 (0.06)	15.22 (0.17)	11.14 (0.32)	0.16 (0.02)	7.08 (0.11)	11.58 (0.14)	2.93 (0.08)	0.42 (0.02)	0.01 (0.01)	0.17 (0.06)	0.12 (0.02)	99.12	3
Jol-9836-04	47.95 (0.11)	2.32 (0.02)	15.23 (0.07)	11.4 (0.09)	0.18 (0.02)	7 (0.11)	11.61 (0.15)	2.89 (0.12)	0.42 (0.02)	0.04 (0.01)	0.19 (0.04)	0.13 (0.02)	99.35	3
Jol-9836-05	48.17 (0.15)	2.38 (0.06)	15.31 (0.15)	11.21 (0.12)	0.17 (0.03)	7.05 (0.07)	11.64 (0.08)	2.92 (0.1)	0.4 (0.02)	0.03 (0.02)	0.14 (0.03)	0.11 (0.01)	99.52	3
Jol-9836-06	48.07 (0.16)	2.39 (0.02)	15.35 (0.07)	11.36 (0.06)	0.16 (0.02)	7 (0.06)	11.59 (0.13)	2.97 (0.09)	0.41 (0.01)	0.03 (0.02)	0.18 (0.07)	0.12 (0.03)	99.63	3
Jol-9836-07	47.73 (0.6)	2.37 (0.02)	15.09 (0.29)	11.29 (0.2)	0.15 (0.02)	6.87 (0.13)	11.56 (0.12)	2.96 (0.03)	0.43 (0.02)	0.03 (0.02)	0.18 (0.05)	0.13 (0.02)	98.79	3
Jol-9836-08	47.83 (0.06)	2.39 (0.02)	15.34 (0.16)	11.22 (0.35)	0.16 (0.01)	6.93 (0.02)	11.52 (0.19)	3.03 (0.06)	0.41 (0.01)	0.02 (0.02)	0.19 (0.04)	0.12 (0.02)	99.17	3
Jol-9836-09	47.63 (0.29)	2.35 (0.02)	15.23 (0.15)	11.44 (0.09)	0.14 (0.02)	6.87 (0.04)	11.57 (0.15)	2.87 (0.02)	0.4 (0.02)	0.03 (0.02)	0.18 (0.07)	0.14 (0.03)	98.85	3
Jol-9836-10	47.81 (0.39)	2.36 (0.04)	15.25 (0.28)	11.17 (0.08)	0.16 (0.01)	6.97 (0.1)	11.56 (0.03)	2.94 (0.14)	0.41 (0.02)	0.02 (0.02)	0.18 (0.05)	0.14 (0.01)	98.97	3
**Jol-9836**	**47.88 (0.16)**	**2.35 (0.05)**	**15.26 (0.08)**	**11.28 (0.1)**	**0.16 (0.01)**	**6.98 (0.07)**	**11.58 (0.04)**	**2.92 (0.05)**	**0.41 (0.01)**	**0.02 (0.01)**	**0.18 (0.02)**	**0.12 (0.01)**	**99.14**	**30**
Jol-9837-01	47.75 (0.68)	2.33 (0.04)	15.23 (0.36)	11.04 (0.18)	0.13 (0.03)	7.03 (0.02)	11.6 (0.11)	2.97 (0)	0.41 (0.02)	0.02 (0.01)	0.18 (0.1)	0.11 (0.01)	98.80	3
Jol-9837-02	47.49 (0.35)	2.33 (0.02)	15.15 (0.2)	11.11 (0.19)	0.19 (0.04)	6.92 (0.09)	11.67 (0.03)	2.88 (0.04)	0.41 (0.02)	0.01 (0.01)	0.17 (0.04)	0.13 (0.02)	98.46	3
Jol-9837-03	47.97 (0.08)	2.4 (0.03)	15.24 (0.09)	11.04 (0.33)	0.19 (0.04)	6.91 (0.03)	11.58 (0.13)	2.94 (0.05)	0.39 (0.02)	0.04 (0.01)	0.2 (0.05)	0.11 (0.03)	99.01	3
Jol-9837-04	47.87 (0.27)	2.35 (0.03)	15.11 (0.23)	11.18 (0.28)	0.15 (0.02)	6.94 (0.03)	11.68 (0.15)	2.93 (0.09)	0.39 (0.02)	0.04 (0.01)	0.18 (0.04)	0.13 (0.01)	98.94	3
Jol-9837-05	48.1 (0.05)	2.25 (0.14)	15.36 (0.11)	11.26 (0.22)	0.15 (0.02)	7.04 (0.14)	11.64 (0.11)	3.02 (0.11)	0.42 (0.01)	0.03 (0.01)	0.16 (0.05)	0.11 (0.03)	99.54	3
Jol-9837-06	47.88 (0.06)	2.38 (0.02)	15.31 (0.18)	11.06 (0.25)	0.16 (0.03)	6.97 (0.07)	11.62 (0.12)	2.91 (0.15)	0.42 (0.01)	0.05 (0.01)	0.17 (0.04)	0.12 (0.01)	99.05	3
Jol-9837-07	47.75 (0.13)	2.38 (0.05)	15.17 (0.25)	11.14 (0.05)	0.15 (0.03)	6.98 (0.1)	11.61 (0.1)	2.92 (0.06)	0.42 (0.02)	0.04 (0.01)	0.17 (0.06)	0.12 (0.02)	98.82	3
Jol-9837-08	47.91 (0.16)	2.36 (0.06)	15.25 (0.12)	11.09 (0.42)	0.17 (0.01)	6.74 (0.11)	11.67 (0.2)	2.9 (0.13)	0.39 (0.03)	0.02 (0.02)	0.21 (0.03)	0.12 (0.02)	98.82	3
Jol-9837-09	47.52 (0.28)	2.35 (0.01)	15.24 (0.17)	11.13 (0.5)	0.18 (0.01)	6.86 (0.06)	11.55 (0.11)	2.91 (0.16)	0.41 (0.01)	0.03 (0.03)	0.17 (0.03)	0.14 (0.02)	98.50	3
Jol-9837-10	47.85 (0.11)	2.34 (0.03)	15.24 (0.13)	11.28 (0.35)	0.15 (0.04)	6.96 (0.14)	11.64 (0.17)	2.95 (0.03)	0.39 (0.02)	0.04 (0.01)	0.15 (0.06)	0.13 (0.01)	99.12	3
**Jol-9837**	**47.81 (0.19)**	**2.35 (0.04)**	**15.23 (0.07)**	**11.13 (0.08)**	**0.16 (0.02)**	**6.93 (0.09)**	**11.63 (0.04)**	**2.93 (0.04)**	**0.41 (0.01)**	**0.03 (0.01)**	**0.18 (0.02)**	**0.12 (0.01)**	**98.90**	**30**
Jol-9843-01	47.66 (0.07)	2.39 (0.06)	14.93 (0.15)	11.11 (0.2)	0.2 (0.05)	7.12 (0.12)	12.19 (0.14)	2.67 (0.05)	0.38 (0.02)	0.04 (0.02)	0.18 (0.04)	0.13 (0.02)	98.99	3
Jol-9843-02	47.69 (0.11)	2.36 (0.03)	14.75 (0.07)	11.07 (0.22)	0.16 (0.02)	7 (0.23)	12.11 (0.15)	2.94 (0.22)	0.39 (0.02)	0.03 (0.03)	0.16 (0.08)	0.15 (0.01)	98.81	3
Jol-9843-03	48.02 (0.31)	2.37 (0.05)	14.88 (0.23)	11.41 (0.12)	0.18 (0.02)	7.04 (0.09)	12.06 (0.14)	2.67 (0.03)	0.38 (0.01)	0.02 (0.01)	0.15 (0.08)	0.08 (0.02)	99.27	3
Jol-9843-05	47.64 (0.1)	2.32 (0.04)	14.78 (0.22)	11.15 (0.27)	0.17 (0.03)	7.15 (0.08)	12.14 (0.03)	2.75 (0.13)	0.37 (0.01)	0.04 (0.02)	0.16 (0.05)	0.15 (0.02)	98.79	3
Jol-9843-06	47.76 (0.09)	2.4 (0.01)	14.79 (0.18)	11.31 (0.11)	0.16 (0.01)	7.13 (0.15)	12.07 (0.12)	2.77 (0.08)	0.38 (0.01)	0.04 (0.01)	0.15 (0.07)	0.16 (0.01)	99.11	3
Jol-9843-07	47.74 (0.06)	2.37 (0.04)	14.86 (0.15)	11.2 (0.36)	0.14 (0.02)	7.14 (0.1)	12.14 (0.13)	2.83 (0.07)	0.39 (0.01)	0.03 (0.01)	0.17 (0.06)	0.12 (0.01)	99.12	3
Jol-9843-08	47.5 (0.29)	2.39 (0.02)	14.75 (0.28)	11.26 (0.21)	0.16 (0.02)	7.1 (0.21)	12.18 (0.09)	2.68 (0.05)	0.39 (0.01)	0.02 (0.02)	0.14 (0.05)	0.13 (0.01)	98.71	3
Jol-9843-09	47.68 (0.01)	2.32 (0.05)	14.77 (0.25)	11.48 (0.03)	0.15 (0.01)	7.12 (0.06)	12.14 (0.13)	2.74 (0.09)	0.39 (0.02)	0.03 (0.01)	0.16 (0.06)	0.15 (0.02)	99.14	3
Jol-9843-10	47.48 (0.5)	2.37 (0.06)	14.8 (0.13)	10.84 (0.56)	0.19 (0.01)	7.08 (0.03)	11.96 (0.1)	2.87 (0.3)	0.37 (0.03)	0.06 (0.02)	0.17 (0.05)	0.12 (0.02)	98.30	3
**Jol-9843**	**47.69 (0.16)**	**2.36 (0.03)**	**14.81 (0.06)**	**11.2 (0.19)**	**0.17 (0.02)**	**7.1 (0.05)**	**12.11 (0.07)**	**2.77 (0.09)**	**0.38 (0.01)**	**0.03 (0.01)**	**0.16 (0.01)**	**0.13 (0.02)**	**98.91**	**27**
Jol-9846-01	47.93 (0.15)	2.34 (0.02)	14.9 (0.08)	11.07 (0.51)	0.14 (0.01)	7.1 (0.06)	12.07 (0.15)	2.78 (0.17)	0.37 (0.01)	0.02 (0.02)	0.16 (0.06)	0.13 (0.01)	99.01	3
Jol-9846-02	47.96 (0.19)	2.35 (0.03)	14.85 (0.28)	11.47 (0.37)	0.14 (0.03)	7.09 (0.1)	12 (0.17)	2.79 (0.07)	0.35 (0.04)	0.05 (0.02)	0.17 (0.06)	0.12 (0)	99.34	3
Jol-9846-03	47.77 (0.21)	2.34 (0.03)	14.78 (0.17)	11.03 (0.23)	0.17 (0.03)	7.12 (0.08)	12.09 (0.14)	2.91 (0.15)	0.37 (0.02)	0.02 (0.02)	0.17 (0.04)	0.13 (0.01)	98.91	3
Jol-9846-04	47.99 (0.34)	2.37 (0.03)	14.86 (0.06)	11.35 (0.13)	0.16 (0.02)	7.15 (0.04)	11.97 (0.19)	2.81 (0.17)	0.36 (0.02)	0.03 (0.01)	0.15 (0.07)	0.14 (0)	99.36	3
Jol-9846-05	47.86 (0.23)	2.36 (0.06)	14.88 (0.03)	11.23 (0.35)	0.17 (0.01)	7.1 (0.14)	11.88 (0.15)	2.72 (0.11)	0.36 (0.02)	0.03 (0.02)	0.2 (0.05)	0.13 (0.03)	98.92	3
Jol-9846-06	47.85 (0.23)	2.34 (0.03)	14.88 (0.1)	11.22 (0.41)	0.17 (0.02)	7.1 (0.18)	11.92 (0.22)	2.83 (0.03)	0.35 (0.01)	0.03 (0.01)	0.17 (0.03)	0.12 (0.02)	98.99	3
Jol-9846-08	47.77 (0.23)	2.4 (0.02)	14.74 (0.14)	11.29 (0.12)	0.14 (0.02)	7.09 (0.01)	12.02 (0.09)	2.74 (0.07)	0.37 (0)	0.03 (0.02)	0.16 (0.03)	0.1 (0.01)	98.85	3
Jol-9846-09	47.79 (0.05)	2.34 (0.03)	14.82 (0.11)	11.23 (0.05)	0.16 (0.01)	7.1 (0.08)	11.97 (0.13)	2.71 (0.04)	0.36 (0.02)	0.04 (0.01)	0.16 (0.03)	0.14 (0.01)	98.81	3
Jol-9846-10	47.97 (0.23)	2.37 (0.02)	14.85 (0.24)	11.5 (0.14)	0.15 (0)	7.14 (0.02)	12.04 (0.12)	2.72 (0.08)	0.37 (0.01)	0.04 (0.03)	0.16 (0.05)	0.15 (0)	99.44	3
**Jol-9846**	**47.88 (0.09)**	**2.36 (0.02)**	**14.84 (0.05)**	**11.27 (0.16)**	**0.16 (0.01)**	**7.11 (0.02)**	**12 (0.07)**	**2.78 (0.07)**	**0.36 (0.01)**	**0.03 (0.01)**	**0.17 (0.01)**	**0.13 (0.01)**	**99.07**	**27**
S-19m-02	46.81 (0.11)	2.61 (0.03)	16.54 (0.11)	11.79 (0.19)	0.19 (0.02)	5.89 (0.01)	9.91 (0.03)	3.93 (0.23)	0.65 (0.01)	0.01 (0.02)	0.21 (0.03)	0.1 (0.01)	98.63	3
S-19m-04	47.01 (0.2)	2.59 (0.02)	16.62 (0.04)	11.69 (0.27)	0.17 (0.02)	5.88 (0.02)	9.8 (0.1)	3.89 (0.03)	0.67 (0.03)	0.02 (0.01)	0.23 (0.03)	0.11 (0.01)	98.66	3
S-19m-05	46.68 (0.02)	2.72 (0.04)	16.02 (0.05)	12.46 (0.05)	0.18 (0.03)	6.07 (0.04)	9.61 (0.07)	3.9 (0.15)	0.72 (0.03)	0.02 (0.03)	0.25 (0.02)	0.11 (0.02)	98.72	3
S-19m-06	46.63 (0.14)	2.67 (0.04)	16.57 (0.03)	12.31 (0.02)	0.19 (0.02)	6.14 (0.04)	9.71 (0.14)	3.78 (0.1)	0.63 (0.03)	0.02 (0.01)	0.22 (0)	0.11 (0.01)	99.00	3
S-19m-07	46.97 (0.19)	2.6 (0.05)	16.55 (0.19)	11.68 (0.17)	0.19 (0.01)	5.87 (0.04)	9.81 (0.05)	3.79 (0.08)	0.67 (0.01)	0.01 (0.01)	0.27 (0.01)	0.1 (0.03)	98.50	3
S-19m-08	46.64 (0.1)	2.58 (0)	16.52 (0.11)	11.81 (0.02)	0.16 (0.01)	5.88 (0.01)	9.85 (0.02)	3.83 (0.22)	0.66 (0)	0.02 (0.02)	0.28 (0.02)	0.11 (0)	98.34	2
S-19m-09	46.46 (0.2)	2.69 (0.03)	16.38 (0.05)	12.4 (0.18)	0.19 (0.02)	6.01 (0.13)	9.77 (0.02)	3.74 (0.32)	0.67 (0.01)	0.02 (0.02)	0.24 (0.03)	0.09 (0.01)	98.66	3
S-19m-10	46.55 (0.26)	2.63 (0.03)	16.43 (0.23)	12 (0.1)	0.2 (0.04)	5.82 (0.06)	9.87 (0.12)	3.83 (0.22)	0.64 (0.03)	0.01 (0.01)	0.25 (0.02)	0.1 (0.01)	98.32	3
**S-19m**	**46.72 (0.19)**	**2.63 (0.05)**	**16.45 (0.19)**	**12.02 (0.32)**	**0.18 (0.01)**	**5.94 (0.12)**	**9.79 (0.1)**	**3.83 (0.07)**	**0.67 (0.03)**	**0.02 (0.01)**	**0.24 (0.02)**	**0.1 (0.01)**	**98.61**	**23**
S-48m-02	47.04 (0.25)	2.63 (0.02)	16.04 (0.06)	12 (0.36)	0.18 (0.02)	5.95 (0.05)	9.58 (0.02)	3.72 (0.08)	0.62 (0.02)	0.03 (0.03)	0.28 (0.01)	0.09 (0.02)	98.17	3
S-48m-03	47.07 (0.14)	2.61 (0.03)	16.04 (0.09)	11.96 (0.54)	0.2 (0.02)	5.98 (0.19)	9.65 (0.05)	3.83 (0.12)	0.64 (0.01)	0 (0)	0.28 (0.03)	0.09 (0.01)	98.34	3
S-48m-04	46.78 (0.26)	2.63 (0.1)	16.07 (0.25)	12.3 (0.19)	0.19 (0.03)	6.1 (0.01)	9.57 (0.03)	3.68 (0.1)	0.69 (0)	0.03 (0.01)	0.31 (0.01)	0.1 (0.02)	98.44	3
S-48m-05	47.04 (0.05)	2.63 (0.06)	16.25 (0.11)	12.03 (0.41)	0.21 (0.04)	5.9 (0.18)	9.67 (0.05)	3.74 (0.04)	0.62 (0.02)	0.02 (0)	0.29 (0.02)	0.1 (0.01)	98.50	3
S-48m-06	47.23 (0.1)	2.65 (0.02)	16.24 (0.05)	12.18 (0.12)	0.19 (0.01)	6.05 (0.11)	9.64 (0.15)	3.83 (0.05)	0.68 (0.02)	0.01 (0)	0.28 (0.01)	0.09 (0.02)	99.07	3
S-48m-07	46.54 (0.22)	2.87 (0.03)	16.47 (0.06)	13.09 (0.41)	0.19 (0.01)	5.55 (0.04)	9.22 (0.11)	3.9 (0.22)	0.64 (0.02)	0 (0.01)	0.29 (0.03)	0.08 (0.02)	98.86	3
S-48m-08	47.18 (0.15)	2.66 (0.03)	16.24 (0.06)	12.1 (0.11)	0.19 (0.04)	6.08 (0.01)	9.67 (0.04)	3.82 (0.06)	0.66 (0.01)	0 (0)	0.24 (0.03)	0.1 (0.02)	98.93	3
S-48m-09	47.13 (0.07)	2.66 (0.01)	16.16 (0.03)	12.14 (0.14)	0.19 (0.02)	5.99 (0.1)	9.59 (0.13)	3.91 (0.04)	0.65 (0.02)	0 (0)	0.27 (0.01)	0.1 (0)	98.79	3
S-48m-10	47.12 (0.07)	2.66 (0.07)	16.13 (0.08)	12.1 (0.13)	0.2 (0.01)	6.09 (0.05)	9.71 (0.03)	3.81 (0.04)	0.65 (0.02)	0.02 (0.01)	0.25 (0.02)	0.1 (0.01)	98.85	3
S-48m-11	47.15 (0.17)	2.6 (0.1)	16.34 (0.11)	11.96 (0.43)	0.18 (0.02)	6.11 (0.03)	9.71 (0.07)	3.76 (0.09)	0.65 (0.02)	0.01 (0.01)	0.23 (0.02)	0.08 (0.01)	98.79	3
**S-48m**	**47.03 (0.21)**	**2.66 (0.08)**	**16.2 (0.14)**	**12.18 (0.34)**	**0.19 (0.01)**	**5.98 (0.17)**	**9.6 (0.14)**	**3.8 (0.08)**	**0.65 (0.02)**	**0.01 (0.01)**	**0.27 (0.02)**	**0.09 (0.01)**	**98.67**	**30**

S-9822-04	33.57 (0.93)	299.19 (5.04)	42.9 (1.03)	58.79 (1.98)	75.61 (2.05)	96.04 (7.85)	23.42 (0.77)	1.35 (0.45)	14.16 (0.37)	332.95 (4.79)	35.05 (0.68)	184.88 (3.03)	22.38 (0.38)	157.29 (3.15)	16.82 (0.34)	41.87 (0.71)	5.45 (0.15)
S-9822-07	33.13 (0.67)	295.51 (4.64)	43.1 (0.98)	57.6 (1.68)	66.12 (1.83)	91.03 (3.5)	22.3 (0.54)	1.31 (0.46)	15.02 (0.4)	333.98 (4.47)	37.37 (0.62)	201.49 (3.05)	23.46 (0.36)	163.35 (3.41)	18.56 (0.34)	44.6 (0.71)	5.92 (0.15)
S-9822-09	31.01 (0.98)	285.45 (5.19)	45 (1.01)	64.92 (2.33)	76.56 (2.1)	94.55 (3.22)	23.62 (0.75)		13.85 (0.47)	333.59 (4.46)	33.62 (0.63)	176 (3.1)	21.24 (0.41)	151.69 (3.31)	16.96 (0.42)	39.54 (0.67)	5.24 (0.13)
**S-9822**	**32.57 (1.24)**	**293.39 (6.93)**	**43.67 (1.15)**	**60.44 (3.77)**	**72.77 (5.17)**	**93.87 (3.23)**	**23.11 (0.7)**		**14.34 (0.63)**	**333.51 (0.48)**	**35.35 (1.7)**	**187.46 (11.75)**	**22.36 (0.81)**	**157.44 (5.06)**	**17.45 (0.9)**	**42 (2.26)**	**5.54 (0.31)**
S-9821-05	31.48 (0.99)	268.97 (8.11)	38.89 (1.46)	48.18 (2.08)	61.8 (2.42)	91.74 (7.38)	23.6 (1.36)		16.51 (0.75)	344.01 (7.77)	36.56 (1.06)	218.2 (6.08)	26.42 (0.8)	179.63 (6.33)	21.31 (0.78)	48.84 (1.28)	7.05 (0.26)
S-9821-07	29.91 (0.74)	272.97 (3.96)	42.24 (0.87)	66 (2.04)	59.59 (1.83)	93.19 (3.79)	23.55 (0.52)		14.41 (0.39)	359.62 (4.83)	35.69 (0.61)	202.33 (2.88)	23.61 (0.34)	166.93 (3.16)	18.62 (0.35)	45.11 (0.67)	6.07 (0.13)
S-9821-09	30.78 (0.7)	286.07 (4.41)	42.22 (0.8)	59.72 (1.78)	64.61 (1.77)	106.56 (11.13)	22.83 (0.53)	1.8 (0.45)	15.26 (0.42)	339.44 (4.34)	37.62 (0.62)	209.43 (2.87)	25.24 (0.38)	173.47 (3.41)	19.55 (0.36)	46.55 (0.69)	6.13 (0.15)
**S-9821**	**30.72 (0.71)**	**276 (8.71)**	**41.12 (1.91)**	**57.97 (8.68)**	**62 (2.25)**	**97.16 (10.24)**	**23.33 (0.42)**		**15.39 (1.09)**	**347.69 (9.83)**	**36.63 (0.87)**	**209.99 (7.22)**	**25.09 (1.03)**	**173.34 (5.51)**	**19.83 (1.27)**	**46.83 (1.68)**	**6.42 (0.49)**
Syrt-9832-02	35.59 (1.05)	310.78 (5.43)	45.17 (1.08)	97.34 (3.1)	68.07 (2.58)	78.72 (2.9)	20.39 (0.72)		8.3 (0.39)	293.88 (4.65)	25.81 (0.52)	126.87 (2.26)	13.74 (0.28)	93.24 (2.54)	11.11 (0.25)	27.73 (0.53)	4 (0.12)
Syrt-9832-05	34.75 (0.74)	322.17 (5.02)	47.23 (0.99)	104.38 (2.55)	104.47 (2.05)	82.44 (2.77)	22.48 (0.56)	1.19 (0.4)	8.06 (0.27)	303.75 (4.22)	26.37 (0.5)	126.65 (2.09)	14.23 (0.24)	96.31 (1.94)	11.36 (0.25)	28.33 (0.42)	3.81 (0.09)
Syrt-9832-08	36.27 (0.9)	313.98 (5.65)	46.08 (1.15)	94.85 (2.94)	99.29 (2.64)	78.63 (2.82)	21.02 (0.62)	1.97 (0.55)	7.97 (0.29)	305.53 (4.75)	26.26 (0.48)	130.37 (2.41)	14.03 (0.3)	100.3 (2.5)	11.01 (0.27)	27.7 (0.49)	3.72 (0.1)
**Syrt-9832**	**35.54 (0.69)**	**315.64 (5.72)**	**46.16 (1.02)**	**98.86 (4.75)**	**90.61 (17.64)**	**79.93 (2.72)**	**21.3 (1.05)**		**8.11 (0.18)**	**301.05 (5.83)**	**26.14 (0.27)**	**127.96 (1.89)**	**14 (0.18)**	**96.62 (3.07)**	**11.16 (0.17)**	**27.92 (0.32)**	**3.84 (0.13)**
Syrt-9833-01	35.95 (1.07)	318.86 (5.77)	47.04 (1.08)	96.85 (3.03)	101.17 (4.03)	81.53 (3.33)	21.34 (0.67)		7.84 (0.26)	307.62 (4.79)	26.17 (0.51)	130.49 (2.4)	14.1 (0.28)	96.19 (2.38)	11.47 (0.26)	27.99 (0.49)	3.75 (0.11)
Syrt-9833-05	38.55 (1.23)	318.04 (6.68)	47.24 (1.36)	95.22 (4.17)	93.28 (3.25)	87.97 (4.21)	22.1 (0.91)		7.97 (0.67)	302.69 (5.35)	25.85 (0.7)	127.17 (3.64)	14.45 (0.37)	95.96 (3.1)	11.33 (0.38)	28.18 (0.82)	3.74 (0.2)
Syrt-9833-07	34.58 (0.8)	320.01 (5.06)	45.02 (0.9)	101.08 (2.55)	98.6 (2.04)	156.97 (90.27)	21 (0.54)	0.94 (0.41)	8.03 (0.25)	305.8 (4.37)	27.22 (0.46)	131.76 (1.92)	14.15 (0.26)	98.5 (1.94)	11.42 (0.24)	28.33 (0.46)	3.86 (0.1)
**Syrt-9833**	**36.36 (1.83)**	**318.97 (0.96)**	**46.43 (1.22)**	**97.71 (2.9)**	**97.68 (3.6)**	**108.83 (52.44)**	**21.48 (0.55)**		**7.95 (0.1)**	**305.37 (2.31)**	**26.41 (0.64)**	**129.8 (2.15)**	**14.23 (0.14)**	**96.88 (1.22)**	**11.41 (0.06)**	**28.17 (0.15)**	**3.78 (0.06)**
Syrt-11221-03	34.9 (1.18)	309.98 (6.07)	44.1 (1.31)	99.47 (3.1)	91.14 (2.7)	77.3 (3.29)	21.29 (0.78)		7.56 (0.3)	301.43 (5.03)	26.24 (0.55)	130.71 (2.42)	14.05 (0.3)	96.6 (2.33)	11.33 (0.32)	27.95 (0.58)	3.89 (0.11)
Syrt-11221-05	36.32 (1.11)	313.91 (5.62)	44.86 (1.15)	92.21 (2.99)	98.44 (2.41)	76.91 (3.25)	21.06 (0.68)	1.36 (0.43)	7.88 (0.33)	303.87 (4.7)	26.92 (0.55)	130.45 (2.32)	13.93 (0.28)	95.66 (1.99)	11.41 (0.27)	28 (0.5)	3.87 (0.12)
Syrt-11221-07	35.97 (0.73)	315.8 (4.72)	46.7 (0.99)	100.23 (2.59)	94.52 (2.08)	93.4 (14.47)	22.44 (0.6)		7.92 (0.22)	313.06 (4.25)	27.53 (0.45)	133.25 (1.89)	14.58 (0.27)	103.07 (1.98)	11.34 (0.21)	28.98 (0.42)	4 (0.12)
**Syrt-11221**	**35.73 (0.67)**	**313.23 (2.89)**	**45.22 (1.32)**	**97.3 (4.26)**	**94.7 (3.27)**	**82.53 (11.8)**	**21.6 (0.73)**		**7.79 (0.2)**	**306.12 (5.7)**	**26.9 (0.58)**	**131.47 (1.4)**	**14.19 (0.26)**	**98.44 (3.5)**	**11.36 (0.04)**	**28.31 (0.52)**	**3.92 (0.06)**
Syrt-9834-01	37.89 (0.9)	318.16 (5.68)	43.97 (1.1)	95.91 (3.02)	95.72 (2.98)	82.26 (4.78)	21.61 (0.77)	1.55 (0.58)	8.59 (0.33)	315.87 (5.01)	27.66 (0.59)	135.98 (2.26)	15.02 (0.32)	103.75 (2.69)	11.7 (0.3)	28.82 (0.52)	3.94 (0.14)
Syrt-9834-07	36.05 (1.08)	323.57 (5.99)	46.14 (1.2)	98.6 (3.25)	100.38 (3.13)	85.49 (3.24)	22.36 (0.81)	2.32 (0.64)	8.45 (0.32)	313.69 (5.1)	27.85 (0.58)	135.87 (2.63)	15.01 (0.36)	103.23 (2.38)	11.78 (0.28)	30.38 (0.6)	4.12 (0.13)
Syrt-9834-10	35.79 (0.8)	320.09 (4.93)	45.83 (0.96)	97.65 (2.93)	131.66 (10)	84.29 (2.72)	21.87 (0.63)	1.91 (0.45)	8.42 (0.29)	315.12 (4.49)	28.26 (0.45)	136.86 (2.13)	14.92 (0.23)	103.78 (1.96)	11.82 (0.22)	29.8 (0.47)	4.09 (0.1)
	0.17	2.39	0.21	0.65	19.81	1.06	0.34		0.02	0.94	0.26	0.64	0.04	0.34	0.02	0.36	0.02
Syrt-9831-04	35.06 (0.85)	320.39 (5.05)	45.07 (0.96)	102.01 (2.53)	99.83 (1.92)	79.2 (2.62)	20.47 (0.55)		7.97 (0.27)	307.65 (4.22)	27.6 (0.51)	132.34 (1.97)	14.76 (0.25)	98.01 (1.97)	11.86 (0.25)	28.81 (0.46)	3.87 (0.09)
Syrt-9831-07	35.94 (1)	319.48 (6.02)	46.46 (1.1)	101.93 (3.47)	93.18 (2.54)	82.4 (3.8)	22.54 (0.76)		7.77 (0.28)	308.65 (4.85)	26.94 (0.55)	132.35 (2.35)	14.06 (0.31)	93.36 (2.45)	11.19 (0.29)	28.14 (0.57)	3.86 (0.13)
Syrt-9831-09	36.12 (0.96)	315.93 (5.82)	45.98 (1.01)	98.5 (3.17)	90.22 (2.68)	88.55 (9.96)	21.57 (0.69)	1.93 (0.57)	8.68 (0.28)	301.74 (4.75)	26.05 (0.51)	125.32 (2.34)	14.3 (0.27)	97.16 (2.49)	10.75 (0.27)	27.31 (0.48)	3.58 (0.1)
**Syrt-9831**	**35.71 (0.52)**	**318.6 (2.29)**	**45.84 (0.7)**	**100.81 (1.92)**	**94.41 (4.41)**	**83.38 (5.96)**	**21.53 (1.01)**		**8.14 (0.49)**	**306.01 (3.46)**	**26.86 (0.7)**	**130 (3.68)**	**14.37 (0.26)**	**96.18 (2.15)**	**11.27 (0.52)**	**28.08 (0.67)**	**3.77 (0.15)**
Syrt-9835-02	36.81 (1)	324.9 (5.94)	45.26 (1.27)	94.08 (3.83)	106.88 (6.03)	93.28 (9.85)	22.03 (0.65)	1.8 (0.45)	7.57 (0.34)	313.79 (5)	29.09 (0.65)	137.91 (2.61)	14.88 (0.33)	105.57 (2.34)	11.71 (0.28)	29.62 (0.59)	3.82 (0.11)
Syrt-9835-05	38.65 (1.1)	333.27 (6.65)	46.86 (1.15)	94.76 (3.13)	95.05 (2.85)	83.23 (3.4)	21.15 (0.68)	1.16 (0.56)	8.26 (0.43)	322.23 (5.45)	28.88 (0.64)	140.16 (2.51)	15.36 (0.33)	103.26 (2.29)	11.73 (0.27)	29.67 (0.61)	4.09 (0.14)
Syrt-9835-08	36.65 (0.84)	318.96 (5.14)	45.97 (0.95)	96.54 (2.54)	94.87 (2.17)	83.18 (2.71)	21.34 (0.66)		8.1 (0.26)	314.76 (4.55)	27.61 (0.48)	135.43 (2.18)	14.8 (0.25)	101.98 (2.08)	11.89 (0.25)	29.16 (0.52)	3.95 (0.09)
**Syrt-9835**	**37.37 (1.01)**	**325.71 (7)**	**46.03 (0.79)**	**95.13 (1.22)**	**98.93 (6.16)**	**86.57 (7.29)**	**21.51 (0.45)**		**7.97 (0.37)**	**316.92 (4.29)**	**28.53 (0.72)**	**137.83 (2.15)**	**15.01 (0.22)**	**103.6 (1.58)**	**11.77 (0.09)**	**29.49 (0.25)**	**3.95 (0.12)**
Jol-11222-02	33.19 (0.98)	308.02 (5.48)	45.94 (1.24)	109.03 (3.56)	142.41 (5.12)	81.65 (22.66)	21.46 (0.77)		7.3 (0.31)	300.77 (5.81)	27.07 (0.65)	127.08 (2.62)	13.07 (0.33)	89.54 (2.51)	11.05 (0.29)	26.13 (0.55)	3.93 (0.12)
Jol-11222-07	33.95 (4.11)	374.74 (23.1)	40.13 (4.17)	72.43 (5.45)	88.1 (9.1)	88.08 (5.98)	17.77 (1.12)		7.28 (0.49)	305.35 (14.45)	23.16 (1.48)	125.19 (5.95)	14.41 (1.39)	102.11 (3.96)	12.24 (0.97)	29.88 (2.16)	4.02 (0.33)
Jol-11222-08	36.43 (0.97)	323.22 (5.79)	44.58 (1.02)	98.1 (3.09)	97.27 (3.78)	83.88 (3.47)	21.51 (0.67)		7.02 (0.3)	308.47 (5.16)	27.52 (0.53)	129.73 (2.33)	14.2 (0.3)	92.62 (2.24)	11 (0.25)	27.55 (0.53)	3.95 (0.1)
**Jol-11222**	**34.52 (1.54)**	**335.33 (34.03)**	**43.55 (3.01)**	**93.19 (18.05)**	**109.26 (26.04)**	**84.53 (4.09)**	**20.25 (2.1)**		**7.2 (0.16)**	**304.86 (3.6)**	**25.92 (2.15)**	**127.33 (2.07)**	**13.89 (0.53)**	**94.76 (5.69)**	**11.43 (0.65)**	**27.86 (1.69)**	**3.97 (0.04)**
Jol-9836-01	34.52 (0.98)	319.29 (6.01)	46.97 (1.21)	99.35 (3.39)	96.36 (3.31)	88.37 (3.89)	22.53 (0.82)	1.67 (0.56)	7.05 (0.28)	307.37 (5.08)	27.44 (0.54)	132.14 (2.39)	13.51 (0.27)	94.43 (2.22)	10.75 (0.3)	27.15 (0.55)	3.86 (0.1)
Jol-9836-02	36.49 (1.53)	323.54 (7.45)	44.22 (1.57)	91.14 (3.4)	91.9 (3.78)	86.92 (5.23)	21.41 (1.18)		7.39 (0.55)	300.61 (6.28)	27.41 (0.69)	128.95 (2.96)	13.33 (0.4)	86.89 (3.37)	10.82 (0.33)	26.87 (0.51)	3.91 (0.17)
Jol-9836-03	35.82 (0.79)	325.52 (5.15)	45.54 (0.76)	101.02 (2.6)	93.7 (2.09)	82.02 (2.89)	21.78 (0.53)	1.79 (0.44)	7.33 (0.28)	300.79 (4.48)	27.71 (0.5)	127.61 (2.01)	13.63 (0.2)	91.3 (2.01)	10.69 (0.23)	26.56 (0.4)	3.61 (0.11)
**Jol-9836**	**35.61 (0.91)**	**322.78 (3.1)**	**45.57 (1.36)**	**97.17 (5.08)**	**93.98 (2.01)**	**85.77 (4.17)**	**21.91 (0.56)**		**7.25 (0.19)**	**302.92 (3.58)**	**27.52 (0.15)**	**129.57 (2.11)**	**13.49 (0.11)**	**90.87 (3.28)**	**10.75 (0.06)**	**26.86 (0.26)**	**3.8 (0.14)**
Jol-9837-02	34.76 (0.95)	320.74 (6.22)	45.49 (1.05)	98.95 (3.01)	92.79 (2.42)	79.55 (3.04)	22.7 (0.87)		7.43 (0.28)	304.71 (4.81)	28.02 (0.57)	131.89 (2.28)	13.77 (0.31)	91.98 (2.15)	11.21 (0.26)	27.2 (0.55)	3.68 (0.1)
Jol-9837-05	37.11 (0.91)	323.92 (4.84)	43.94 (0.94)	97.92 (2.24)	82.38 (2.19)	81.47 (2.95)	20.98 (0.6)	1.17 (0.39)	7.15 (0.22)	303.2 (4.07)	28.15 (0.46)	131.22 (1.95)	13.79 (0.23)	92.62 (1.87)	10.98 (0.23)	27.21 (0.5)	3.7 (0.09)
Jol-9837-07	35.26 (1.1)	319.94 (5.86)	44.96 (1.13)	99.28 (3.41)	80.05 (2.75)	85.21 (3.22)	21.83 (0.72)	1.15 (0.48)	7.17 (0.34)	303.3 (4.94)	28.2 (0.68)	132.64 (2.41)	14.13 (0.34)	92.41 (2.25)	10.54 (0.27)	26.44 (0.53)	3.73 (0.13)
**Jol-9837**	**35.71 (1.12)**	**321.53 (2.05)**	**44.8 (0.78)**	**98.72 (0.68)**	**85.07 (6.07)**	**82.08 (3.61)**	**21.84 (0.84)**		**7.25 (0.16)**	**303.74 (0.78)**	**28.12 (0.08)**	**131.92 (0.64)**	**13.9 (0.15)**	**92.34 (0.28)**	**10.91 (0.32)**	**26.95 (0.39)**	**3.7 (0.02)**
Jol-9843-02	36.44 (0.85)	330.67 (4.71)	47.96 (0.89)	97.19 (2.52)	89.1 (2.26)	82.83 (2.46)	21.33 (0.59)	1.53 (0.44)	6.65 (0.23)	301.39 (4.23)	26.27 (0.42)	122.69 (1.92)	12.8 (0.24)	86.53 (1.89)	10 (0.2)	25.77 (0.42)	3.54 (0.11)
Jol-9843-05	37.36 (1.02)	337.76 (6.34)	47.35 (1.17)	102.13 (3.71)	95.57 (2.61)	85.73 (3.4)	22.49 (0.64)	1.85 (0.66)	6.5 (0.26)	305.7 (5.03)	27.04 (0.57)	123.95 (2.46)	13.02 (0.3)	86.3 (2.01)	10.05 (0.23)	25.61 (0.55)	3.65 (0.11)
Jol-9843-08	36.69 (1.02)	335.7 (6.49)	46.65 (1.12)	104.18 (2.83)	115.23 (4)	87.68 (3.37)	22.83 (0.78)		7.05 (0.29)	296.06 (5)	26.06 (0.56)	120.35 (2.21)	12.62 (0.26)	84.02 (2.34)	10.04 (0.23)	25.25 (0.48)	3.6 (0.12)
**Jol-9843**	**36.83 (0.43)**	**334.71 (3.55)**	**47.32 (0.65)**	**101.17 (3.45)**	**99.97 (12.19)**	**85.41 (3.06)**	**22.22 (0.77)**		**6.73 (0.29)**	**301.05 (4.49)**	**26.45 (0.46)**	**122.33 (1.66)**	**12.81 (0.15)**	**85.62 (1.2)**	**10.03 (0.03)**	**25.54 (0.24)**	**3.6 (0.05)**
Jol-9846-03	37.94 (1.09)	334.49 (6.32)	47.66 (1.27)	100.37 (3.21)	96.98 (2.95)	87.57 (4.24)	22.49 (0.74)	1.01 (0.57)	6.63 (0.27)	299.2 (4.78)	27.1 (0.57)	126.52 (2.36)	12.73 (0.25)	79.93 (1.92)	9.8 (0.23)	25.28 (0.49)	3.5 (0.12)
Jol-9846-09	36.84 (1.15)	333.71 (6.29)	45.23 (1.21)	98.14 (2.9)	95.29 (2.81)	81.15 (3.24)	21.79 (0.75)		6.21 (0.28)	295.65 (4.81)	27.17 (0.56)	124.5 (2.43)	12.71 (0.28)	78.57 (2.12)	9.74 (0.25)	24.98 (0.49)	3.44 (0.11)
Jol-9846-10	37.41 (0.93)	337.89 (5.48)	46.51 (0.87)	102.15 (2.53)	95.96 (2.14)	84.37 (2.77)	20.83 (0.53)		6.02 (0.22)	300.26 (4.37)	27.22 (0.43)	123.82 (2)	12.58 (0.28)	83.74 (2.02)	9.88 (0.23)	25.19 (0.4)	3.66 (0.09)
**Jol-9846**	**37.4 (0.5)**	**335.36 (2.17)**	**46.47 (1.2)**	**100.22 (1.93)**	**96.08 (0.76)**	**84.36 (4.03)**	**21.7 (0.82)**		**6.29 (0.32)**	**298.37 (2.24)**	**27.16 (0.05)**	**124.95 (1.28)**	**12.67 (0.06)**	**80.75 (2.32)**	**9.81 (0.06)**	**25.15 (0.14)**	**3.53 (0.1)**
S-19m-06	29.86 (0.77)	281.57 (4.1)	43.24 (1.11)	63.15 (2.18)	51.8 (1.51)	104.35 (16.02)	22.63 (0.63)		13.51 (0.41)	363.54 (4.83)	35.33 (0.55)	195.04 (2.84)	23.78 (0.4)	166.36 (3.33)	18.62 (0.38)	43.31 (0.67)	5.9 (0.13)
S-19m-09	29.85 (0.91)	283.61 (4.31)	44.89 (0.98)	70.69 (2.35)	63.86 (1.95)	105.82 (8.32)	24.84 (0.78)	1.88 (0.46)	14.01 (0.41)	367.82 (5.37)	34.07 (0.58)	193.26 (3.36)	23.36 (0.39)	151.52 (3.43)	18.32 (0.36)	43.13 (0.69)	5.88 (0.16)
S-19m-10	29.81 (0.76)	277.22 (4.56)	42.71 (0.97)	58.68 (2.02)	65.9 (1.95)	106.1 (4.76)	23.02 (0.75)	2.42 (0.59)	15.96 (0.42)	362.22 (5.06)	34.57 (0.61)	197.74 (3.04)	23.46 (0.36)	173.18 (3.48)	18.26 (0.35)	44.02 (0.75)	6.11 (0.16)
**S-19m**	**29.84 (0.02)**	**280.8 (3.18)**	**43.61 (1.13)**	**64.18 (5.83)**	**60.52 (6.83)**	**105.42 (1.18)**	**23.5 (1.16)**		**14.5 (1.33)**	**364.53 (2.72)**	**34.65 (0.57)**	**195.35 (2.05)**	**23.54 (0.16)**	**163.69 (9.61)**	**18.4 (0.18)**	**43.49 (0.42)**	**5.96 (0.11)**
S-48m-04	25.08 (0.82)	241.79 (7.18)	36.04 (1.04)	50.14 (2.06)	42.05 (2.37)	76.83 (3.97)	23.73 (0.6)		12.46 (0.49)	412.32 (6.93)	30.61 (0.71)	176.65 (3.92)	21.87 (0.51)	172.27 (4)	16.59 (0.5)	40.16 (1.15)	5.27 (0.17)
S-48m-05	28.62 (0.69)	277.35 (4.57)	42.22 (0.85)	62.71 (2.2)	64.24 (2.08)	93.64 (3.46)	23.98 (0.72)		15.14 (0.47)	351.64 (4.97)	35.2 (0.6)	204.08 (2.95)	24.55 (0.38)	174.99 (3.22)	19.24 (0.34)	45.66 (0.76)	5.93 (0.13)
S-48m-11	29.58 (0.75)	274.1 (4.29)	42.83 (0.96)	63.26 (2.24)	62.26 (1.53)	91.86 (3.2)	23.46 (0.75)		15.71 (0.41)	359.94 (4.95)	37.14 (0.54)	206.18 (3.14)	24.22 (0.39)	181.38 (3.32)	19.77 (0.36)	46.47 (0.7)	6.41 (0.15)
**S-48m**	**27.76 (2.15)**	**264.41 (19.13)**	**40.37 (3.72)**	**58.7 (7.13)**	**56.19 (11)**	**87.45 (11.58)**	**23.72 (0.25)**		**14.43 (1.79)**	**374.63 (30.55)**	**34.32 (3.01)**	**195.64 (14.96)**	**23.55 (1.07)**	**176.21 (4.05)**	**18.53 (1.59)**	**44.1 (3.06)**	**5.87 (0.5)**

S-9822-04	23.66 (0.76)	6.46 (0.37)	2.08 (0.08)	6.89 (0.38)	1.06 (0.06)	6.49 (0.28)	1.23 (0.05)	3.42 (0.14)	0.5 (0.04)	2.77 (0.19)	0.49 (0.03)	4.21 (0.22)	1.48 (0.04)	1.56 (0.1)	1.37 (0.06)	0.44 (0.04)
S-9822-07	28.52 (0.76)	6.7 (0.32)	2.37 (0.11)	7.72 (0.4)	1.1 (0.04)	7.37 (0.27)	1.33 (0.06)	3.75 (0.18)	0.57 (0.03)	3.29 (0.2)	0.53 (0.03)	5.09 (0.2)	1.59 (0.04)	1.59 (0.1)	1.64 (0.08)	0.5 (0.04)
S-9822-09	23.21 (0.69)	5.48 (0.36)	2.03 (0.08)	6.68 (0.29)	0.91 (0.05)	6.1 (0.26)	1.12 (0.06)	3.68 (0.16)	0.46 (0.03)	3.04 (0.22)	0.45 (0.03)	3.85 (0.19)	1.28 (0.05)	1.78 (0.1)	1.39 (0.08)	0.49 (0.04)
**S-9822**	**25.13 (2.68)**	**6.21 (0.61)**	**2.16 (0.17)**	**7.1 (0.49)**	**1.02 (0.09)**	**6.65 (0.61)**	**1.22 (0.1)**	**3.62 (0.16)**	**0.51 (0.05)**	**3.03 (0.25)**	**0.49 (0.04)**	**4.38 (0.59)**	**1.45 (0.11)**	**1.64 (0.12)**	**1.46 (0.15)**	**0.48 (0.03)**
S-9821-05	29.56 (1.36)	6.77 (0.48)	2.11 (0.17)	7.01 (0.55)	1.23 (0.08)	7.8 (0.48)	1.46 (0.09)	4.27 (0.28)	0.4 (0.06)	3.81 (0.37)	0.54 (0.06)	4.41 (0.33)	1.68 (0.08)	2.06 (0.15)	1.55 (0.13)	0.42 (0.05)
S-9821-07	27.28 (0.86)	6.46 (0.34)	2.44 (0.1)	6.6 (0.29)	1.13 (0.05)	6.82 (0.26)	1.34 (0.05)	3.48 (0.18)	0.51 (0.03)	3.07 (0.17)	0.44 (0.03)	4.76 (0.21)	1.51 (0.04)	1.62 (0.12)	1.57 (0.08)	0.53 (0.04)
S-9821-09	27.27 (0.62)	6.98 (0.34)	2.11 (0.1)	7.38 (0.34)	1.12 (0.05)	7.58 (0.26)	1.35 (0.05)	3.76 (0.15)	0.54 (0.04)	3.21 (0.2)	0.5 (0.04)	5.17 (0.26)	1.58 (0.04)	1.75 (0.1)	1.74 (0.07)	0.53 (0.04)
**S-9821**	**28.04 (1.2)**	**6.74 (0.24)**	**2.22 (0.17)**	**6.99 (0.35)**	**1.16 (0.06)**	**7.4 (0.48)**	**1.38 (0.06)**	**3.84 (0.36)**	**0.48 (0.06)**	**3.36 (0.38)**	**0.49 (0.04)**	**4.78 (0.35)**	**1.59 (0.06)**	**1.81 (0.23)**	**1.62 (0.1)**	**0.49 (0.06)**
Syrt-9832-02	17.93 (0.58)	4.78 (0.26)	1.51 (0.08)	5.37 (0.3)	0.75 (0.04)	5.08 (0.24)	1.06 (0.05)	2.61 (0.13)	0.34 (0.02)	2.31 (0.17)	0.39 (0.03)	3.17 (0.2)	0.93 (0.04)	0.96 (0.08)	0.95 (0.06)	0.29 (0.03)
Syrt-9832-05	17.05 (0.52)	5.15 (0.26)	1.48 (0.07)	5.3 (0.29)	0.72 (0.04)	5.14 (0.2)	1 (0.04)	2.64 (0.15)	0.4 (0.03)	2.42 (0.15)	0.36 (0.03)	3.21 (0.16)	0.93 (0.03)	1.09 (0.08)	0.97 (0.05)	0.33 (0.03)
Syrt-9832-08	18.23 (0.62)	5.22 (0.33)	1.7 (0.09)	5.57 (0.31)	0.82 (0.04)	5.36 (0.23)	0.96 (0.04)	2.53 (0.12)	0.34 (0.02)	2.47 (0.14)	0.31 (0.03)	3.24 (0.19)	0.87 (0.03)	1.04 (0.07)	0.94 (0.05)	0.35 (0.03)
**Syrt-9832**	**17.74 (0.56)**	**5.05 (0.22)**	**1.56 (0.11)**	**5.41 (0.13)**	**0.76 (0.05)**	**5.19 (0.14)**	**1 (0.05)**	**2.59 (0.05)**	**0.36 (0.03)**	**2.4 (0.08)**	**0.35 (0.04)**	**3.21 (0.03)**	**0.91 (0.02)**	**1.03 (0.07)**	**0.95 (0.01)**	**0.32 (0.03)**
Syrt-9833-01	18.53 (0.57)	5.12 (0.3)	1.62 (0.08)	5.69 (0.36)	0.79 (0.05)	5.21 (0.22)	1 (0.05)	2.94 (0.18)	0.41 (0.03)	2.06 (0.16)	0.39 (0.03)	3.51 (0.2)	0.8 (0.03)	1.12 (0.08)	0.92 (0.05)	0.3 (0.03)
Syrt-9833-05	18.2 (0.87)	4.58 (0.35)	1.57 (0.09)	4.79 (0.4)	0.84 (0.06)	5.45 (0.4)	0.96 (0.08)	2.95 (0.19)	0.36 (0.03)	2.7 (0.31)	0.32 (0.04)	2.91 (0.23)	0.9 (0.06)	1.08 (0.13)	0.85 (0.07)	0.35 (0.04)
Syrt-9833-07	18.15 (0.52)	4.89 (0.31)	1.74 (0.07)	5.25 (0.27)	0.74 (0.03)	5.63 (0.24)	1 (0.05)	2.86 (0.15)	0.41 (0.03)	2.49 (0.19)	0.32 (0.03)	3.44 (0.19)	0.94 (0.03)	0.98 (0.09)	0.97 (0.06)	0.32 (0.03)
**Syrt-9833**	**18.29 (0.19)**	**4.86 (0.25)**	**1.65 (0.08)**	**5.25 (0.4)**	**0.79 (0.05)**	**5.43 (0.2)**	**0.98 (0.02)**	**2.92 (0.05)**	**0.39 (0.02)**	**2.42 (0.31)**	**0.34 (0.04)**	**3.29 (0.31)**	**0.88 (0.05)**	**1.06 (0.07)**	**0.91 (0.06)**	**0.32 (0.03)**
Syrt-11221-03	18.23 (0.6)	4.6 (0.27)	1.66 (0.07)	5.27 (0.38)	0.84 (0.05)	5.06 (0.29)	1.03 (0.05)	2.93 (0.16)	0.37 (0.03)	2.86 (0.17)	0.31 (0.02)	3.21 (0.19)	0.98 (0.04)	1.05 (0.08)	0.93 (0.05)	0.37 (0.04)
Syrt-11221-05	18.26 (0.6)	4.82 (0.31)	1.57 (0.08)	5.24 (0.28)	0.76 (0.05)	5.42 (0.23)	0.98 (0.05)	2.8 (0.12)	0.35 (0.03)	2.16 (0.16)	0.33 (0.02)	3.66 (0.25)	0.94 (0.04)	0.95 (0.08)	0.89 (0.06)	0.26 (0.02)
Syrt-11221-07	19.67 (0.6)	4.93 (0.3)	1.79 (0.1)	4.91 (0.27)	0.83 (0.04)	5.2 (0.25)	0.89 (0.05)	2.95 (0.16)	0.45 (0.03)	2.45 (0.16)	0.36 (0.03)	3.44 (0.22)	0.88 (0.04)	1.06 (0.09)	0.93 (0.05)	0.31 (0.03)
**Syrt-11221**	**18.72 (0.75)**	**4.79 (0.16)**	**1.67 (0.1)**	**5.14 (0.17)**	**0.81 (0.04)**	**5.23 (0.17)**	**0.97 (0.07)**	**2.89 (0.07)**	**0.39 (0.05)**	**2.49 (0.34)**	**0.33 (0.02)**	**3.44 (0.21)**	**0.93 (0.03)**	**1.02 (0.06)**	**0.92 (0.03)**	**0.31 (0.06)**
Syrt-9834-01	18.58 (0.56)	4.47 (0.28)	1.74 (0.1)	6.04 (0.32)	0.97 (0.05)	5.74 (0.23)	1.05 (0.06)	3.12 (0.15)	0.43 (0.03)	2.56 (0.17)	0.37 (0.03)	3.25 (0.17)	0.96 (0.04)	0.85 (0.07)	0.98 (0.05)	0.25 (0.03)
Syrt-9834-07	18.8 (0.62)	4.75 (0.32)	1.86 (0.1)	5.18 (0.29)	0.76 (0.05)	5.41 (0.22)	1.05 (0.06)	2.81 (0.16)	0.34 (0.03)	2.61 (0.15)	0.34 (0.03)	3.75 (0.19)	0.94 (0.04)	1.02 (0.09)	1 (0.06)	0.29 (0.03)
Syrt-9834-10	20.37 (0.55)	4.54 (0.28)	1.84 (0.09)	6.07 (0.29)	0.93 (0.04)	5.81 (0.22)	1.07 (0.05)	3.02 (0.13)	0.44 (0.03)	2.62 (0.15)	0.4 (0.03)	3.85 (0.19)	1.02 (0.03)	1.05 (0.08)	0.91 (0.06)	0.27 (0.03)
	1.01	0.14	0.02	0.56	0.12	0.26	0.01	0.13	0.06	0.00	0.04	0.06	0.04	0.02	0.06	0.011984554
Syrt-9831-04	18.6 (0.55)	4.68 (0.23)	1.81 (0.09)	5.61 (0.27)	0.85 (0.04)	5.52 (0.25)	0.97 (0.04)	3.06 (0.13)	0.41 (0.03)	2.57 (0.16)	0.39 (0.03)	3.35 (0.18)	0.87 (0.03)	1.07 (0.09)	1 (0.05)	0.36 (0.03)
Syrt-9831-07	19.06 (0.61)	5.12 (0.32)	1.75 (0.1)	5.18 (0.3)	0.85 (0.05)	5.11 (0.21)	0.98 (0.05)	2.88 (0.15)	0.41 (0.03)	2.38 (0.17)	0.39 (0.03)	3.12 (0.21)	0.93 (0.04)	1.01 (0.07)	1.01 (0.05)	0.31 (0.03)
Syrt-9831-09	16.96 (0.63)	4.91 (0.27)	1.68 (0.08)	4.74 (0.29)	0.74 (0.04)	5 (0.28)	0.94 (0.05)	2.8 (0.12)	0.35 (0.02)	2.3 (0.13)	0.36 (0.02)	3.29 (0.19)	0.86 (0.04)	0.96 (0.07)	0.94 (0.05)	0.3 (0.02)
**Syrt-9831**	**18.2 (1)**	**4.9 (0.21)**	**1.75 (0.06)**	**5.18 (0.38)**	**0.81 (0.06)**	**5.21 (0.25)**	**0.97 (0.02)**	**2.91 (0.12)**	**0.39 (0.03)**	**2.42 (0.13)**	**0.38 (0.02)**	**3.25 (0.11)**	**0.89 (0.03)**	**1.02 (0.05)**	**0.98 (0.03)**	**0.32 (0.04)**
Syrt-9835-02	19.02 (0.67)	5.88 (0.32)	1.84 (0.09)	5.67 (0.27)	0.89 (0.04)	5.61 (0.27)	1.12 (0.06)	3 (0.15)	0.4 (0.03)	2.64 (0.2)	0.33 (0.03)	3.3 (0.21)	0.96 (0.04)	0.97 (0.09)	0.9 (0.05)	0.32 (0.03)
Syrt-9835-05	19.03 (0.59)	5.12 (0.29)	1.82 (0.09)	6.54 (0.34)	0.94 (0.05)	6.08 (0.28)	1.09 (0.06)	3.04 (0.14)	0.45 (0.03)	2.49 (0.18)	0.35 (0.03)	3.81 (0.22)	0.97 (0.03)	1.08 (0.08)	0.93 (0.06)	0.31 (0.03)
Syrt-9835-08	19.69 (0.6)	4.58 (0.22)	1.75 (0.09)	5.82 (0.27)	0.81 (0.04)	5.49 (0.25)	1.02 (0.06)	3.02 (0.12)	0.36 (0.02)	2.8 (0.16)	0.36 (0.03)	3.53 (0.21)	0.91 (0.03)	1.13 (0.09)	0.85 (0.05)	0.32 (0.03)
**Syrt-9835**	**19.25 (0.35)**	**5.19 (0.61)**	**1.8 (0.04)**	**6.01 (0.41)**	**0.88 (0.06)**	**5.73 (0.29)**	**1.08 (0.05)**	**3.02 (0.02)**	**0.4 (0.04)**	**2.64 (0.15)**	**0.35 (0.01)**	**3.55 (0.24)**	**0.95 (0.02)**	**1.06 (0.08)**	**0.9 (0.04)**	**0.32 (0)**
Jol-11222-02	18.26 (0.65)	4.64 (0.4)	1.51 (0.11)	4.93 (0.3)	0.85 (0.08)	5.2 (0.31)	0.89 (0.07)	2.77 (0.19)	0.46 (0.04)	2.45 (0.23)	0.36 (0.03)	3.59 (0.25)	0.9 (0.05)	0.92 (0.11)	0.86 (0.06)	0.25 (0.04)
Jol-11222-07	19.02 (2.04)	3.87 (0.24)	1.73 (0.39)	4.89 (0.55)	0.68 (0.08)	5.12 (0.68)	1.28 (0.1)	2.2 (0.31)	0.19 (0.06)	2.16 (0.33)	0.27 (0.07)	2.8 (0.59)	0.79 (0.07)	1.28 (0.16)	0.93 (0.12)	0.31 (0.09)
Jol-11222-08	19.1 (0.65)	5.14 (0.27)	1.86 (0.09)	5.65 (0.31)	0.77 (0.04)	5.16 (0.23)	1.01 (0.05)	2.88 (0.14)	0.39 (0.03)	2.36 (0.15)	0.35 (0.02)	3.62 (0.18)	0.96 (0.03)	0.92 (0.07)	0.91 (0.06)	0.32 (0.03)
**Jol-11222**	**18.79 (0.42)**	**4.55 (0.59)**	**1.7 (0.16)**	**5.16 (0.38)**	**0.77 (0.08)**	**5.16 (0.04)**	**1.06 (0.19)**	**2.62 (0.33)**	**0.35 (0.12)**	**2.32 (0.14)**	**0.32 (0.05)**	**3.33 (0.43)**	**0.88 (0.06)**	**1.04 (0.21)**	**0.9 (0.03)**	**0.29 (0.04)**
Jol-9836-01	17.96 (0.58)	5.06 (0.28)	1.72 (0.09)	5.55 (0.36)	0.9 (0.04)	5.83 (0.25)	1.07 (0.05)	2.54 (0.14)	0.43 (0.03)	2.55 (0.19)	0.4 (0.03)	3.37 (0.19)	0.92 (0.04)	0.88 (0.09)	1.01 (0.05)	0.32 (0.03)
Jol-9836-02	18.48 (0.9)	5.4 (0.38)	1.77 (0.12)	5.93 (0.39)	0.97 (0.1)	5.65 (0.35)	1.03 (0.08)	3.21 (0.17)	0.39 (0.04)	1.84 (0.18)	0.28 (0.04)	3.42 (0.35)	0.82 (0.04)	0.85 (0.08)	0.93 (0.08)	0.24 (0.04)
Jol-9836-03	18.05 (0.54)	4.63 (0.26)	1.65 (0.08)	5.37 (0.3)	0.89 (0.04)	5.34 (0.23)	1.08 (0.05)	2.69 (0.13)	0.43 (0.03)	2.36 (0.19)	0.34 (0.03)	3.25 (0.15)	0.9 (0.03)	0.84 (0.07)	0.99 (0.06)	0.31 (0.03)
**Jol-9836**	**18.16 (0.25)**	**5.03 (0.36)**	**1.71 (0.05)**	**5.62 (0.25)**	**0.92 (0.04)**	**5.61 (0.23)**	**1.06 (0.02)**	**2.81 (0.31)**	**0.42 (0.02)**	**2.25 (0.36)**	**0.34 (0.06)**	**3.35 (0.08)**	**0.88 (0.04)**	**0.86 (0.02)**	**0.98 (0.04)**	**0.29 (0.04)**
Jol-9837-02	18.81 (0.6)	4.31 (0.26)	1.8 (0.08)	5.43 (0.26)	0.92 (0.05)	5.43 (0.24)	0.94 (0.05)	2.91 (0.14)	0.41 (0.03)	2.32 (0.17)	0.33 (0.03)	3.49 (0.19)	0.88 (0.03)	0.99 (0.07)	0.97 (0.06)	0.28 (0.03)
Jol-9837-05	17.98 (0.45)	5.21 (0.27)	1.65 (0.09)	5.31 (0.28)	0.85 (0.04)	5.65 (0.21)	1.09 (0.05)	2.7 (0.14)	0.38 (0.03)	2.37 (0.17)	0.31 (0.03)	3.65 (0.24)	0.95 (0.04)	0.88 (0.07)	0.79 (0.04)	0.25 (0.03)
Jol-9837-07	18.21 (0.55)	4.81 (0.34)	1.78 (0.09)	4.94 (0.26)	0.87 (0.05)	5.62 (0.24)	0.94 (0.05)	3.08 (0.15)	0.4 (0.03)	2.42 (0.15)	0.41 (0.03)	3.83 (0.19)	0.79 (0.03)	0.95 (0.07)	0.83 (0.04)	0.28 (0.03)
**Jol-9837**	**18.33 (0.39)**	**4.78 (0.42)**	**1.74 (0.07)**	**5.23 (0.23)**	**0.88 (0.03)**	**5.56 (0.11)**	**0.99 (0.08)**	**2.89 (0.17)**	**0.4 (0.02)**	**2.37 (0.05)**	**0.35 (0.05)**	**3.66 (0.16)**	**0.87 (0.05)**	**0.94 (0.05)**	**0.86 (0.09)**	**0.27 (0.02)**
Jol-9843-02	17.86 (0.57)	4.95 (0.25)	1.8 (0.08)	5.07 (0.27)	0.82 (0.04)	5.26 (0.24)	1.09 (0.04)	2.8 (0.14)	0.34 (0.03)	2.26 (0.14)	0.34 (0.02)	2.99 (0.16)	0.83 (0.03)	0.92 (0.08)	0.86 (0.05)	0.25 (0.02)
Jol-9843-05	18.3 (0.59)	4.71 (0.29)	1.75 (0.09)	5.53 (0.34)	0.93 (0.04)	5.26 (0.2)	0.99 (0.04)	2.65 (0.14)	0.36 (0.03)	2.59 (0.18)	0.28 (0.02)	3.05 (0.17)	0.87 (0.03)	0.77 (0.07)	0.81 (0.06)	0.27 (0.03)
Jol-9843-08	16.61 (0.63)	4.42 (0.26)	1.7 (0.09)	5.06 (0.27)	0.84 (0.05)	5.26 (0.21)	0.99 (0.05)	2.39 (0.12)	0.37 (0.03)	2.25 (0.15)	0.29 (0.03)	3.08 (0.18)	0.78 (0.03)	1.13 (0.09)	0.78 (0.05)	0.27 (0.03)
**Jol-9843**	**17.59 (0.8)**	**4.7 (0.25)**	**1.75 (0.04)**	**5.22 (0.24)**	**0.87 (0.05)**	**5.26 (0)**	**1.02 (0.05)**	**2.62 (0.18)**	**0.35 (0.01)**	**2.36 (0.19)**	**0.31 (0.03)**	**3.04 (0.04)**	**0.83 (0.03)**	**0.94 (0.18)**	**0.82 (0.04)**	**0.26 (0.01)**
Jol-9846-03	17.38 (0.61)	4.6 (0.29)	1.73 (0.1)	5.75 (0.3)	0.89 (0.05)	5.45 (0.25)	0.99 (0.04)	2.59 (0.14)	0.43 (0.03)	2.23 (0.15)	0.34 (0.03)	3.67 (0.2)	0.81 (0.03)	0.77 (0.07)	0.79 (0.06)	0.23 (0.02)
Jol-9846-09	17.29 (0.61)	5.27 (0.34)	1.76 (0.09)	5.35 (0.26)	0.82 (0.06)	5.36 (0.25)	1.07 (0.05)	2.69 (0.14)	0.39 (0.03)	2.29 (0.19)	0.34 (0.02)	3.4 (0.18)	0.9 (0.03)	0.84 (0.08)	0.82 (0.05)	0.25 (0.03)
Jol-9846-10	18.08 (0.5)	4.74 (0.34)	1.74 (0.09)	5.7 (0.27)	0.98 (0.05)	5.48 (0.23)	1.05 (0.06)	2.81 (0.16)	0.41 (0.03)	2.4 (0.16)	0.36 (0.03)	3.55 (0.17)	0.73 (0.03)	0.89 (0.07)	0.8 (0.05)	0.25 (0.03)
**Jol-9846**	**17.58 (0.39)**	**4.87 (0.33)**	**1.74 (0.01)**	**5.6 (0.19)**	**0.9 (0.08)**	**5.43 (0.06)**	**1.04 (0.04)**	**2.7 (0.1)**	**0.41 (0.02)**	**2.3 (0.08)**	**0.35 (0.01)**	**3.54 (0.12)**	**0.81 (0.06)**	**0.83 (0.06)**	**0.8 (0.02)**	**0.24 (0.01)**
S-19m-06	27.02 (0.73)	6.93 (0.36)	2.21 (0.1)	7.01 (0.31)	0.95 (0.05)	6.8 (0.27)	1.31 (0.06)	4.07 (0.17)	0.5 (0.03)	3.28 (0.18)	0.43 (0.03)	4.79 (0.25)	1.39 (0.04)	1.61 (0.11)	1.73 (0.07)	0.44 (0.03)
S-19m-09	26.56 (0.74)	6.65 (0.37)	2.19 (0.1)	6.46 (0.3)	1.18 (0.06)	6.71 (0.3)	1.36 (0.06)	3.73 (0.15)	0.51 (0.03)	3.1 (0.16)	0.43 (0.03)	4.91 (0.22)	1.46 (0.05)	1.71 (0.11)	1.43 (0.07)	0.46 (0.04)
S-19m-10	26.58 (0.72)	5.88 (0.31)	2.21 (0.1)	6.61 (0.32)	0.99 (0.05)	6.71 (0.28)	1.45 (0.07)	3.55 (0.14)	0.51 (0.03)	3.27 (0.19)	0.44 (0.03)	4.88 (0.22)	1.5 (0.04)	2.56 (0.16)	1.55 (0.08)	0.5 (0.04)
**S-19m**	**26.72 (0.24)**	**6.49 (0.51)**	**2.2 (0.01)**	**6.69 (0.25)**	**1.04 (0.12)**	**6.74 (0.05)**	**1.37 (0.07)**	**3.79 (0.24)**	**0.51 (0)**	**3.22 (0.1)**	**0.44 (0)**	**4.86 (0.06)**	**1.45 (0.04)**	**1.96 (0.52)**	**1.57 (0.15)**	**0.47 (0.03)**
S-48m-04	23.77 (0.9)	5.36 (0.27)	2.11 (0.1)	6.58 (0.33)	0.9 (0.05)	6 (0.26)	1.17 (0.06)	3.23 (0.15)	0.48 (0.03)	2.81 (0.16)	0.45 (0.03)	4.45 (0.23)	1.32 (0.05)	1.63 (0.1)	1.53 (0.08)	0.43 (0.04)
S-48m-05	25.42 (0.61)	6.04 (0.33)	2.16 (0.09)	7.04 (0.3)	1.02 (0.04)	6.49 (0.24)	1.31 (0.06)	3.52 (0.16)	0.56 (0.04)	3.21 (0.18)	0.39 (0.03)	4.39 (0.21)	1.49 (0.05)	1.7 (0.09)	1.58 (0.08)	0.45 (0.03)
S-48m-11	28.94 (0.78)	6.76 (0.35)	2.24 (0.11)	7.84 (0.4)	0.98 (0.04)	6.81 (0.29)	1.34 (0.06)	3.98 (0.16)	0.55 (0.03)	3.42 (0.19)	0.44 (0.03)	4.76 (0.23)	1.52 (0.05)	1.7 (0.09)	1.63 (0.06)	0.55 (0.04)
**S-48m**	**26.04 (2.4)**	**6.06 (0.65)**	**2.17 (0.06)**	**7.15 (0.56)**	**0.97 (0.06)**	**6.43 (0.38)**	**1.27 (0.08)**	**3.57 (0.34)**	**0.53 (0.04)**	**3.15 (0.3)**	**0.43 (0.03)**	**4.54 (0.19)**	**1.44 (0.07)**	**1.68 (0.04)**	**1.58 (0.05)**	**0.48 (0.06)**

S-140-141m-01	46.99 (0.18)	2.59 (0.03)	16.72 (0.11)	11.76 (0.3)	0.18 (0.03)	6.07 (0.1)	9.75 (0.04)	3.84 (0.05)	0.61 (0.01)	0.01 (0.01)	0.25 (0.01)	0.12 (0.01)	98.89	3
S-140-141m-02	47.04 (0.11)	2.53 (0.1)	16.69 (0.11)	11.99 (0.18)	0.18 (0.03)	6.09 (0.11)	9.74 (0.03)	3.8 (0.05)	0.63 (0.03)	0.04 (0.01)	0.31 (0.02)	0.1 (0.02)	99.12	3
S-140-141m-03	46.93 (0.03)	2.62 (0.01)	16.59 (0.06)	12.13 (0.26)	0.2 (0.03)	6.12 (0.1)	9.78 (0.06)	3.58 (0.08)	0.6 (0.04)	0.01 (0.02)	0.26 (0.02)	0.11 (0.01)	98.92	3
S-140-141m-04	46.96 (0.12)	2.54 (0.06)	16.64 (0.13)	11.67 (0.55)	0.2 (0.01)	6.02 (0.16)	9.76 (0.01)	3.88 (0.13)	0.63 (0.01)	0.02 (0.02)	0.28 (0.03)	0.11 (0.02)	98.71	3
S-140-141m-05	47.11 (0.12)	2.63 (0.03)	16.73 (0.07)	12.3 (0.18)	0.18 (0.03)	6.15 (0.12)	9.73 (0.03)	3.82 (0.14)	0.62 (0.04)	0.01 (0.01)	0.24 (0.01)	0.1 (0)	99.61	3
S-140-141m-06	47.03 (0.11)	2.59 (0.07)	16.49 (0.1)	11.7 (0.38)	0.18 (0.01)	6.12 (0.1)	9.8 (0.07)	3.75 (0.09)	0.63 (0.01)	0.02 (0)	0.28 (0.06)	0.12 (0.01)	98.69	3
S-140-141m-07	47.08 (0.24)	2.58 (0.06)	16.67 (0)	12.07 (0.22)	0.23 (0.01)	6.15 (0.09)	9.76 (0.02)	3.76 (0.08)	0.62 (0.03)	0 (0)	0.28 (0.02)	0.1 (0.02)	99.32	3
S-140-141m-08	46.96 (0.13)	2.62 (0.02)	16.53 (0.14)	11.43 (0.53)	0.21 (0.01)	6.08 (0.05)	9.7 (0)	3.71 (0.06)	0.61 (0.03)	0 (0)	0.26 (0.04)	0.1 (0.03)	98.22	3
S-140-141m-09	46.93 (0.15)	2.5 (0.02)	16.7 (0.2)	11.6 (0.35)	0.2 (0.01)	6.01 (0.04)	9.77 (0.12)	3.89 (0.04)	0.62 (0.01)	0.01 (0.01)	0.26 (0.02)	0.14 (0.02)	98.63	3
S-140-141m-10	46.96 (0.03)	2.52 (0.01)	16.56 (0.03)	11.63 (0.38)	0.2 (0.03)	6.03 (0.04)	9.8 (0.02)	3.79 (0.12)	0.63 (0)	0.04 (0.03)	0.24 (0.02)	0.12 (0.02)	98.50	3
**S-140-141m**	**47 (0.06)**	**2.57 (0.05)**	**16.63 (0.09)**	**11.83 (0.28)**	**0.19 (0.02)**	**6.08 (0.05)**	**9.76 (0.03)**	**3.78 (0.09)**	**0.62 (0.01)**	**0.02 (0.01)**	**0.27 (0.02)**	**0.11 (0.01)**	**98.86**	**30**
S-150-151m-03	46.73 (0.08)	2.68 (0.05)	16.45 (0.09)	12.39 (0.29)	0.15 (0.01)	6.11 (0.07)	9.72 (0.04)	3.83 (0.04)	0.64 (0.04)	0.03 (0.02)	0.23 (0.01)	0.09 (0.02)	99.06	3
S-150-151m-04	46.9 (0.07)	2.66 (0.05)	16.52 (0.12)	12.1 (0.03)	0.19 (0.03)	6.15 (0.05)	9.69 (0.08)	3.84 (0.05)	0.61 (0.03)	0.02 (0.03)	0.24 (0)	0.1 (0.02)	99.02	3
S-150-151m-05	46.78 (0.06)	2.69 (0.04)	16.43 (0.02)	12.37 (0.05)	0.18 (0.03)	6.15 (0.07)	9.68 (0.01)	3.81 (0.07)	0.64 (0.03)	0.02 (0.03)	0.22 (0.04)	0.12 (0.01)	99.10	3
S-150-151m-06	46.62 (0.24)	2.74 (0.03)	16.45 (0.08)	12.67 (0.17)	0.2 (0.01)	5.91 (0.05)	9.49 (0.08)	3.94 (0.07)	0.67 (0.03)	0 (0)	0.27 (0.01)	0.11 (0.01)	99.06	3
S-150-151m-07	46.76 (0.17)	2.71 (0.02)	16.63 (0.05)	12.55 (0.12)	0.2 (0.05)	6.18 (0.05)	9.59 (0.06)	3.85 (0.03)	0.64 (0.01)	0.01 (0.01)	0.23 (0.02)	0.12 (0.03)	99.47	3
S-150-151m-08	46.71 (0.11)	2.82 (0.05)	16.39 (0.28)	12.81 (0.18)	0.19 (0.02)	5.83 (0.04)	9.44 (0.05)	3.89 (0.04)	0.7 (0.01)	0.02 (0.02)	0.26 (0.06)	0.11 (0.02)	99.15	3
S-150-151m-09	47.27 (0.24)	2.09 (0.04)	16.52 (0.08)	11.37 (0.15)	0.18 (0.01)	6.62 (0.01)	10.48 (0.16)	3.18 (0.09)	0.57 (0.01)	0.03 (0.01)	0.17 (0.02)	0.11 (0.02)	98.59	3
S-150-151m-10	46.91 (0.08)	2.58 (0.02)	16.45 (0.09)	12.57 (0.31)	0.19 (0.02)	6.02 (0.07)	9.73 (0.05)	3.54 (0.11)	0.6 (0.01)	0.02 (0.02)	0.17 (0.02)	0.09 (0)	98.88	3
S-150-151m-11	46.77 (0.15)	2.57 (0.03)	16.52 (0.05)	11.62 (0.29)	0.23 (0.04)	6.04 (0.09)	9.81 (0.05)	3.79 (0.16)	0.65 (0.01)	0.02 (0.02)	0.26 (0.05)	0.14 (0.01)	98.43	3
S-150-151m-12	46.8 (0.16)	2.58 (0.03)	16.67 (0.17)	12 (0.05)	0.18 (0.01)	6.13 (0.04)	9.73 (0.05)	3.77 (0.06)	0.66 (0.01)	0.01 (0.01)	0.27 (0.03)	0.11 (0.01)	98.91	3
**S-150-151m**	**46.83 (0.18)**	**2.61 (0.2)**	**16.5 (0.09)**	**12.25 (0.47)**	**0.19 (0.02)**	**6.11 (0.21)**	**9.74 (0.29)**	**3.74 (0.22)**	**0.64 (0.04)**	**0.02 (0.01)**	**0.23 (0.04)**	**0.11 (0.01)**	**98.97**	**30**
S-158-159m-01	46.74 (0.17)	2.75 (0.01)	16.62 (0.1)	12.36 (0.03)	0.2 (0.01)	5.94 (0.06)	9.55 (0.11)	3.86 (0.04)	0.66 (0.03)	0.01 (0.01)	0.24 (0.03)	0.08 (0.02)	99.06	3
S-158-159m-04	46.6 (0.18)	2.91 (0.05)	16.22 (0.08)	13.22 (0.38)	0.2 (0.04)	5.91 (0.16)	9.2 (0.08)	4.04 (0.18)	0.72 (0.02)	0 (0)	0.25 (0)	0.11 (0.02)	99.37	3
S-158-159m-05	46.76 (0.17)	2.94 (0.05)	16.12 (0.04)	13.26 (0.1)	0.2 (0.03)	5.76 (0.12)	9.13 (0.06)	4.15 (0.1)	0.74 (0.01)	0.03 (0.01)	0.25 (0.01)	0.12 (0.01)	99.45	3
S-158-159m-06	46.61 (0.01)	2.86 (0.02)	16.48 (0.12)	12.72 (0.04)	0.17 (0.01)	5.66 (0.08)	9.25 (0.04)	4.06 (0.09)	0.71 (0.01)	0.01 (0.01)	0.28 (0.01)	0.11 (0.01)	98.92	3
S-158-159m-07	46.95 (0.26)	2.74 (0.05)	16.48 (0.1)	12.35 (0.24)	0.21 (0.02)	5.95 (0.06)	9.5 (0.11)	3.96 (0.14)	0.73 (0.04)	0.01 (0.01)	0.26 (0.02)	0.1 (0.02)	99.25	3
S-158-159m-08	46.58 (0.13)	2.94 (0.06)	16.28 (0.09)	12.8 (0.32)	0.19 (0.03)	5.82 (0.1)	9.27 (0.07)	3.99 (0.08)	0.73 (0.05)	0.01 (0.02)	0.26 (0.01)	0.11 (0.01)	98.99	3
S-158-159m-09	46.73 (0.07)	2.91 (0.03)	16.47 (0.14)	12.74 (0.36)	0.19 (0.03)	5.72 (0.03)	9.07 (0.08)	4.39 (0.14)	0.75 (0.02)	0.01 (0.02)	0.24 (0.03)	0.11 (0.01)	99.33	3
S-158-159m-10	46.49 (0.06)	2.99 (0.1)	15.99 (0.14)	13.36 (0.16)	0.22 (0.02)	5.59 (0.02)	9.36 (0.02)	3.96 (0.08)	0.76 (0.03)	0.02 (0.02)	0.29 (0.03)	0.09 (0.01)	99.10	3
S-158-159m-11	46.74 (0.1)	2.73 (0.07)	16.47 (0.1)	12.45 (0.48)	0.19 (0.02)	5.89 (0.07)	9.75 (0.08)	3.94 (0.06)	0.65 (0.02)	0 (0)	0.26 (0.02)	0.1 (0.02)	99.16	3
S-158-159m-12	46.76 (0.08)	2.68 (0.04)	16.43 (0.24)	12.44 (0.17)	0.21 (0.03)	6.14 (0.02)	9.57 (0.22)	3.92 (0.13)	0.65 (0.02)	0.03 (0.01)	0.25 (0)	0.13 (0.02)	99.20	3
**S-158-159m**	**46.69 (0.13)**	**2.85 (0.11)**	**16.36 (0.2)**	**12.77 (0.39)**	**0.2 (0.01)**	**5.84 (0.16)**	**9.37 (0.22)**	**4.03 (0.15)**	**0.71 (0.04)**	**0.01 (0.01)**	**0.26 (0.02)**	**0.11 (0.02)**	**99.18**	**30**
S-169-170m-01	46.69 (0.21)	2.89 (0.03)	16.5 (0.06)	12.79 (0.1)	0.2 (0.01)	5.58 (0.02)	9.2 (0.02)	4.07 (0.11)	0.7 (0.03)	0.02 (0.02)	0.27 (0.01)	0.12 (0.02)	99.02	3
S-169-170m-02	46.61 (0.19)	2.95 (0.03)	16.3 (0.04)	13.13 (0.52)	0.2 (0.01)	5.64 (0.07)	9.14 (0.13)	4.02 (0.05)	0.71 (0.02)	0.02 (0.01)	0.26 (0.03)	0.08 (0.01)	99.05	3
S-169-170m-03	46.77 (0.11)	2.89 (0.03)	16.46 (0.07)	12.85 (0.34)	0.19 (0.02)	5.65 (0.05)	9.24 (0.08)	4.1 (0.02)	0.71 (0.01)	0.01 (0.02)	0.25 (0.02)	0.1 (0.01)	99.23	3
S-169-170m-05	46.56 (0.17)	2.94 (0.05)	16.35 (0.15)	13.3 (0.13)	0.21 (0.03)	5.69 (0.1)	9.18 (0.09)	4.04 (0.06)	0.73 (0.02)	0 (0)	0.27 (0.04)	0.11 (0.01)	99.37	3
S-169-170m-06	46.47 (0.13)	2.95 (0.04)	16.26 (0.16)	12.82 (0.27)	0.23 (0.01)	5.47 (0.01)	9.19 (0.07)	4.03 (0.04)	0.73 (0.03)	0.02 (0.02)	0.23 (0.04)	0.09 (0.03)	98.51	3
S-169-170m-07	46.59 (0.26)	2.93 (0.05)	16.43 (0.13)	12.85 (0.17)	0.2 (0.02)	5.39 (0.05)	9.21 (0.07)	4 (0.18)	0.74 (0.03)	0 (0.01)	0.27 (0.04)	0.1 (0.02)	98.71	3
S-169-170m-08	46.62 (0)	2.99 (0.02)	16.42 (0.11)	13.01 (0.32)	0.19 (0.02)	5.38 (0.05)	9.28 (0.02)	4.17 (0.17)	0.74 (0.02)	0.01 (0.01)	0.26 (0.02)	0.1 (0.01)	99.17	2
S-169-170m-09	46.55 (0.08)	3.01 (0.03)	16.24 (0.04)	12.98 (0.44)	0.21 (0.04)	5.57 (0.04)	9.11 (0.02)	4.09 (0.13)	0.74 (0.02)	0.02 (0.01)	0.27 (0.04)	0.12 (0.02)	98.90	3
S-169-170m-10	46.64 (0.12)	2.94 (0.01)	16.34 (0.08)	12.7 (0.3)	0.2 (0.02)	5.64 (0.05)	9.17 (0.1)	3.98 (0.09)	0.69 (0.01)	0.01 (0.01)	0.27 (0.04)	0.11 (0.01)	98.69	3
S-169-170m-13	46.6 (0.21)	2.91 (0.03)	16.41 (0.17)	12.99 (0.12)	0.19 (0.04)	5.63 (0.07)	9.23 (0.04)	4.12 (0.06)	0.69 (0.01)	0.02 (0.02)	0.25 (0.06)	0.12 (0.03)	99.15	3
**S-169-170m**	**46.61 (0.08)**	**2.94 (0.04)**	**16.37 (0.09)**	**12.94 (0.18)**	**0.2 (0.01)**	**5.56 (0.11)**	**9.19 (0.05)**	**4.06 (0.06)**	**0.72 (0.02)**	**0.01 (0.01)**	**0.26 (0.01)**	**0.1 (0.01)**	**98.98**	**29**
S-170m-01	45.76 (0.09)	2.83 (0.01)	16.08 (0.02)	12.7 (0.07)	0.19 (0.04)	5.47 (0.03)	9.07 (0.1)	4 (0.02)	0.7 (0.01)	0 (0)	0.3 (0)	0.12 (0)	97.22	2
S-170m-02	46.35 (0.3)	2.7 (0.03)	16.29 (0.09)	12.32 (0.26)	0.2 (0.02)	6.05 (0.12)	9.61 (0.09)	3.77 (0.05)	0.66 (0.02)	0.03 (0.02)	0.24 (0.03)	0.1 (0.01)	98.32	3
S-170m-06	46.34 (0.05)	2.67 (0.02)	16.4 (0.06)	12.39 (0.29)	0.21 (0.02)	6 (0.02)	9.57 (0.09)	3.75 (0.05)	0.64 (0.02)	0.02 (0.02)	0.22 (0.02)	0.11 (0.02)	98.32	3
S-170m-07	46.47 (0.23)	2.9 (0.03)	16.27 (0.05)	13.11 (0.18)	0.19 (0.02)	5.46 (0.03)	9.1 (0.08)	3.9 (0.13)	0.74 (0.04)	0 (0)	0.27 (0.05)	0.12 (0.01)	98.53	2
S-170m-08	46.26 (0.15)	2.87 (0.08)	16.2 (0.14)	13.05 (0.25)	0.19 (0.02)	5.69 (0.02)	9.31 (0.02)	3.88 (0.09)	0.69 (0.02)	0.01 (0.01)	0.26 (0.02)	0.1 (0.01)	98.49	3
S-170m-09	46.23 (0.28)	3.01 (0.04)	15.97 (0.07)	13.15 (0.08)	0.2 (0.02)	5.46 (0.07)	9.18 (0.05)	3.85 (0.22)	0.72 (0.01)	0.02 (0.02)	0.27 (0.03)	0.12 (0.03)	98.18	3
S-170m-10	45.99 (0.44)	2.85 (0.01)	16.22 (0.17)	12.85 (0.26)	0.23 (0.03)	5.5 (0.1)	9.24 (0.09)	4.08 (0.06)	0.72 (0)	0.01 (0)	0.26 (0.04)	0.1 (0.02)	98.03	2
**S-170m**	**46.2 (0.24)**	**2.83 (0.12)**	**16.2 (0.14)**	**12.79 (0.34)**	**0.2 (0.01)**	**5.66 (0.26)**	**9.3 (0.22)**	**3.89 (0.12)**	**0.7 (0.03)**	**0.01 (0.01)**	**0.26 (0.02)**	**0.11 (0.01)**	**98.16**	**18**
S-176-177m-01	46.15 (0.14)	2.92 (0.02)	16.33 (0.04)	13.09 (0.12)	0.18 (0.03)	5.48 (0.01)	9.24 (0.01)	4.02 (0.15)	0.71 (0.01)	0.02 (0.01)	0.24 (0.02)	0.09 (0.01)	98.47	3
S-176-177m-03	46.22 (0.29)	3.02 (0.05)	16.09 (0.13)	13.08 (0.4)	0.16 (0.08)	5.38 (0.02)	9.18 (0.05)	3.91 (0.09)	0.71 (0.03)	0 (0.01)	0.26 (0.03)	0.07 (0.02)	98.08	3
S-176-177m-04	46.08 (0.39)	3.09 (0.03)	15.9 (0.06)	13.46 (0.25)	0.23 (0.02)	5.46 (0.04)	9.12 (0.03)	4.03 (0.09)	0.71 (0.01)	0.02 (0.02)	0.25 (0.08)	0.09 (0.02)	98.44	3
S-176-177m-05	46.21 (0.1)	2.97 (0.02)	16.24 (0.1)	12.87 (0.31)	0.19 (0.04)	5.51 (0.08)	9.16 (0.17)	4.01 (0.13)	0.69 (0.03)	0.02 (0.03)	0.32 (0.02)	0.1 (0.01)	98.28	3
S-176-177m-06	46.16 (0.16)	2.91 (0.03)	16.36 (0.13)	12.65 (0.54)	0.2 (0.01)	5.64 (0.07)	9.28 (0.02)	3.93 (0.03)	0.7 (0.03)	0.01 (0.01)	0.27 (0.02)	0.09 (0)	98.20	3
S-176-177m-07	46.55 (0.24)	2.93 (0.05)	16.53 (0.07)	13.04 (0.09)	0.18 (0.01)	5.57 (0.05)	9.27 (0.04)	3.85 (0.11)	0.68 (0.02)	0.01 (0.02)	0.26 (0.02)	0.1 (0.03)	98.98	3
S-176-177m-08	46.64 (0.06)	2.95 (0.05)	16.51 (0.07)	12.9 (0.22)	0.19 (0.03)	5.62 (0.05)	9.14 (0.01)	4 (0.1)	0.7 (0.02)	0.01 (0.01)	0.27 (0.03)	0.09 (0.01)	99.02	3
S-176-177m-09	46.29 (0.32)	2.85 (0.07)	16.5 (0.09)	12.76 (0.23)	0.2 (0.03)	5.53 (0.03)	9.26 (0.14)	4.04 (0.1)	0.7 (0.02)	0.02 (0.02)	0.25 (0.03)	0.1 (0.01)	98.50	3
S-176-177m-10	46.11 (0.04)	2.92 (0.06)	16.26 (0.11)	12.75 (0.3)	0.21 (0.02)	5.66 (0.08)	9.23 (0.07)	4.01 (0.04)	0.71 (0.05)	0.01 (0)	0.27 (0.04)	0.12 (0.03)	98.26	3
**S-176-177m**	**46.27 (0.2)**	**2.95 (0.07)**	**16.3 (0.21)**	**12.96 (0.25)**	**0.19 (0.02)**	**5.54 (0.09)**	**9.21 (0.06)**	**3.98 (0.07)**	**0.7 (0.01)**	**0.01 (0.01)**	**0.26 (0.02)**	**0.09 (0.01)**	**98.47**	**27**

S-140-141m-02	28.2 (0.79)	268.88 (4.32)	42.18 (0.86)	70.19 (2.27)	57.67 (1.7)	88.13 (3.25)	23.43 (0.59)	2.53 (0.38)	14.01 (0.38)	368.62 (5.1)	34.96 (0.57)	195.37 (2.73)	24.09 (0.41)	173.49 (3.58)	18.2 (0.35)	44.65 (0.7)	5.91 (0.12)	26.28 (0.7)	6.77 (0.32)	2.11 (0.09)	6.93 (0.29)	1.09 (0.05)	7 (0.25)	1.19 (0.05)	3.72 (0.18)	0.55 (0.03)	3.31 (0.16)	0.52 (0.04)	4.58 (0.21)	1.39 (0.04)	1.65 (0.11)	1.47 (0.06)	0.47 (0.03)
S-140-141m-03	30.73 (1.24)	284.17 (6.21)	42.65 (1.49)	58.47 (2.6)	53.6 (2.78)	89.85 (4.69)	21.57 (0.81)	2.62 (0.78)	14.64 (0.53)	361.72 (7.11)	36.88 (0.78)	197.59 (4.92)	23.69 (0.6)	157.95 (3.79)	18.72 (0.62)	42.85 (1)	5.34 (0.23)	25.71 (1.19)	6.82 (0.51)	1.75 (0.15)	7.3 (0.47)	1.13 (0.06)	6.86 (0.39)	1.08 (0.06)	4.05 (0.21)	0.45 (0.04)	3.57 (0.36)	0.43 (0.05)	4.67 (0.34)	1.45 (0.08)	1.69 (0.12)	1.42 (0.1)	0.46 (0.06)
S-140-141m-05	29.03 (0.76)	270.18 (4.2)	43.22 (0.97)	66.88 (2.06)	62.03 (1.82)	92.2 (3.13)	23.87 (0.72)	1.79 (0.46)	14.02 (0.41)	368.92 (5.16)	35.66 (0.59)	197.05 (2.9)	23.23 (0.38)	165.11 (3.1)	18.43 (0.39)	43.31 (0.69)	5.82 (0.14)	25.92 (0.68)	6 (0.29)	2.09 (0.1)	6.89 (0.33)	1.17 (0.06)	6.31 (0.23)	1.25 (0.06)	3.39 (0.14)	0.5 (0.03)	3.5 (0.21)	0.46 (0.03)	4.24 (0.25)	1.54 (0.04)	1.51 (0.1)	1.43 (0.07)	0.48 (0.04)
**S-140-141m**	**29.32 (1.17)**	**274.41 (8.25)**	**42.68 (0.52)**	**65.18 (5.81)**	**57.77 (3.77)**	**90.06 (2.56)**	**22.96 (1.19)**		**14.22 (0.37)**	**366.42 (3.78)**	**35.83 (0.87)**	**196.67 (1.05)**	**23.67 (0.31)**	**165.52 (6.75)**	**18.45 (0.24)**	**43.6 (0.83)**	**5.69 (0.27)**	**25.97 (0.26)**	**6.53 (0.43)**	**1.98 (0.18)**	**7.04 (0.2)**	**1.13 (0.04)**	**6.72 (0.34)**	**1.17 (0.09)**	**3.72 (0.29)**	**0.5 (0.04)**	**3.46 (0.13)**	**0.47 (0.04)**	**4.5 (0.21)**	**1.46 (0.05)**	**1.62 (0.09)**	**1.44 (0.03)**	**0.47 (0.01)**
S-150-151m-07	28.51 (0.82)	281.32 (4.29)	43.97 (0.96)	61.19 (1.94)	60.25 (1.65)	93.38 (3.18)	22.58 (0.64)	1.68 (0.59)	14.47 (0.38)	357.78 (4.7)	35.39 (0.59)	196.07 (2.88)	23.45 (0.36)	167.26 (3.16)	18.98 (0.35)	43.82 (0.76)	6.04 (0.17)	28.12 (0.79)	7.06 (0.42)	2.33 (0.09)	7.17 (0.33)	1.17 (0.06)	7.01 (0.26)	1.39 (0.07)	4.02 (0.19)	0.46 (0.03)	3.23 (0.19)	0.49 (0.03)	4.35 (0.2)	1.61 (0.05)	1.65 (0.1)	1.51 (0.06)	0.51 (0.04)
S-150-151m-11	27.57 (0.69)	269.44 (4.08)	42.3 (1)	68.32 (2.01)	61.55 (1.6)	125.28 (38.83)	23.45 (0.63)	1.42 (0.49)	14.27 (0.44)	375.91 (5.09)	35.97 (0.63)	201.17 (3.2)	23.46 (0.38)	173.46 (3.52)	18.28 (0.35)	44.27 (0.75)	5.68 (0.13)	25.49 (0.64)	6.29 (0.32)	2.17 (0.1)	6.51 (0.29)	1.11 (0.05)	6.64 (0.22)	1.35 (0.06)	3.55 (0.16)	0.58 (0.03)	3.05 (0.16)	0.54 (0.04)	4.76 (0.25)	1.42 (0.04)	1.57 (0.1)	1.56 (0.07)	0.48 (0.04)
S-150-151m-12	27.23 (0.8)	268.71 (4.31)	41.77 (0.8)	66.95 (2.07)	58.18 (1.69)	92.14 (3.34)	23.14 (0.62)		14.06 (0.41)	373.28 (4.94)	34.54 (0.56)	201.22 (2.89)	23.4 (0.39)	169.56 (3.22)	18.02 (0.38)	43.6 (0.73)	5.77 (0.16)	26.6 (0.8)	6.1 (0.31)	2.24 (0.11)	6.92 (0.33)	1.24 (0.06)	6.93 (0.25)	1.25 (0.06)	3.55 (0.14)	0.43 (0.03)	3.15 (0.19)	0.46 (0.03)	5.14 (0.22)	1.45 (0.04)	1.66 (0.11)	1.61 (0.07)	0.44 (0.04)
**S-150-151m**	**27.77 (0.6)**	**273.16 (6.89)**	**42.68 (1.14)**	**65.49 (3.64)**	**59.99 (1.52)**	**103.6 (23.55)**	**23.06 (0.43)**		**14.27 (0.21)**	**368.99 (9.1)**	**35.3 (0.64)**	**199.49 (2.68)**	**23.43 (0.02)**	**170.09 (2.72)**	**18.43 (0.46)**	**43.9 (0.31)**	**5.83 (0.16)**	**26.74 (1.2)**	**6.49 (0.47)**	**2.25 (0.07)**	**6.87 (0.29)**	**1.17 (0.06)**	**6.86 (0.18)**	**1.33 (0.07)**	**3.7 (0.24)**	**0.49 (0.07)**	**3.14 (0.08)**	**0.5 (0.04)**	**4.75 (0.37)**	**1.5 (0.07)**	**1.63 (0.05)**	**1.56 (0.05)**	**0.48 (0.04)**
S-158-159m-04	28.39 (0.78)	285.35 (4.36)	41.2 (0.93)	55.3 (1.91)	64.73 (2.04)	96.95 (3.35)	24.51 (0.62)	1.65 (0.59)	15.34 (0.43)	360.84 (4.91)	38.82 (0.65)	216.77 (3.12)	26.75 (0.44)	180.55 (3.22)	20.06 (0.37)	47.8 (0.74)	6.45 (0.16)	29.93 (0.73)	6.59 (0.33)	2.43 (0.11)	7.37 (0.36)	1.23 (0.05)	6.83 (0.27)	1.5 (0.07)	4.1 (0.17)	0.59 (0.04)	3.35 (0.15)	0.55 (0.04)	5.17 (0.28)	1.59 (0.05)	1.88 (0.1)	1.67 (0.08)	0.51 (0.03)
S-158-159m-07	28.79 (0.93)	280.76 (4.4)	41.78 (0.93)	60.09 (1.89)	60.19 (1.81)	100.98 (6.74)	22.97 (0.66)	1.83 (0.5)	15.37 (0.44)	367.71 (5.17)	36.45 (0.63)	208.31 (3.31)	24.82 (0.41)	174.32 (3.47)	19.46 (0.36)	46.7 (0.7)	6.17 (0.15)	28.43 (0.85)	6.47 (0.37)	2.38 (0.11)	6.98 (0.3)	1.13 (0.05)	7.29 (0.27)	1.38 (0.06)	3.73 (0.18)	0.55 (0.04)	3.06 (0.19)	0.5 (0.04)	5.22 (0.22)	1.61 (0.05)	1.6 (0.09)	1.7 (0.07)	0.44 (0.03)
S-158-159m-12	26.94 (0.79)	273.14 (4.78)	43.83 (1.07)	62.83 (2.36)	64.95 (1.84)	102.06 (3.75)	26.02 (0.78)		14.19 (0.47)	359.74 (5.08)	35.44 (0.72)	188.66 (3.4)	23.55 (0.42)	163.56 (3.1)	18.37 (0.43)	43.49 (0.83)	5.69 (0.16)	25.27 (0.81)	5.25 (0.31)	1.87 (0.09)	6.22 (0.32)	1.09 (0.07)	6.24 (0.26)	1.37 (0.06)	3.11 (0.2)	0.51 (0.04)	2.94 (0.23)	0.53 (0.04)	4.11 (0.24)	1.39 (0.04)	1.85 (0.12)	1.36 (0.08)	0.46 (0.05)
**S-158-159m**	**28.04 (0.88)**	**279.75 (6)**	**42.27 (1.37)**	**59.41 (3.66)**	**63.29 (2.4)**	**99.99 (3.38)**	**24.5 (1.49)**		**14.97 (0.69)**	**362.76 (4.01)**	**36.9 (1.55)**	**204.58 (13.09)**	**25.04 (1.18)**	**172.81 (7.46)**	**19.3 (0.8)**	**46 (2)**	**6.1 (0.34)**	**27.88 (2.16)**	**6.1 (0.69)**	**2.22 (0.28)**	**6.85 (0.52)**	**1.15 (0.07)**	**6.79 (0.49)**	**1.42 (0.07)**	**3.65 (0.45)**	**0.55 (0.04)**	**3.12 (0.2)**	**0.53 (0.02)**	**4.84 (0.58)**	**1.53 (0.08)**	**1.78 (0.16)**	**1.58 (0.18)**	**0.47 (0.04)**
S-169-170m-03	26.45 (0.85)	262.99 (4.21)	55.3 (2.26)	141.24 (11.4)	53.78 (1.87)	98.3 (3.8)	21.93 (0.62)	1.37 (0.49)	14.54 (0.44)	344.53 (5.45)	35.13 (0.68)	198.44 (3.22)	23.39 (0.4)	162.37 (3.44)	18.12 (0.42)	44.24 (0.7)	5.81 (0.14)	26.15 (0.68)	6.23 (0.33)	2.17 (0.11)	6.66 (0.35)	1.13 (0.05)	6.73 (0.27)	1.29 (0.06)	3.88 (0.16)	0.45 (0.03)	3.14 (0.2)	0.5 (0.03)	4.57 (0.29)	1.5 (0.05)	1.72 (0.1)	1.65 (0.06)	0.5 (0.03)
S-169-170m-06	28.92 (0.8)	290.51 (4.29)	42.52 (0.96)	51.86 (1.99)	59.04 (1.83)	101.46 (3.11)	25.05 (0.73)	1.83 (0.54)	16.11 (0.48)	367.71 (5.07)	39.94 (0.66)	230.05 (3.34)	27.27 (0.43)	183.26 (3.42)	21.37 (0.4)	50.9 (0.81)	6.7 (0.14)	30.61 (0.78)	6.77 (0.39)	2.43 (0.09)	7.7 (0.35)	1.25 (0.05)	7.58 (0.28)	1.44 (0.06)	4.12 (0.16)	0.55 (0.04)	3.79 (0.21)	0.54 (0.03)	5.21 (0.24)	1.84 (0.06)	2.03 (0.09)	1.77 (0.07)	0.54 (0.04)
S-169-170m-07	26.64 (0.77)	283.08 (4.51)	43.97 (0.96)	55.73 (1.93)	59.73 (1.82)	135.52 (37.59)	24.94 (0.67)	1.27 (0.54)	14.7 (0.45)	361.13 (5.58)	39.34 (0.66)	217.66 (3.47)	25.94 (0.41)	177.96 (3.39)	19.42 (0.39)	47.75 (0.8)	6.34 (0.17)	28 (0.76)	6.87 (0.36)	2.23 (0.12)	7.48 (0.38)	1.15 (0.05)	7.65 (0.32)	1.55 (0.07)	3.83 (0.2)	0.65 (0.04)	3.05 (0.2)	0.58 (0.04)	4.96 (0.24)	1.66 (0.05)	2.01 (0.12)	1.72 (0.07)	0.53 (0.04)
**S-169-170m**	**27.34 (1.25)**	**278.86 (13.86)**	**47.27 (6.93)**	**82.94 (48.52)**	**57.52 (2.92)**	**111.76 (25.88)**	**23.97 (1.73)**		**15.11 (0.89)**	**357.79 (11.09)**	**38.14 (2.35)**	**215.38 (14.46)**	**25.53 (1.44)**	**174.53 (9.42)**	**19.64 (1.52)**	**47.63 (2.98)**	**6.28 (0.4)**	**28.25 (2.04)**	**6.62 (0.32)**	**2.28 (0.12)**	**7.28 (0.48)**	**1.18 (0.06)**	**7.32 (0.48)**	**1.43 (0.13)**	**3.94 (0.14)**	**0.55 (0.09)**	**3.33 (0.39)**	**0.54 (0.04)**	**4.91 (0.3)**	**1.67 (0.12)**	**1.92 (0.18)**	**1.71 (0.06)**	**0.52 (0.02)**
S-170m-02	29.73 (0.81)	277.85 (4.69)	43.82 (1.07)	62.82 (2.55)	59.14 (1.84)	80.36 (3.11)	23.1 (0.65)	1.44 (0.46)	14.2 (0.39)	355.58 (5)	35.24 (0.62)	197.69 (2.9)	23.76 (0.34)	168.24 (3.62)	17.5 (0.36)	44.23 (0.73)	6.12 (0.17)	26.79 (0.8)	6.81 (0.38)	2.14 (0.1)	7.23 (0.36)	1.05 (0.06)	6.61 (0.26)	1.32 (0.06)	3.75 (0.16)	0.57 (0.04)	3.13 (0.21)	0.49 (0.04)	4.51 (0.23)	1.4 (0.05)	1.62 (0.1)	1.56 (0.07)	0.49 (0.03)
S-170m-06	27.08 (0.71)	272.29 (4.6)	43.76 (0.83)	62.57 (2.03)	62.04 (1.9)	99.22 (10.94)	24.31 (0.62)		14.13 (0.35)	354.85 (5.62)	34.07 (0.62)	192.3 (3.21)	23.89 (0.38)	163.1 (3.44)	18.02 (0.42)	43.56 (0.77)	5.86 (0.17)	26.97 (0.78)	6.62 (0.37)	2.07 (0.11)	6.93 (0.28)	1.07 (0.05)	6.57 (0.23)	1.29 (0.06)	3.46 (0.14)	0.56 (0.03)	3.23 (0.16)	0.54 (0.03)	5.26 (0.23)	1.42 (0.05)	1.8 (0.1)	1.47 (0.06)	0.47 (0.04)
**S-170m**	**28.41 (1.7)**	**275.07 (3.83)**	**43.79 (0.05)**	**62.69 (0.17)**	**60.59 (1.83)**	**89.79 (16.72)**	**23.7 (0.84)**		**14.16 (0.05)**	**355.21 (0.48)**	**34.65 (0.74)**	**195 (3.46)**	**23.83 (0.07)**	**165.67 (3.15)**	**17.76 (0.34)**	**43.89 (0.42)**	**5.99 (0.16)**	**26.88 (0.12)**	**6.71 (0.12)**	**2.11 (0.04)**	**7.08 (0.18)**	**1.06 (0.01)**	**6.59 (0.03)**	**1.31 (0.02)**	**3.61 (0.19)**	**0.57 (0.01)**	**3.18 (0.07)**	**0.51 (0.03)**	**4.89 (0.49)**	**1.41 (0.01)**	**1.71 (0.13)**	**1.52 (0.06)**	**0.48 (0.01)**
S-176-177m-01	25.8 (0.99)	252.66 (7.05)	68.33 (2.57)	180.18 (8.73)	49.93 (1.96)	101.93 (4.47)	20.09 (0.89)	1.39 (0.6)	12.51 (0.43)	306.56 (6.02)	31.08 (0.75)	178.12 (4.19)	22.67 (0.45)	150.68 (4.61)	15.99 (0.55)	40.96 (1.18)	5.29 (0.2)	25.36 (1.09)	5.75 (0.5)	2.07 (0.14)	5.55 (0.39)	0.91 (0.08)	5.68 (0.3)	1.2 (0.09)	3.34 (0.19)	0.5 (0.04)	2.83 (0.25)	0.46 (0.04)	3.98 (0.32)	1.36 (0.06)	1.91 (0.17)	1.14 (0.08)	0.41 (0.04)
S-176-177m-04	27.25 (0.87)	303 (5.2)	44.12 (0.98)	48.35 (1.77)	67.12 (4.54)	99.8 (3.49)	23.03 (0.68)		15.32 (0.41)	346.88 (4.98)	38.3 (0.67)	222.45 (3.99)	27.5 (0.45)	184.59 (4.07)	20.63 (0.43)	49.23 (0.86)	6.64 (0.16)	28.87 (0.91)	7.26 (0.38)	2.41 (0.11)	8.11 (0.34)	1.19 (0.05)	7.77 (0.29)	1.46 (0.06)	4.26 (0.16)	0.6 (0.04)	3.29 (0.19)	0.61 (0.03)	5.42 (0.22)	1.59 (0.05)	1.72 (0.12)	1.73 (0.09)	0.55 (0.04)
S-176-177m-10	26.53 (0.85)	283.89 (4.37)	40.78 (0.93)	56.58 (1.84)	55.42 (1.75)	94.19 (3.11)	23.98 (0.6)		15.26 (0.41)	372.33 (4.79)	39.06 (0.68)	217.36 (3.14)	25.84 (0.36)	187.94 (3.37)	20.14 (0.38)	48.19 (0.81)	6.49 (0.14)	28.4 (0.86)	7.44 (0.35)	2.45 (0.11)	7.98 (0.39)	1.25 (0.05)	7.16 (0.26)	1.37 (0.06)	3.98 (0.15)	0.59 (0.03)	3.68 (0.21)	0.52 (0.03)	5.21 (0.19)	1.74 (0.05)	1.97 (0.11)	1.75 (0.08)	0.47 (0.04)
**S-176-177m**	**26.53 (0.66)**	**279.85 (24.74)**	**51.07 (14.88)**	**95.04 (70.94)**	**57.49 (7.87)**	**98.64 (5.02)**	**22.36 (1.98)**		**14.36 (1.66)**	**341.92 (30.8)**	**36.15 (3.95)**	**205.98 (22.03)**	**25.34 (1.8)**	**174.4 (17.88)**	**18.92 (2.37)**	**46.13 (4.02)**	**6.14 (0.65)**	**27.54 (1.73)**	**6.81 (0.87)**	**2.31 (0.19)**	**7.21 (1.28)**	**1.12 (0.17)**	**6.87 (1)**	**1.34 (0.13)**	**3.86 (0.43)**	**0.56 (0.05)**	**3.27 (0.41)**	**0.53 (0.07)**	**4.87 (0.72)**	**1.57 (0.13)**	**1.87 (0.13)**	**1.54 (0.33)**	**0.48 (0.07)**

EPMA analyses from VUW, using 15 kV, 8 nA, 10 µm spot size. Each grain is average of n analyses, with 1 st dev in brackets
LA-ICPMS analyses from UofOtago, using 193 nm laser at 60 s count times. Each analysis is one spot, with 1 st dev (from counting statistics) in brackets
